# Targeting TGF‐β signaling, oxidative stress, and cellular senescence rescues osteoporosis in gerodermia osteodysplastica

**DOI:** 10.1111/acel.14322

**Published:** 2024-09-05

**Authors:** W. L. Chan, C. H. Bucher, J. Goldes, A. C. Ma, M. Steiner, B. M. Willie, S. Mundlos, U. Kornak

**Affiliations:** ^1^ Institut für Medizinische Genetik Und Humangenetik Charité‐Universitätsmedizin Berlin Berlin Germany; ^2^ Max‐Planck‐Institut für Molekulare Genetik, FG Development & Disease Berlin Germany; ^3^ BIH Center for Regenerative Therapies Charité‐Universitätsmedizin Berlin Berlin Germany; ^4^ Julius Wolff Institute of Biomechanics and Musculoskeletal Regeneration Charité ‐ Universitätsmedizin Berlin Berlin Germany; ^5^ Berlin‐Brandenburg School for Regenerative Therapies Charité ‐ Universitätsmedizin Berlin Berlin Germany; ^6^ Department of Health Technology and Informatics Hong Kong Polytechnic University Hong Kong China; ^7^ Research Centre Shriners Hospital for Children‐Canada Montreal Quebec Canada; ^8^ Department of Pediatric Surgery McGill University Montreal Quebec Canada; ^9^ Faculty of Dental Medicine and Oral Health Sciences McGill University Montreal Quebec Canada; ^10^ Institut für Humangenetik Universitätsmedizin Göttingen Göttingen Germany

**Keywords:** cellular senescence, gerodermia osteodysplastica, Gorab, osteoporosis, proteoglycan, reactive oxygen species, TGF‐beta

## Abstract

GORAB is a key regulator of Golgi vesicle transport and protein glycanation. Loss of GORAB function in gerodermia osteodysplastica (GO) causes shortening of glycosaminoglycan chains, leading to extracellular matrix disorganization that results in wrinkled skin, osteoporosis and elevated TGF‐β signaling. In this study, we investigated the role of TGF‐β‐signaling, oxidative stress, and resulting cellular senescence in the osteoporosis phenotype of GO. Treatment of *Gorab* ^*Prx1*^ conditional knockouts with the TGF‐β neutralizing antibody 1D11 rescued the trabecular bone loss, indicating that TGF‐β overactivation causes osteoporosis in GO. Using an inducible knockout system, we demonstrated that TGF‐β dysregulation was not a cell‐intrinsic effect of GORAB inactivation, but a consequence of a disorganized extracellular matrix. Enhanced TGF‐β signaling caused elevated *Nox4* expression in *Gorab* ^Prx1^ mutants and in GO patients' fibroblasts, resulting in overproduction of mitochondrial superoxide. The resulting oxidative stress was detected in GORAB null cells and also in wildtype bystander cells. The same effect was observed in zebrafish after TALEN‐mediated *gorab* inactivation, indicating that the pathway is evolutionarily conserved. Treating *Gorab* ^Prx1^ mutants with the antioxidant N‐acetylcysteine ameliorated the osteoporosis phenotype. TGF‐β induced oxidative stress coincided with accumulation of DNA damage and elevated expression of senescence markers. Inactivation of *Cdkn2a* in the *Gorab* ^*Prx1*^ rescued the osteoporosis phenotype. Reduced colony formation and altered subpopulations of bone marrow stromal cells were normalized upon inactivation of *Cdkn2a*, thus further demonstrating the relevance of cellular senescence in the pathogenesis. Our results shed light on the causative role of a TGF‐β‐Nox4‐senescence axis and therapeutic strategies for GO.

## Graphical abstract

Loss‐of‐function mutations of the gene GORAB in gerodermia osteodysplastica (GO) cause shortening of glycan chains in proteoglycans such as the small leucine rich proteoglycans (SLRPs). This results in the disorganization of collagen fibers, leading to defective osteoblast differentiation and overactivation of TGF‐ß signaling. The overactivation of TGF‐ß signaling induces oxidative stress, leads to accumulation of DNA damage and cellular senescence in the osteoblast lineage and also bone marrow stromal cells, leading to osteoporosis in GO.

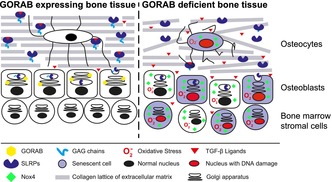

**Abbreviations**
B.Pm: Bone perimeter
bmMSCs: Bone marrow mesenchymal stromal cells
BrDU: Bromodeoxyuridine
BV: Bone volume
CFU: Colony formation unit
Ct.B.Ar: Cortical bone area
Ct.Th: Cortical thickness
ECM: Extracellular matrix
GAGs: Glycosaminoglycans
GO: Gerodermia osteodysplastica
IgG: Immunoglobulin G
N.Ob: Number of osteoblast
N.Oc: Number of osteoclast
N.Ot: Number of osteocyte
NAC: N‐acetylcysteine
SA‐βGal: Senescence associated beta‐galactosidase
SLRPs: Small leucin‐rich proteoglycans
TALEN: Transcription activator‐like effector nuclease
Tb.N: Trabecular number
Tb.Sp: Trabecular separation
Tb.Th: Trabecular thickness
TV: Tissue volume

## 1 INTRODUCTION

Proteoglycans are key components of the extracellular matrix (ECM) maintaining hydration of the ECM, organizing collagen fibril formation, and regulating the activity and availability of growth factors and ligands in the ECM (Kalamajski & Oldberg, 2010; Kim et al., 2011; Nikitovic et al., 2012). The length of the glycosaminoglycans (GAGs) on proteoglycans gets reduced during aging and impairs the structural integrity of the ECM (Chan et al., 2018; Grzesik et al., 2002; Li et al., 2013). Whether this GAG shortening is a cause or consequence of aging remains elusive. Gerodermia osteodysplastica (GO) is a rare segmental progeroid disorder characterized by wrinkled skin and osteoporosis. It is caused by loss‐of‐function mutations in the gene *GORAB* (Hennies et al., 2008). The GORAB protein functions as a scaffolding factor for the COPI coatomer. Loss of this protein causes defective COPI‐mediated retrograde trafficking of trans‐Golgi enzymes, leading to defective protein glycosylation and thus shortening of GAG chains on proteoglycans (Witkos et al., 2019), similar to age‐related ECM changes.

We previously created various mouse models with constitutive and tissue‐specific *Gorab* inactivation to study the pathomechanism of GO (Chan et al., 2018). In contrast to humans, constitutive inactivation of *Gorab* in mice resulted in perinatal lethality without a significant bone phenotype, but with strongly reduced protein glycosylation and shortening of GAG chains of proteoglycans such as decorin in bone and skin (Chan et al., 2018; Witkos et al., 2019). In vitro, differentiation and collagen secretion of *Gorab* null osteoblasts were not impaired, indicating that the bone pathology in GO is not due to a cell‐intrinsic osteoblast defect. Limb bud‐specific inactivation of *Gorab* in the long bones of *Gorab* ^*Prx1*^ mice recapitulated the bone phenotype of GO patients. *Gorab* ^*Prx1*^ mutants developed severe osteoporosis postnatally, with extreme thinning of the cortical bone and high fracture risk in the long bones (Chan et al., 2018). This was associated with significantly increased osteoblast and osteocyte numbers without changes in osteoclasts. The osteocytes showed a rounded morphology with disorganized canalicular network, indicating disorganized ECM caused by defective protein glycosylation in *Gorab* ^*Prx1*^. The disorganized ECM caused overactivation of TGF‐β signaling (Chan et al., 2018). TGF‐β signaling has been described as a key regulator of bone homeostasis, including disorders such as osteogenesis imperfecta (Grafe et al., 2014; Song et al., 2022; Wu et al., 2016). However, the role of overactivated TGF‐β signaling in the pathomechanism of GO remains unknown.

In this study, we utilized mouse models, patients' fibroblasts and a zebrafish model to investigate the causal relationship between impaired glycanation in the ECM, TGF‐β signaling, and cellular senescence in the GO pathomechanism and explored potential therapeutic interventions.

## 2 RESULTS

### 2.1 GORAB deficient long bones in *Gorab* ^*Prx1*^ mutants are more sensitive to TGF‐β inhibition than the *Gorab*‐expressing axial skeleton

**FIGURE 1:**
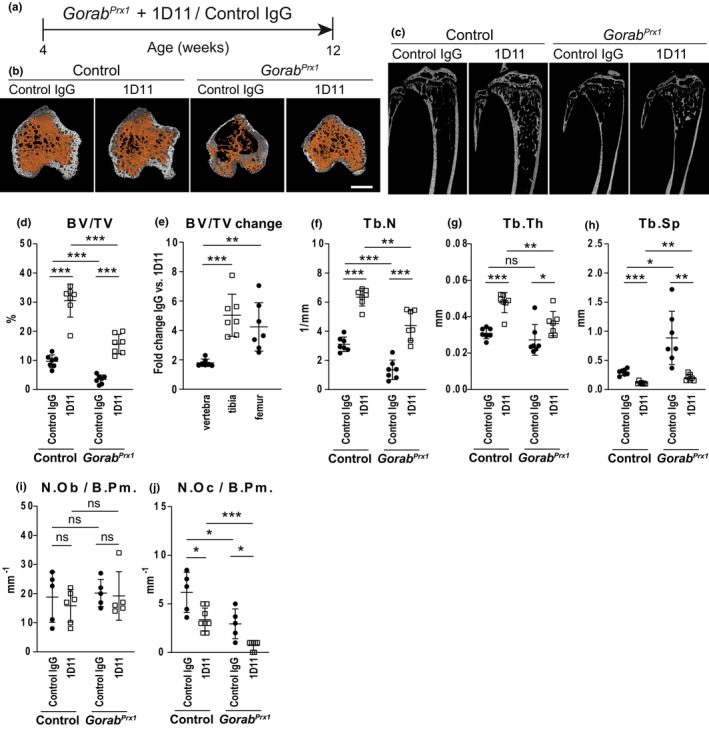
*Gorab*‐deficient long bones are more sensitive to TGF‐β inhibition than the *Gorab*‐expressing axial skeleton. (a) Treatment scheme for the *Gorab* ^*Prx1*^ mice. 4 weeks old *Gorab* ^*Prx1*^ mice were injected with 10 mg/kg body weight of either control IgG or 1D11 thrice a week by intraperitoneal injection for 8 weeks. (b) 3D rendering and (c) Representative microCT images showing the proximal tibia of control and *Gorab* ^*Prx1*^ mice treated with control IgG or 1D11. Scale bar = 500 μm. Quantitation of (d) percentage of trabecular bone volume (BV/TV); (e) comparison of rescue effects in vertebrae, tibia, and femur; (f) trabecular number (Tb.N); (g) trabecular thickness (Tb.Th); and (h) trabecular separation (Tb.Sp) in proximal tibia of control IgG and 1D11 treated control and *Gorab* ^*Prx1*^ mice (*N* = 7). Scanned and analyzed with Scanco μCT40 at 10 μm. Histomorphometric analysis at the proximal tibia secondary spongiosa in control and 1D11 treated *Gorab* ^*Prx1*^ mutants for (i) number of osteoblasts per bone perimeter (N.Ob/B.Pm); and (j) number of osteoclasts per bone perimeter (N.Oc/B.Pm). (*N* = 5–6). *p < 0.05, **p < 0.01, ***p < 0.001, ns, no statistically significant difference (unpaired Welch's *t*‐test).

In our previous study, we reported upregulated TGF‐β signaling in conditional *Gorab* ^*Prx1*^ mice, in which *Gorab* is inactivated in the limb bud mesenchyme (giving rise to the bones of the extremities), but not the axial skeleton comprising vertebrae and ribs (Chan et al., 2018). Therefore, we hypothesized that the overactivation of TGF‐β induced bone loss in GO. To test this hypothesis, we injected control IgG or anti‐TGF‐β antibody 1D11 (that inhibits all 3 TGF‐β ligands) thrice a week intraperitoneally into *Gorab* ^*Prx1*^ mutants and *Gorab* ^*floxed*^ control mice, starting at 4 weeks of age for 8 weeks (Figure 1a). Control IgG had no effect on the bone phenotype of neither controls nor *Gorab* ^*Prx1*^ mice. However, after 8 weeks of 1D11 treatment, the osteoporosis phenotype was significantly improved in the mutants as demonstrated by the increase in tibia and femur trabecular bone parameters to a level even higher than that of IgG treated control animals (Figure 1b–h, Figure S1). Histomorphometrical analysis detected no changes in osteoblast numbers but a significant reduction in osteoclast numbers (Figure 1i,j). This showed that the observed effect of 1D11 treatment was largely due to a reduction of osteoclast numbers as previously described, but also due to elevated trabecular thickness indicating higher bone formation (Santagati et al., 2005). Supporting this reasoning, the 1D11 treated control animals also showed a general increase in trabecular bone volume (Figure 1b–h, Figures S1 and S2a–d). This was also supported by the ~2‐fold increase in trabecular bone percentage in the 6th lumbar vertebrae of *Gorab* ^*Prx1*^ mutants, where Gorab was expressed normally (Figure 1e, Figure S2a–d). These data demonstrated a generalized promoting effect on trabecular bone by the 1D11 treatment through inhibition of osteoclast differentiation. However, the 1D11 treatment resulted in an average 4‐5‐fold increase in trabecular bone mass in the *Gorab* inactivated tibia and femur, respectively (Figure 1e). This indicated that the pathologically altered tissue was significantly more responsive to the 1D11 treatment, leading to a more pronounced positive effect comparing to the normal tissue. Taken together, our data demonstrate that overactivation of TGF‐β is a major contributor to osteoporosis in GO. Targeting this pathway by a TGF‐β inhibitory antibody can rescue trabecular bone loss in GO and is a potential therapeutic option.

### 2.2 Overactivation of TGF‐β signaling is not due to cell‐intrinsic effects of GORAB deficiency

Loss of GORAB causes defective protein glycosylation and reduces glycosaminoglycan content (Chan et al., 2018; Witkos et al., 2019). This effect was best exemplified by the changes in the glycanation of small leucin‐rich proteoglycans (SLRPs) such as decorin (Chan et al., 2018). Changes in the glycan chains of small SLRPs have been shown to be underlying hereditary genetic disorders such as Ehlers‐Danlos syndrome (Miyake et al., 2010; Quentin et al., 1990). The shortening of glycan chains in GO leads to an immature collagen network and thus disorganized ECM in bone, leading to porous cortical bone and defective osteocyte maturation (Chan et al., 2018).

The anti‐TGF‐β treatment with 1D11 showed no effect on femur and tibia length and cortical bone thickness of *Gorab* ^*Prx1*^ mice (Figures S1e,f and S3a,b). The cortical bone osteocyte number in the tibiae of *Gorab* ^*Prx1*^ was also not affected by 1D11 treatment (Figure S3c). The lack of changes in the cortical bone indicated that the 1D11 treatment had no effect on the ECM organization. This was supported by the lack of effect of 1D11 on the length of GAG chains of decorin in the bone of *Gorab* ^*Prx1*^ mice (Figure S4), suggesting that the overactivation of TGF‐β signaling is downstream of the impaired protein glycanation and defective ECM. This again supported that the overactivation of TGF‐β was secondary to the defective protein glycanation and ECM disruption caused by loss of GORAB. In order to further test this hypothesis, we employed a tamoxifen‐inducible *Gorab* knockout mouse model (*Gorab* ^*icre*^) generated by crossing the conditional *Gorab* ^flox^ mouse model (Chan et al., 2018) with a transgenic mouse line constitutively expressing the Cre‐ERT2 fusion protein in all tissues, allowing for tamoxifen‐mediated gene inactivation (Santagati et al., 2005). This model allows the discernment of cell‐intrinsic impacts of *Gorab* inactivation from cell‐extrinsic factors like a disorganized ECM. Tamoxifen was administered thrice per week at the age of 10 weeks and the mice were analyzed 2 weeks after the last tamoxifen injection (Figure S5a). The efficiency of *Gorab* inactivation in cortical bone was comparable to the reported level of *Gorab* inactivation in *Gorab* ^*Prx1*^ mice (~60%) (Chan et al., 2018) (Figure S5b). Our results demonstrated that loss of GORAB after formation of a normal ECM had no immediate effect on expression of TGF‐β responsive genes in vivo, the glycanation state of decorin or bone microarchitecture (Figure S5b–h). Likewise, inactivation of *Gorab* in *Gorab* ^*icre*^ primary calvaria osteoblasts also showed no significant changes in expression of TGF‐β responsive genes in vitro within 3 days of culture (Figure S5i). Taken together, our results showed that inactivation of Gorab in 10 weeks old mice, which have a functional ECM and properly glycanated proteoglycans, showed no effect on bone microarchitecture and TGF‐β signaling after 2 weeks. Therefore, the upregulated TGF‐β signaling in GO is not a direct, cell‐intrinsic consequence of *Gorab* inactivation, but a secondary effect caused by the reduced GAG content and disorganized ECM in GO.

### 2.3 GORAB deficiency leads to TGF‐β induced oxidative stress as an evolutionarily conserved mechanism

The effects of altered TGF‐β signaling are highly context‐specific and therefore difficult to predict (Treiber et al., 2011). In order to understand how elevated TGF‐β might contribute to osteoporosis in GO, we performed a transcriptome screening for differentially expressed genes. 600 protein‐coding transcripts with relevant read counts were at least ±0.5 Log_2_ up‐ and 19 downregulated in bone tissue from *Gorab* ^*Prx1*^ compared to control animals (Table S1). Among the upregulated genes was *Nox4*, encoding an enzyme localized to mitochondria involved in the generation of superoxide causing oxidative stress (Block et al., 2009). This mechanism is a key mediator of the effect of TGF‐β signaling in pathologies such as muscle weakness in bone metastases (Waning et al., 2015), epithelial‐mesenchymal transition in pancreatic adenocarcinoma (Hiraga et al., 2013), and tissue fibrosis (Jain et al., 2013). Increased oxidative stress is correlated with age‐related bone loss (Treiber et al., 2011), while inactivation of *Nox4* limits bone loss (Goettsch et al., 2013). Therefore, we hypothesized that oxidative stress caused by *Nox4* upregulation mediates the effect of excess TGF‐β signaling in GO.

**FIGURE 2:**
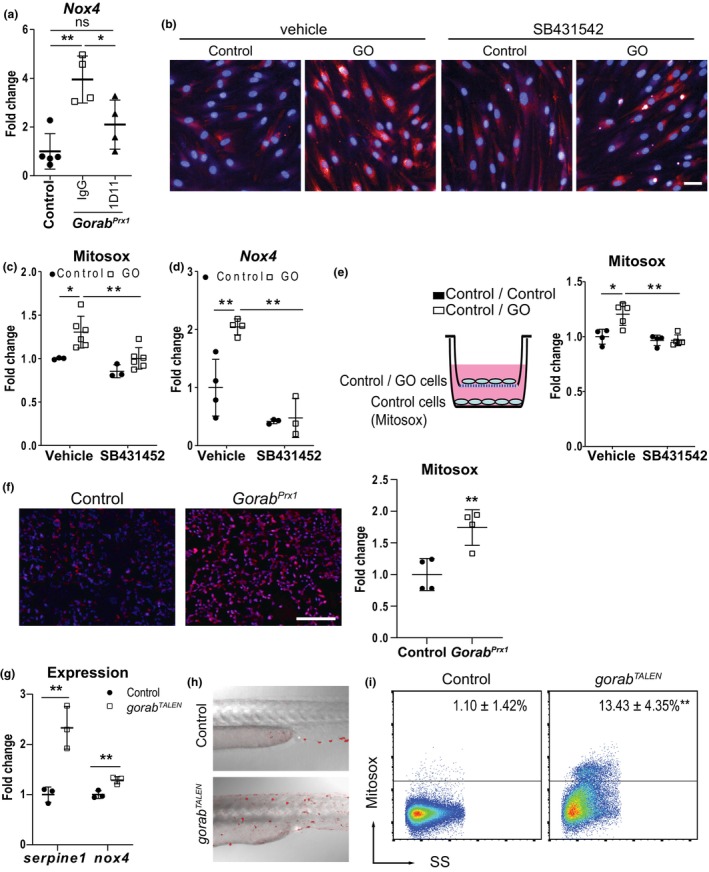
Elevated TGF‐β signaling caused increased Nox4 expression and elevated oxidative stress in GO. (a) 1D11 treatment inhibited the Nox4 upregulation in *Gorab* ^*Prx1*^ mice (*N* = 5) (b) Representative images and (c) quantitation of Mitosox intensity in GO patient fibroblasts (*N* = 3–6) treated with TGF‐β inhibitor SB431542. (d) SB431542 reduced Nox4 expression in GO patient fibroblasts (*N* = 3–4). (e) Coculturing of control fibroblasts with GO patient fibroblasts caused upregulation of mitosox signal in the control fibroblasts, which can be prevented by SB431542 (*N* = 4–5). (f) Increased in mitosox signal in the bone marrow of tibiae of 4 weeks old *Gorab* ^*Prx1*^ (N = 4) in vivo. (g) Knockdown of gorab in zebrafish by TALEN resulted in upregulation of serpine1 and nox4 expression. (h) Representative picture and (i) quantitation of Mitosox positive cells by flow cytometry in *gorab* *^TALEN^* zebrafish embryos. *p < 0.05, **p < 0.01, ns, no statistically significant difference (unpaired Welch's *t*‐test). Scale bar = 100 μm.

We found that 1D11 treatment in the *Gorab* ^*Prx1*^ mice reduced the expression of *Nox4* in bone to a level comparable to control mice (Figure 2a). This indicated that the upregulation of *Nox4* expression in *Gorab* ^*Prx1*^ was caused by TGF‐β signaling in vivo. Next, we examined the level of oxidative stress in *GORAB‐deficient* skin fibroblasts from GO patients in vitro. Fibroblasts were stained with Mitosox™, which gives out red fluorescence upon oxidation by superoxide and thus allows for quantitation of cellular superoxide‐induced oxidative stress. The GO patients' fibroblasts showed increased fluorescence intensities, indicating increased mitochondrial superoxide and oxidative stress which could be reduced to control levels by treatment with the specific TGF‐β signaling receptor inhibitor SB431452 (Halder et al., 2005) (Figure 2b,c). In contrast, the Mitosox™ signal of control cells was not affected by SB431452. The increase in oxidative stress levels coincided with the increase in the expression level of *Nox4* (Figure 2d) in GO cells, which were both reduced after TGF‐β inhibitor treatment. This indicated that the upregulation of *Nox4* expression is responsible for the oxidative stress in GO cells and is caused by aberrant TGF‐β signaling. Since TGF‐β is secreted, we hypothesized that overactivation of TGF‐β might have a cell‐extrinsic effect on nearby cells. Therefore, we explored the effect of coculturing GO patients' fibroblasts on oxidative stress levels in control fibroblasts. Culturing control or GO patients' fibroblast in a culture well insert with 1 μm pore size allowed soluble molecules, but not cells, to pass through the membrane. Coculturing with GO fibroblasts induced mitochondrial superoxide production in control fibroblasts, which was blocked by addition of SB431452 (Figure 2e). This demonstrated that the effect of excess TGF‐β on inducing oxidative was not limited to GORAB null cells, but extended to nearby control cells. Indeed, this was supported by upregulated Mitosox™ signals in the bone marrow of *Gorab* ^*Prx1*^ tibiae in vivo (Figure 2f), which was a mixture of *Gorab* null and wildtype cells. This demonstrated that inactivation of GORAB induces oxidative stress in an auto‐ and paracrine fashion through TGF‐β signaling.

In order to see whether TGF‐β‐induced oxidative stress caused by *Gorab* inactivation is evolutionarily conserved, we disrupted *gorab* in zebrafish by TALEN (Figure S6). Similar to mice and humans, the TGF‐β responsive genes *serpine1* and n*ox4* were also upregulated in the *gorab* ^*TALEN*^ mutants (Figure 2g). The mutant zebrafish also showed elevated superoxide levels in vivo compared to controls (Figure 2h,i). Taken together, loss of GORAB resulted in elevated oxidative stress through TGF‐β induced *Nox4* upregulation and this effect is evolutionarily conserved in human, mouse, and zebrafish.

### 2.4 TGF‐β‐induced oxidative stress contributes to osteoporosis in *Gorab* ^*Prx1*^ mice

**FIGURE 3:**
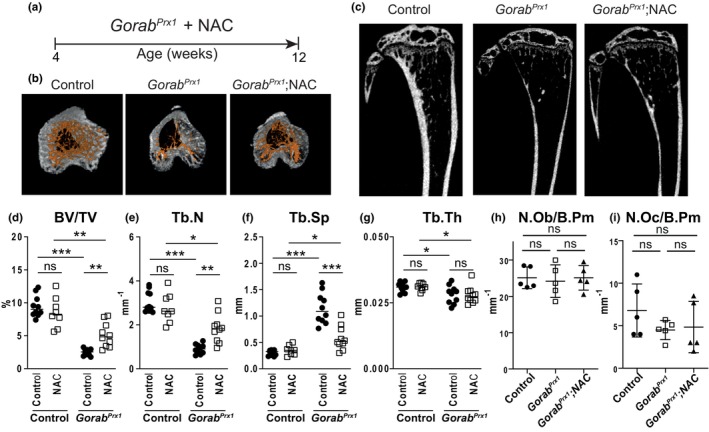
Antioxidant treatment ameliorated osteoporosis in *Gorab* ^*Prx1*^ mutants. (a) NAC treatment plan. 4 weeks old *Gorab* ^*Prx1*^ mutants started drinking 1 mg/mL of the antioxidant N‐acetylcyteine (NAC) or vehicle for 8 weeks. (b) 3D rendering, (c) representative microCT images and quantitation of (d) percentage of trabecular bone volume (BV/TV); (e) trabecular number (Tb.N); (f) trabecular separation (Tb.Sp); and (g) trabecular thickness (Tb.N) at the proximal tibia of 12 weeks old female control and *Gorab* ^*Prx1*^ mice treated with NAC (*N* = 8–10). Scale bar = 500 μm. Scanned and analyzed with Scanco μCT40 at 10 μm. Histomorphometric analysis at the proximal tibia secondary spongiosa in control and NAC treated *Gorab* ^*Prx1*^ mutants for (h) number of osteoblast per bone perimeter (N.Ob/B.Pm) and (i) number of osteoclast per bone perimeter (N.Oc/B.Pm). (*N* = 5) *p < 0.05, **p < 0.01, ***p < 0.001, ns, no statistically significant difference (unpaired Welch's *t*‐test).

To corroborate that oxidative stress contributed to the osteoporosis phenotype in GO, we explored the effects and the widely used antioxidant N‐acetylcysteine (NAC). We treated 4 weeks old *Gorab* ^Prx1^ mutants with 1 mg/mL NAC in drinking water for 8 weeks (Figure 3a). The treatment resulted in a significant improvement of the tibial and femoral trabecular bone parameters, but had no effect in control animals nor in the spine of the *Gorab* ^*Prx1*^ mice, in which *Gorab* is not inactivated (Figure 3b–g, Figures S7a–d and S8a–d). The degree of improvement of the trabecular bone after NAC treatment was milder than that of 1D11 treatment. In contrast to the 1D11 treatment, there were no significant changes in the number of osteoblasts and osteoclasts (Figure 3h,i). On the contrary, there were no changes in tibia and femur cortical bone thickness, bone length, or tibia cortical bone osteocyte numbers after NAC treatment (Figures S7e,f and S9a–c). This implied a lack of rescue of ECM in *Gorab* ^*Prx1*^ animals after NAC treatment. This was supported by absent changes in decorin glycanation after NAC treatment (Figure S4). The similarities in the rescue effects suggest that the 1D11 and NAC treatments targeted a common pathway in which excessive TGF‐β‐induced oxidative stress is a key mediator of osteoporosis in GO.

### 2.5 Evidence for DNA damage and cellular senescence in GORAB deficient cells and bone tissue

**FIGURE 4:**
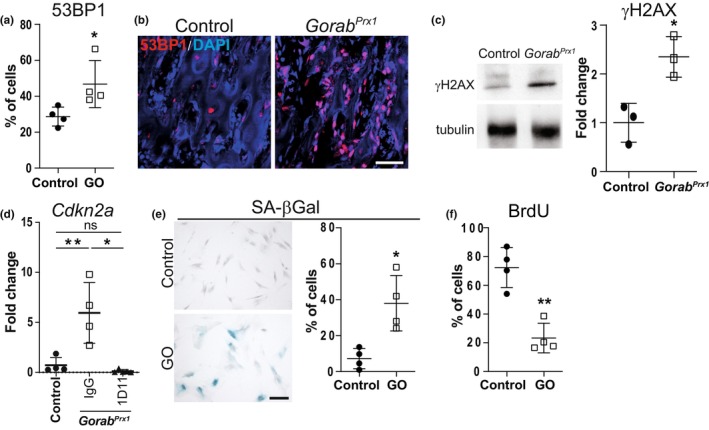
Evidence of DNA damage and cellular senescence in GORAB‐deficient cells and bone tissue. (a) Percentage of cells with 53BP1 nuclear foci in GO patient fibroblasts (*N* = 4). (b) Representative images of nuclear 53 bp1 staining in the secondary spongiosa of proximal tibia in 4 weeks old *Gorab* ^*Prx1*^ mice. Scale bar = 50 μm. (c) Increase in γH2AX expression in femur diaphysis of 4 weeks old *Gorab* ^*Prx1*^ mice (*N* = 3). (d) Relative expression of Cdkn2a in control and *Gorab* ^*Prx1*^ mice treated with control IgG or 1D11 (*N* = 5). (e) Representative images and quantitation of senescence associated beta‐galactosidase (SA‐βGal) positive cells in GO patient fibroblasts (*N* = 4). Scale bar =20 μm. (f) BrdU positive cells in Control and GO patient fibroblasts after 24 h BrdU labeling (*N* = 4). *p < 0.05, **p < 0.01, ns, no statistically significant difference (unpaired Welch's *t*‐test).

Superoxide radicals as detected by the Mitosox™ dye are known to cause DNA damage (Brauer et al., 2023). This damage can range from individual oxidized bases to single‐ and double‐strand breaks (DSB). DSB are repaired by a machinery comprising the proteins 53BP1 and γH2AX. Compared to control cells, cultured GORAB deficient fibroblasts showed an increased percentage of cells with 53BP1 foci, indicating accumulation of DSB (Figure 4a). In the *Gorab* ^*Prx1*^ mutants, we detected increased nuclear 53BP1 staining in osteoblasts and osteocytes (Figure 4b). γH2AX band intensity was enhanced in immunoblots of lysates from *Gorab* ^*Prx1*^ bone tissues compared to controls (Figure 4c), indicating accumulation of DNA damage.

Prolonged DNA damage triggers cell cycle arrest and cellular senescence through the p53 and RB tumor suppressor proteins (Campisi, 2005). Among the upregulated genes in bone tissue from *Gorab* ^*Prx1*^ mice were several markers for cellular senescence (Table S1), among them *Cdkn1a* and *Cdkn2a*, encoding the cyclin D kinase inhibitors p21 and p16, respectively (Figure S10) (Chan et al., 2018). As a demonstration that this overexpression was TGF‐β dependent, 1D11‐treatment drastically reduced *Cdkn2a* expression in *Gorab* ^*Prx1*^ (Figure 4d), indicating that cellular senescence in *Gorab* ^*Prx1*^ was caused by excessive TGF‐β signaling. In GO patients' fibroblasts, we detected an increase in senescence‐associated β‐galactosidase (SaβGal) activity (Figure 4e), demonstrating cell cycle arrest in GO patients fibroblasts. This was further supported by reduced proliferation of GO fibroblasts indicated by low rates of BrdU labeling (Figure 4f). These data suggest that loss of GORAB results in DNA damage and cellular senescence in both human and mouse; and inhibition of TGF‐β signaling prevents the cells from entering senescence.

### 2.6 Preventing cellular senescence by inactivating *Cdkn2a* rescues trabecular bone loss in *Gorab* ^*Prx1*^ mutants

Cellular senescence has been demonstrated to contribute to osteoporosis (Farr et al., 2017). In order to investigate the role of cellular senescence in the GO bone phenotype, we crossed the *Gorab* ^*Prx1*^ mouse line with *Cdkn2a* null mice. Inactivation of *Cdkn2a* prevents the cells from entering cellular senescence through the p16/pRB pathway (Krimpenfort et al., 2001). Again, the spines of the *Gorab* ^*Prx1*^ mutants served as an internal control allowing us to study the effects of absence of p16‐mediated cellular senescence in osteoporotic long bones versus the non‐affected spine.

**FIGURE 5:**
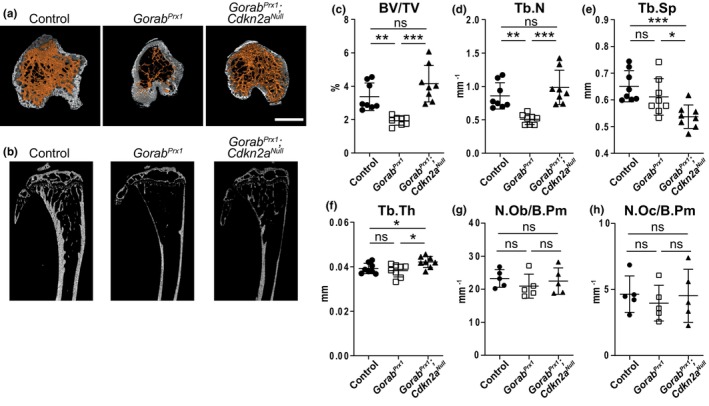
Preventing cellular senescence by inactivating p16 rescues trabecular bone loss in *Gorab* ^*Prx1*^ mutants. (a) 3D rendering, (b) representative microCT images and quantitation of (c) percentage of trabecular bone volume (BV/TV); (d) trabecular number (Tb.N); (e) trabecular separation (Tb.Sp); and (f) trabecular thickness (Tb.Th) at the proximal tibia of female control, *Gorab* ^*Prx1*^ and *Gorab* ^*Prx1*^;*Cdkn2a* ^*Null*^ mice at 12 weeks of age (*N* = 8). Scanned and analyzed with Bruker Skyscan 1172 at 5 μm. Scale bar = 500 μm. Histomorphometric analysis at the proximal tibia secondary spongiosa in control, *Gorab* ^*Prx1*^ and *Gorab* ^*Prx1*^;*Cdkn2a* ^*Null*^ mice for (g) number of osteoblast per bone perimeter (N.Ob/B.Pm) and (h) number of osteoclast per bone perimeter (N.Oc/B.Pm) (*N* = 5). *p < 0.05, **p < 0.01, ***p < 0.001, ns, no statistically significant difference (unpaired Welch's *t*‐test).

At 12 weeks of age, the genetic inactivation of *Cdkn2a* completely rescued the loss of trabecular bone in the tibiae of *Gorab* ^*Prx1*^ mutants, which was restored to the level of control animals (Figure 5a–f). The same rescue effect was also observed in the femur of the animals (Figure S11a–d). However, there was no difference in trabecular bone architecture in vertebrae when comparing *Gorab* ^*Prx1*^;*Cdkn2a* ^*Null*^, *Gorab* ^*Prx1*^, and control animals (Figure S12a–d). This was expected as the Prx1‐cre is not active in the spine and thus GORAB was functional. This demonstrated that the inactivation of *Cdkn2a* had no general effect on bone remodeling and architecture in wildtype bones. Our data illustrates that inhibition of cellular senescence prevented the trabecular bone loss in the long bones of *Gorab* ^*Prx1*^ mice and highlighted the crucial role of p16‐mediated cell cycle arrest in osteoporosis of GO. Interestingly, no significant changes in osteoblast and osteoclast numbers were found in the mutants at 12 weeks of age (Figure 5g,h). Prevention of cellular senescence in *Gorab* ^*Prx1*^ mutants by inactivation of *Cdkn2a* had no effect on tibia and femur cortical bone thickness and bone length (Figures S11e,f and S13a,b), cortical osteocyte numbers (Figure S13c), or glycanation of decorin (Figure S4). This suggested that cellular senescence is secondary to ECM changes after Gorab inactivation and the complete rescue of the trabecular bone phenotype in the *Gorab* ^*Prx1*^;*Cdkn2a* ^*Null*^ mutants indicated that cellular senescence was a major effector of trabecular bone loss in the pathology of GO.

### 2.7 Alteration in mesenchymal stem cell subtypes underline the senescence‐induced osteoporosis in GO

**FIGURE 6:**
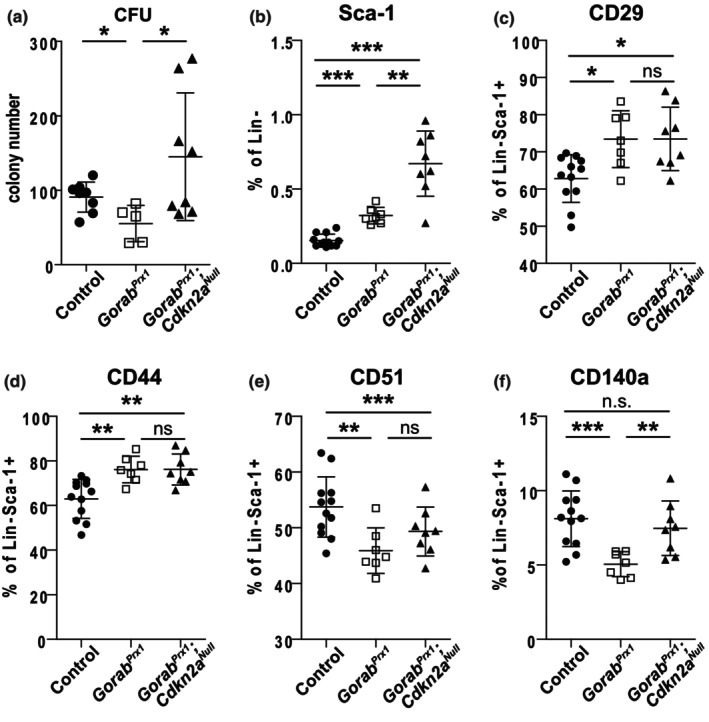
Alteration of mesenchymal stem cell subpopulations in *Gorab* ^*Prx1*^ mutant. (a) Number of Colony Formation Units (CFU) from the bone marrow stromal cells of 12 weeks old Control (*N* = 8), *Gorab* ^*Prx1*^ (*N*=) and *Gorab* ^*Prx1*^;*Cdkn2a* ^*Null*^ (*N* = 8) mice. (b) Flow cytometry analysis of mesenchymal stem cells (mMSCs) defined as Lin‐ (CD45‐CD11b‐CD34‐Cd31‐) Sca‐1+. Flow cytometry analysis of mMSCs subpopulations as (c) mMSCs CD29+; (d) mMSCs CD44+; (e) mMSCs Cd51+ and (f) mMSCs CD140a+. *N* = 12 Control, *N* = 7 *Gorab* ^*Prx1*^, *N* = 8 *Gorab* ^*Prx1*^;*Cdkn2a* ^*Null*^. *p < 0.05, **p < 0.01, ***p < 0.001, ns, no statistically significant difference (unpaired Welch's *t*‐test).

Our next question was how cellular senescence may contribute to the pathomechanism of GO. The remodeling of trabecular bone is performed by osteoblasts and osteoclasts originating from the bone marrow cell pool. Previously, we reported alterations in osteoblast differentiation and function that underly the reduced bone formation in 4‐week‐old *Gorab* ^*Prx1*^ mice (Chan et al., 2018). We hypothesized that bone marrow mesenchymal stromal cells (bmMSCs), precursor cells of osteoblasts, are altered in the *Gorab* ^*Prx1*^ mutants. We therefore performed a colony formation unit (CFU) assay for bmMSCs, which revealed a significantly reduced number of CFU in 12‐week‐old *Gorab* ^*Prx1*^ mutants (Figure 6a). This suggested a reduced proliferation potential due to enhanced cellular senescence in the mutant cells. Indeed, the CFU of bmMSCs from *Gorab* ^*Prx1*^;*Cdkn2a* ^*Null*^ double mutants was significantly higher than that of *Gorab* ^*Prx1*^ mutants and comparable to that of control animals (Figure 6a). This indicated that cellular senescence causes reduction in the proliferation potential of bmMSCs in GO. We further characterized the subpopulations of bmMSCs in the mutants based on cell surface markers by flow cytometry. The percentage of Lin‐ (Lineage marker, CD45‐CD11b‐CD34‐CD31‐) Sca‐1+ bmMSCs was significantly higher in the 12‐week‐old *Gorab* ^*Prx1*^ mice than control animals (Figure 6b). The inactivation of *Cdkn2a* resulted in a ~2‐fold increase in the percentage of Lin‐ Sca‐1+ bmMSCs in the *Gorab* ^*Prx1*^ mice (Figure 6c), coinciding with the rescue of CFU in the *Gorab* ^*Prx1*^;*Cdkn2a* ^*Null*^ mice (Figure 6d). We performed further analysis of bmMSC subtypes based on CD29, CD44, CD51, and CD140a expression. We found a significant increase in the CD29+ bmMSCs and CD44+ bmMSCs populations in the *Gorab* ^*Prx1*^ mutants compared to controls, while there was a decrease in CD51+ bmMSCs (mouse skeletal stem cells) (Chan et al., 2015) and CD140a/Pdgfra+ bmMSCs (PαS cells) (Houlihan et al., 2012; Sivaraj et al., 2021) (Figure 6e,f). These two cell populations were shown to contribute to osteoblast formation in vivo. Taken together, our data demonstrated an alteration of bmMSC subpopulations in the bone marrow of *Gorab* ^*Prx1*^ mutants, with a decrease in bmMSC proliferation potential and a decrease in osteogenic progenitors. Inactivation of *Cdkn2a* showed no significant impact on the number of CD29+, CD44+ or CD51+ bmMSCs but resulted in a significant increase in the PαS bmMSCs to a level comparable to controls. Thus, loss of *Gorab* causes cellular senescence in the bone marrow mesenchymal stromal cells and affects the bmMSC subtype composition, in particular the PαS bmMSCs that likely contribute to the imbalance in bone remodeling and thus osteoporosis.

## 3 DISCUSSION

Upregulation of TGF‐β plays critical a role in multiple disorders and age‐related pathologies. In bone metastases (Waning et al., 2015) and osteogenesis imperfecta (Grafe et al., 2014), excessive TGF‐β signaling is coupled with oxidative stress or upregulation of senescence markers, respectively, which may ultimately converge on the same pathways and downstream effects on cell functions (Grafe et al., 2014; Waning et al., 2015). Impaired ECM integrity is a common upstream trigger for TGF‐β, which can be secondary to mutations in ECM proteins, factors involved in their processing or, as in the case of GO, reduced glycanation of ECM proteins (Chan et al., 2018). Glycanation within bone tissue was also reduced with increasing age. In contrast to selective cell cycle inhibition by p16 or p21 overexpression, DNA damage‐induced cellular senescence leads to reduced glycosaminoglycan synthesis (Brauer et al., 2023). This underlines the crucial role of oxidative stress‐induced damage in the proposed TGF‐β‐induced senescence pathway and implies a feedback loop that keeps cells in a senescent state. It also suggests that TGF‐β induced senescence could contribute to age‐related bone loss, demonstrating the broad physiological implications of our findings. The similarities in the rescue effect between anti‐TGF‐β treatment, antioxidant treatment, and inhibition of cellular senescence supported that the targeted mechanisms belong to one common pathway.

Moreover, the complete rescue of trabecular bone in the long bones, but not in the vertebrae, of *Groab* ^*Prx1*^;*Cdkn2a* ^*Null*^ double mutants strongly indicates that cellular senescence is a major effector of the excessive TGF‐β signaling pathway in this bone compartment. During growth, the amount of trabeculae is mainly determined by the activity of the metaphyseal growth plate and resorption of the primary spongiosa by osteoclasts. Given that the 1D11 treatment of *Gorab* ^*Prx1*^ mutants did not change longitudinal bone growth, our results hint at the inhibition of osteoclast activity as the basis for the normalization of trabecular bone mass (Figure 1j), although osteoclast numbers were not elevated in *Gorab* ^*Prx1*^ trabecular bone (Figures 1j, 3i, 5h) or in a GO bone biopsy (Chan et al., 2018).

We found evidence for TGF‐β‐induced senescence in bone marrow stromal cells in *Gorab* ^*Prx1*^ mice, especially in the Lin‐Sca1 + Pdgfrα+ (PαS) population, which was previously shown to express high levels of *Cdkn2a* in aged mice (Yoshida et al., 2012). Senescent osteoblast progenitors, osteoblasts, and osteocytes have been found to increase with age (Farr et al., 2016) and can be targeted by senolytics (Doolittle et al., 2023). Senolytic treatment killing senescent cells has been shown to be effective in ameliorating osteoporosis (Farr et al., 2017), improve physical function and increase lifespan in aged mice (Xu et al., 2018). This demonstrated the importance of senescent osteoblasts and osteoprogenitor cells in trabecular bone loss, which coincided with our findings after *Cdkn2a* inactivation to prevent cells from entering senescence. Prevention of senescence by *Cdnk2a* inactivation is fundamentally different from senolytic treatment. It prevents cells from entering senescence and showed significant rescue effects in *Gorab* ^*Prx1*^ at the expense of weakening cell cycle inhibition as a major cancer defense mechanism (Sharpless et al., 2001). The resulting increased cancer risk reduces the therapeutic potential of *Cdkn2a* inhibition in contrast to senolytic treatment, which removes senescent cells and does not promote cancer development (Kirkland et al., 2017). Further experiments are ongoing to explore the potential of senolytic treatment in GO.

The thinning of the cortical bone and increased cortical bone osteocyte numbers in *Gorab* ^*Prx1*^ mice were not responsive to the treatments applied in this study, indicating that the cortical bone phenotype is not caused by TGF‐β overactivation at the investigated age of 8–12 weeks. However, this does not exclude a role of the TGF‐β pathway in the cortical bone anomalies during earlier developmental stages as indicated by elevated phosphorylation of the downstream signaling factor Smad2 in this bone compartment at 4 weeks of age (Chan et al., 2018). Increased P‐Smad2 coincided with elevated *Sp7* expression, which is known to be directly regulated by TGF‐β and has the potential to increase osteocyte numbers (Wu et al., 2016; Yoshida et al., 2012).

TGF‐β inhibition with the antibody Fresolimumab (a human version of 1D11), showed promising results in a phase 1 clinical trial for the treatment of patients with osteogenesis Imperfecta (Song et al., 2022). The application of Fresolimumab can potentially be extended to GO and other low bone mass disorders that directly or indirectly affect the ECM, and result in increases TGF‐β signaling activity. Although cellular senescence induced by TGF‐β signaling and oxidative stress have been demonstrated in various in vitro systems (Hubackova et al., 2012; Wu et al., 2014), this study is the first to demonstrate the pathophysiological relevance of this pathway in a hereditary disorder with early‐onset osteoporosis.

In summary, our data demonstrate that upregulation of TGF‐β is a secondary effect of the ECM disorganization in GO. The overactivation of TGF‐β caused oxidative stress and DNA damage that ultimately leads to cellular senescence. Targeting this TGF‐β‐oxidative‐stress‐cellular senescence pathway by inhibition of TGF‐β signaling, antioxidant treatment, or *Cdkn2a* inactivation can ameliorate the osteoporosis phenotype in GO. Our results validate the crucial role and therapeutic potential of this pathway in the GO pathology and shed light on how loss of protein glycanation in aging can impinge on the function of bone and connective tissue cells.

## 4 MATERIALS AND METHODS

### 4.1 Animals

All analyses were done on female mice homozygous for the conditional *Gorab* ^*flox*^ allele and heterozygous for the Prx1‐cre transgene (*Gorab* ^*Prx1*^). Homozygous conditional *Gorab* ^*flox*^ mice without the cre allele from the same generation served as controls. All animals had been backcrossed with C57/Bl6 mice for at least 5 times. 1D11 (Cat#BP0057, Bioxcell, New Hampshire, USA) treatment was done by intraperitoneal injection of 10 mg antibody per kg body weight thrice a week from 4 to 12 weeks of age. Antioxidant treatment was done by adding with 1 mg/mL N‐acetyl cysteine (Cat#A9165, Sigma‐Aldrich, Munich, Germany) to the drinking water from 4 to 12 weeks of age. All animal experimental procedures were approved by the ‘Landesamt für Gesundheitsschutz und Technische Sicherheit (LaGeTSi), Berlin, Germany (approval number G0213/12, G0138/16).

### 4.2 microCT analysis

microCT analysis for the 1D11 and NAC treatment were done using the Scanco μCT40 (Scanco Medical, Brüttisellen, Switzerland). The scanning was performed at 10 μm voxel size. The volume of interest for trabecular bone measurement was a region of 700 μm at the secondary spongiosa of the tibia. 1 mm of cortical bone was measured at a region 1200 μm below the proximal growth plate of the tibia and above the distal growth plate of femur. microCT analysis of *Gorab* ^*icre*^ and *Gorab* ^*Prx1*^;*Cdkn2a* ^*Null*^ mutants and the corresponding Control and *Gorab* ^*Prx1*^ mice were scanned with a Skyscan 1172 (Bruker Corp., Massachusetts, USA). The scanning was done at 5 μm voxel size at a region 300 μm below the growth plate for a volume of 10% of the bone length. Cortical bone was measured at a region of 5% bone length, starting from 15% bone length below the growth plate. The trabecular bone volume of the sixth lumbar vertebra of all animals was determined by measuring the trabecular bone between the two growth plates of the vertebra.

### 4.3 Histology

Undecalcified mouse tibiae from 12‐week‐old mice were first fixed in 4% paraformaldehyde and subsequently embedded in Methylmethacrylate, MMA (Cat#00834, Polysciences, Eppelheim, Germany) and then sectioned for histological study. The embedded samples were then sectioned using a Leica RM2255 microtome (Leica, Wetzlar, Germany) at 5 μm thickness and subjected to Von Kossa/Van Giesson staining, Von Kossa/hematoxylin staining, Goldner trichrome staining, or Pircosirius red staining.

### 4.4 Histomorphometry

The tibiae of the animals were fixed in 4% PFA and embedded in MMA as mentioned previously and then sectioned at 5 μm thickness. Sections were then stained with 0.1% Toluidine Blue and the number of osteoblast, osteoclast, and osteocyte were counted according to standard protocols using the Osteomeasure™ histomorphometry system (Osteometrics, Atlanta, USA).

### 4.5 Expression analysis

Mouse tibiae were first dissected, removing attached muscles and the fibula. Then the epiphyses were cut off and the bone marrow was flushed out with RNAlater® (Thermo Fisher, Germany). The cleared bone shaft was frozen in liquid nitrogen and then homogenized with pestle and mortar. RNA was extracted from the homogenized tissue with Aurum Total RNA Fatty and Fibrous Tissue kit (Biorad, Mississauga, Canada). RNA quality was confirmed by Bioanalyzer (Agilent Technologies, USA). RNAseq was done following the NEBNext Poly(A) mRNA Magnetic Isolation Module (New England Biolabs, USA) protocol and HighSeq 1500 (Illumina, San Diego, USA) sequencing of around 50.000 reads/sample. RNAseq data processing was executed by the Canadian Centre for Computational Genomics using the in‐house MUGQIC pipeline. Read trimming was performed using Trimmomatic (Bolger et al., 2014) and reads were aligned by STAR (Dobin et al., 2013). Differential expression was estimated by DEseq (Anders & Huber, 2010). Pathway enrichment was assessed with the help of PANTHER (Thomas et al., 2022).

For analysis of individual candidate genes RNAs were reverse‐transcribed using the RevertAidTM H Minus First Strand cDNA Synthesis Kit (Life Technologies, Darmstadt, Germany) with random hexamer primers. Quantitative PCR was performed with SYBR green (Life Technologies, Darmstadt, Germany) on ABI Prism 7500 (Life Technologies, Darmstadt, Germany). Expression levels were normalized to Gapdh expression. Primer sequences are shown in Table S1.

### 4.6 Immunostaining

Cell lysates from in vitro cultures were extracted with RIPA buffer followed by sonication. Mouse tissue lysates were extracted with 8 M Urea, 1% SDS followed by sonication. Mouse bone biopsies were first fixed in 4% PFA and then decalcified in Morses' solution (10% Sodium Citrate, 20% Formic Acid). The decalcified bone were then embedded in paraffin and sectioned for immunostainings. Antibodies used are as follows: Anti‐53BP1 (#sc22760, Santa Cruz, Heidelberg, Germany), Anti‐H2AX (#9718, Cell Signaling, Leiden, The Netherlands), Anti‐Gapdh (#sc6215, Santa Cruz, Heidelberg, Germany), and Anti‐DCN (#AF143, R&D, Abingdon, UK). For immunofluorescence on sections, the Tyramide signal amplification system (Perkin Elmer, Baesweiler, Germany) was used for signal development.

### 4.7 Mitochondrial superoxide measurement

Human skin fibroblast and mouse primary osteoblast were cultured in DMEM medium with 10% fetal calf serum, 1% penicillin/streptomycin, and 1% ultraglutamine. All cell culture medium and supplements were obtained from Lonza, Verviers, Belgium. Cells were cultured at full confluence for 7 days, with or without 10 μM SB431452 (Halder et al., 2005) (Sigma‐Aldrich, Munich, Germany). The cells were then stained with MitoSOX™ red mitochondrial superoxide indicator (Thermo Fisher, Dreieich, Germany) according to the manufacturer's protocol and then imaged and analyzed using the Operetta high content imaging system (Thermo Fisher, Dreieich, Germany). 5 μm thick Cryosections from 4 weeks old control or *Gorab* ^*Prx1*^ mice were prepared according to published protocol (Kawamoto & Shimizu, 2000). Sections were then stained with Mitosox™ stain according to manufacturer's protocol and analysed by Image J.

### 4.8 Zebrafish husbandry

Adult zebrafish and embryo were maintained under standard laboratory conditions at 28.5°C (Zebrafish Core Facility, LKS Faculty of Medicine, HKU). Zebrafish embryos were raised in E3 medium E3 medium (5 mM NaCl, 0.17 mM KCl, 0.33 mM CaCl_2_, and 0.33 mM MgSO_4_) and developmental staging was determined. For microscopic observation, 0.003% 1‐phenyl‐2‐thiourea (PTU) (Sigma, St. Louis, MO, USA) in was used to suppress pigment formation after gastrulation. All zebrafish experiments were approved by the Committee of the Use of Laboratory and Research Animals (CULATR) at the University of Hong Kong.

### 4.9 TALEN‐mediated *gorab* mutagenesis

GoldyTALEN pair targeting zebrafish *gorab* exon‐1 was designed with a PstI restriction site included in the middle of the spacer and synthesized as described in previous study (Ma et al., 2013). TALEN plasmids were linearized by SacI and purified with PCR Purification Kit (Qiagen, Hilden, Germany). TALEN encoding mRNA was generated by in vitro transcription using the mMESSAGE mMachine T3 Kit (Life Technologies, Darmstadt, Germany) and microinjected into 1‐cell stage zebrafish embryos. RFLP assay was used to evaluate the somatic mutagenic activity of the g*orab* TALEN pair as described (Ma et al., 2013).

### 4.10 Zebrafish expression analysis

Total RNA were extracted from a pool of at least 20 embryos with Trizol Reagent (Life Technologies, Darmstadt, Germany) and cDNA was subsequently synthesized with SuperScript II First‐Strand Synthesis System (Life Technologies, Darmstadt, Germany). Quantitative PCR was performed using the StepOnePlus Real‐time PCR system (Life Technologies, Darmstadt, Germany) with Power SYBR Green Master Mix (Life Technologies, Darmstadt, Germany). Expression level was presented as fold‐change calculated using the comparative C^T^ method with *βactin* as the house keeping control. Primer sequences are given in Table S1.

### 4.11 Zebrafish mitochondrial superoxide measurement, confocal microscopy, and flow cytometry

MitoSOX Red Mitochondrial Superoxide Indicator (Thermo Fisher, Dreieich, Germany) at 2.5 μM was added to PTU‐treated embryos were at 30 hpf and incubated 28.5°C for 30 min. After incubation, embryos were transferred to fresh E2 medium and anesthetized with 0.016% Tricane/MS‐222 (Sigma‐Aldrich, Munich, Germany). For confocal microscopy, embryos were mounted in 1% low melting point agarose (Sigma‐Aldrich, Munich, Germany) and imaged by Zeiss LSM510 Meta confocal microscope (Carl Zeiss, Jena, Germany) in Faculty Core Facility, LKS Faculty of Medicine, HKU. For flow cytometry analysis, at least 20 embryos were digested with 0.05% Trypsin/EDTA (Life Technologies, Darmstadt, Germany) for 15 min at 28.5°C and dissociated to single‐cell suspension by pipetting. Trypsin digestion was terminated by 10 μL of CaCl_2_ (0.1 mM), and the percentage of MitoSOX Red+ cells was evaluated by flow cytometry (Cytomics FC 500MPL, Beckman Coulter, Krefeld, Germany).

### 4.12 Colony formation unit

Femora of 12 weeks mice were collected, the epiphysis of the bone were cut off, and the bone marrow stromal cells were flushed out with a syringe and PBS. The red blood cells were then lysed using Red Blood Cell Lysis buffer (Miltenyi Biotech, Germany) according tomanufacturer's protocol. 1 × 10^6^ cells were then plated in a 75cm^2^ flask for 14 days. Cell colonies were then stained with 0.2% crystal violet solution and quantitated.

### 4.13 bmMSCs flow analysis

The femora were explanted and transferred in ice‐cold PBS. Both ends of the bone were cut using scissors and the bone marrow was flushed using a 24G needle directly onto a cell strainer. A single cell suspension was achieved using a pestle and pressing the bone marrow through the cell strainer. After washing and cell counting, 1 × 10^6^ cells per sample were stained and analyzed. The staining included a live/dead staining (LIVE/DEAD Fixable dye, ThermoFisher, Waltham, USA) in a protein free buffer (PBS) for 30 min at 4°C and blocking of unspecific binding by fc receptors (fcX Plus, BioLegend, San Diego, USA) for 10 min. The fluorescent coupled antibody staining was performed at 4°C for 20 min in a staining buffer containing 0.5% bovine serum albumin (Sigma, St. Louis, USA). For a list of antibodies, refer to Table S2. After subsequent washing step, the cells were fixated (FluoroFix, BioLegend, San Diego, USA) for 30 min at room temperature and run on a CytoFlex LX system (BeckmanCoulter, Brea, USA). Data analysis was performed with FlowJo (BD Biosciences, Franklin Lakes, USA).

## AUTHOR CONTRIBUTIONS

W.L.C designed and performed the major part of the experiments and wrote the manuscript. C.B. performed flow cytometry. J.G. performed histomorphometrical analysis. A.C.M. performed the zebrafish experiments. M.S. did the oxidative stress analysis. B.M.W. participated in the expression analysis and reviewed the manuscript. S.M. reviewed the manuscript. U.K. supervised and wrote the manuscript.

## CONFLICT OF INTEREST STATEMENT

Authors have no conflict of interest to declare.

## Supporting information


Figures S1.–S13.

Table S1.

Table S2.

## Data Availability

The data that supports the findings of this study are available in the supplementary material of this article. Further inquiries can be directed to the corresponding author.
