# Supplementary material for: Targeting TGF‐β signaling, oxidative stress, and cellular senescence rescues osteoporosis in gerodermia osteodysplastica
Source: Aging Cell. 2024 Sep 5;23(12):e14322. doi: 10.1111/acel.14322 (PMC11634742; doi:10.1111/acel.14322)
Supplement: Supplementary file 1 — Figures S1.–S13. [file ACEL-23-e14322-s002.pdf]

Supplementary Figure 1

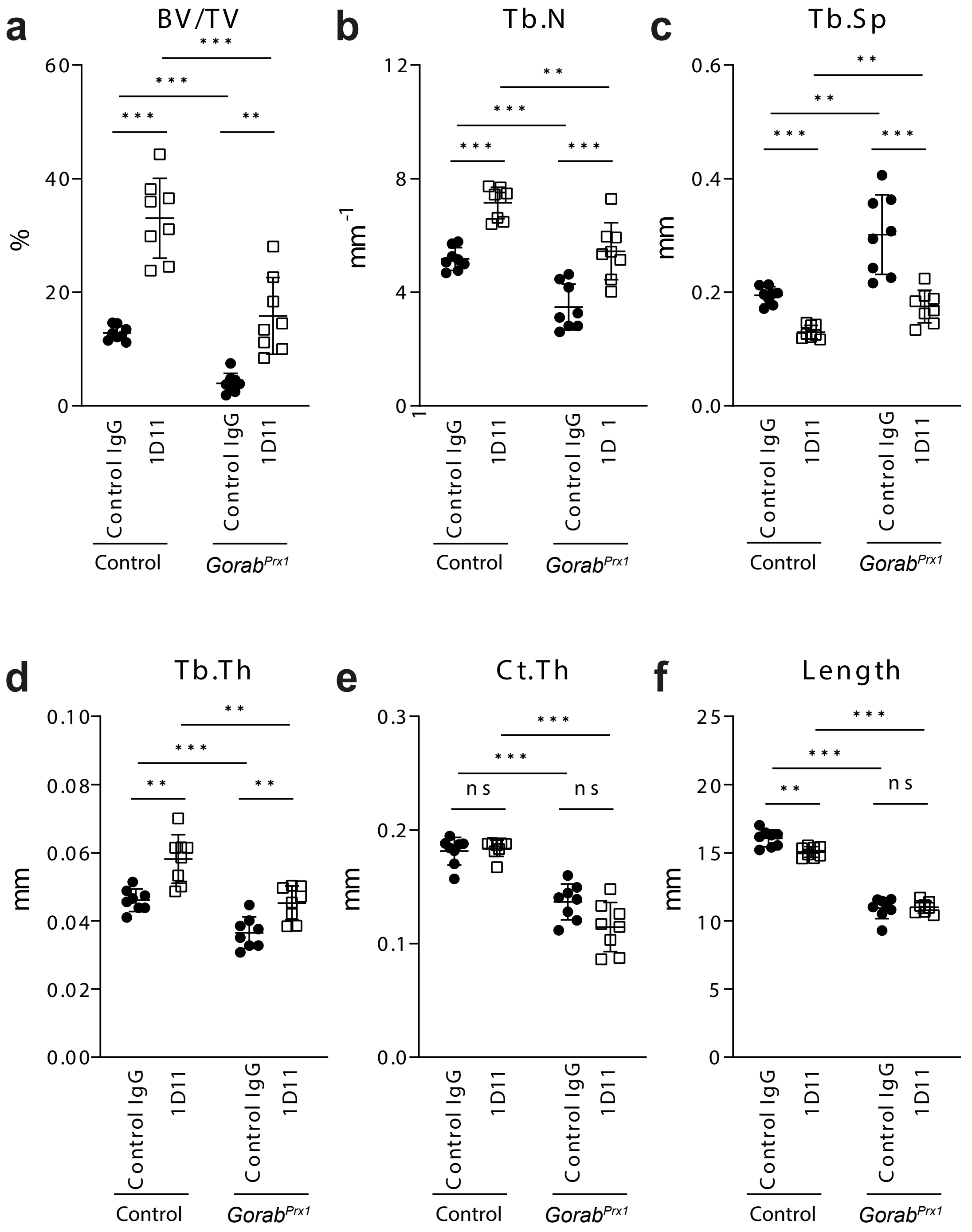

Supplementary Figure 1. TGF-β neutralizing antibody treatment rescued the trabecular bone phenotype in the femur of *Gorab^{Prx1}* mice, but not the cortical bone phenotype. microCT quantitation of (a) percentage of trabecular bone volume (BV/TV); (b) trabecular number (Tb.N); (c) trabecular separation (Tb.Sp); and (d) trabecular thickness (Tb.Th); (e) the cortical bone thickness and (f) bone length of the femurs of 12 weeks old female control and*Gorab^{Prx1}* (N=8) mice. Scanned and analyzed with Scanco µCT40 at 10µm. **p<0.05, *** p<0.001, n.s. = no statistically significant difference (unpaired Welch's t-test).

Supplementary Figure 2

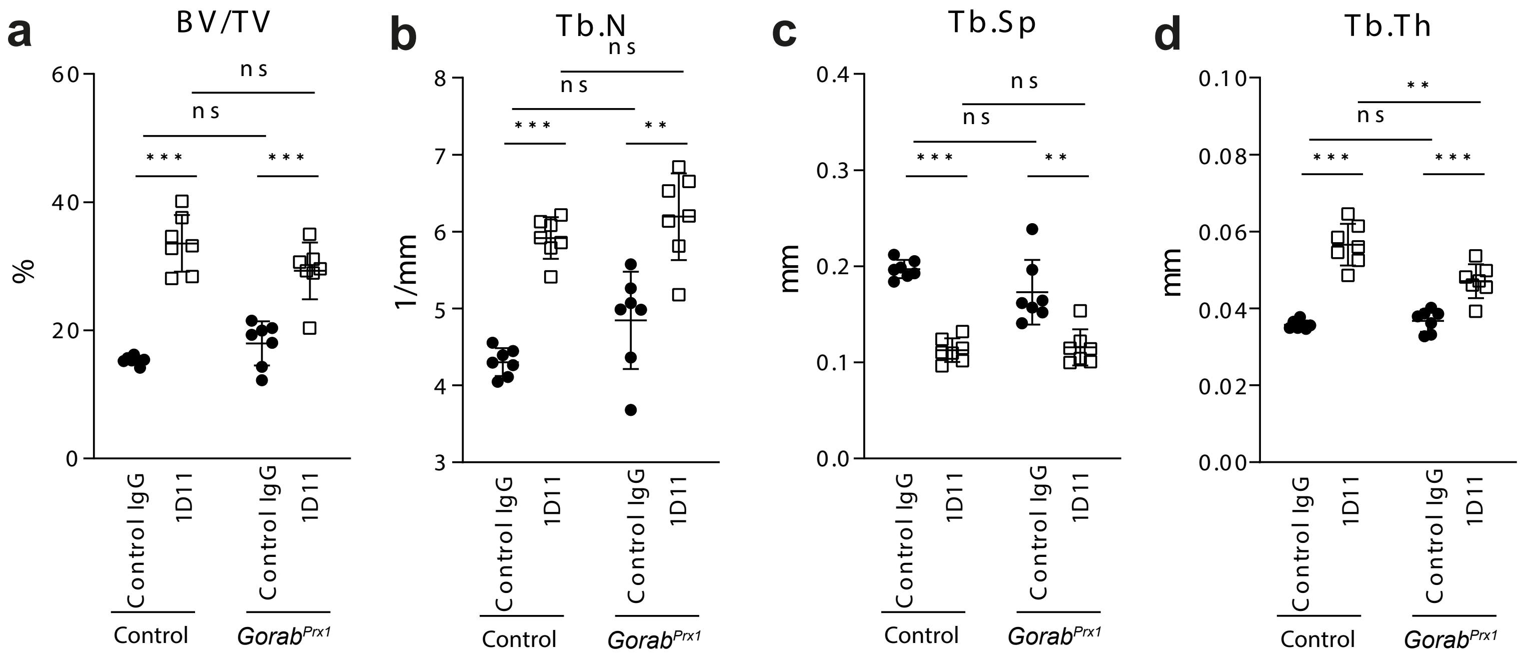

Supplementary Figure 2. TGF-β neutralizing antibody treatment significantly increased trabecular bone in the Gorab expressing vertebrae of *Gorab^{Prx1}* mice. MicroCT quantitation of (a) percentage of trabecular bone volume (BV/TV); (b) trabecular number (Tb.N); (c) trabecular separation(Tb.Sp); and (d) trabecular thickness (Tb.Th) in 6th lumbar vertebrae of 12 weeks old *Gorab^{Prx1}* mice after 1D11 treatment (N=7). Scanned and analyzed with Scanco µCT40 at 10µm. **$p<0.01$, *** $p<0.001$, n.s. = no statistically significant difference (unpaired Welch's t-test).

Supplementary Figure 3

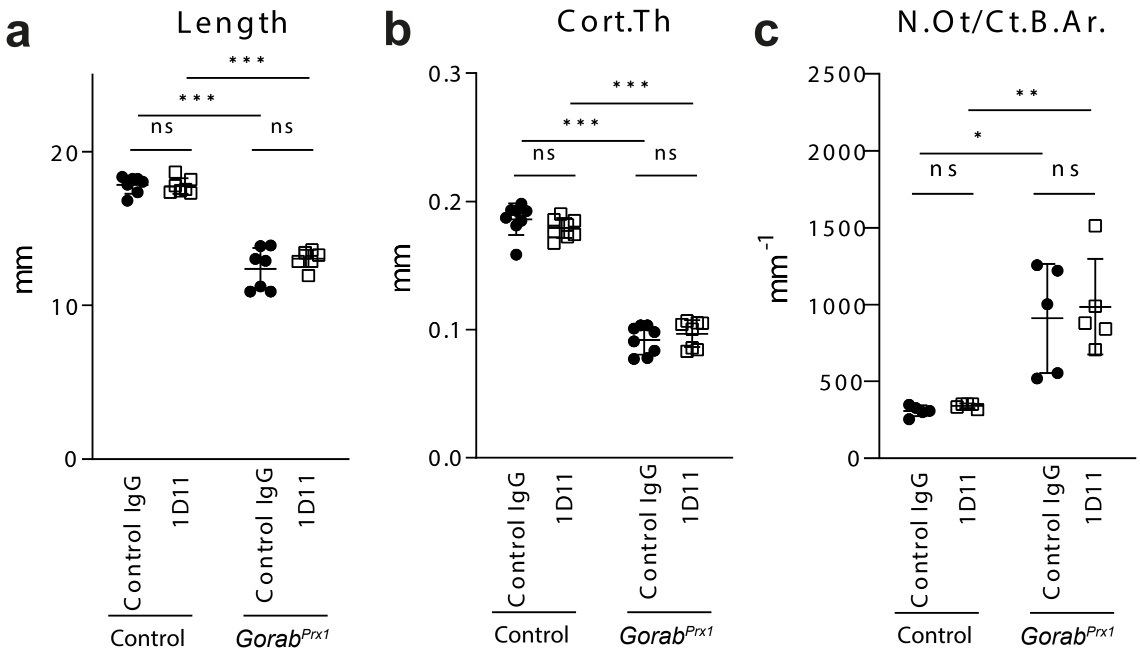

Supplementary Figure 3. TGF-β neutralizing antibody treatment has no effect on tibia length and the cortical bone phenotype. (a) Length and (b) cortical bone thickness of tibiae of *Gorab*^{Prx1} mutants showed no significant changes after 8 weeks of 1D11 treatment (N=7). Scanned and analyzed with Scanco µCT40 at 10µm. (c) Number of osteocytes per cortical bone area (N.Ot/Ct.B.Ar.) of tibiae of *Gorab*^{Prx1} mutants also showed no significant changes after 8 weeks of 1D11 treatment (N=5). *p<0.05, **p<0.01, *** p<0.001, n.s. = no statistically significant difference (unpaired Welch's t-test).

Supplementary Figure 4

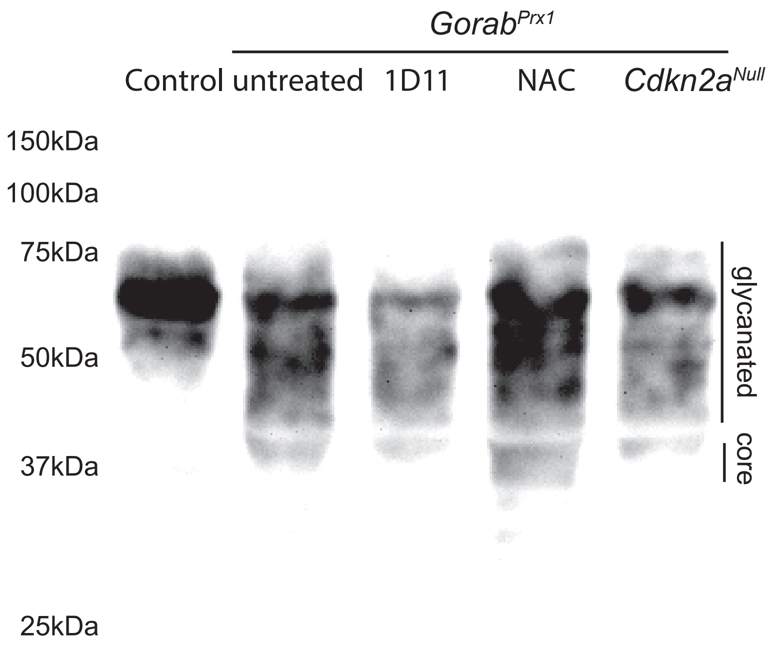

Supplementary Figure 4. 1D11, NAC and *Cdkn2a* knockout have no effect on decorin glycanation in *Gorab*^{Prx1} mice. Western blot analysis of decorin from the femur diaphysis of *Gorab*^{Prx1} mice after treatments showed no changes in protein glycanation, indicating that the TGF-β-induced senescence pathway was downstream of protein glycanation defect and ECM disruption in GO.

Supplementary Figure 5

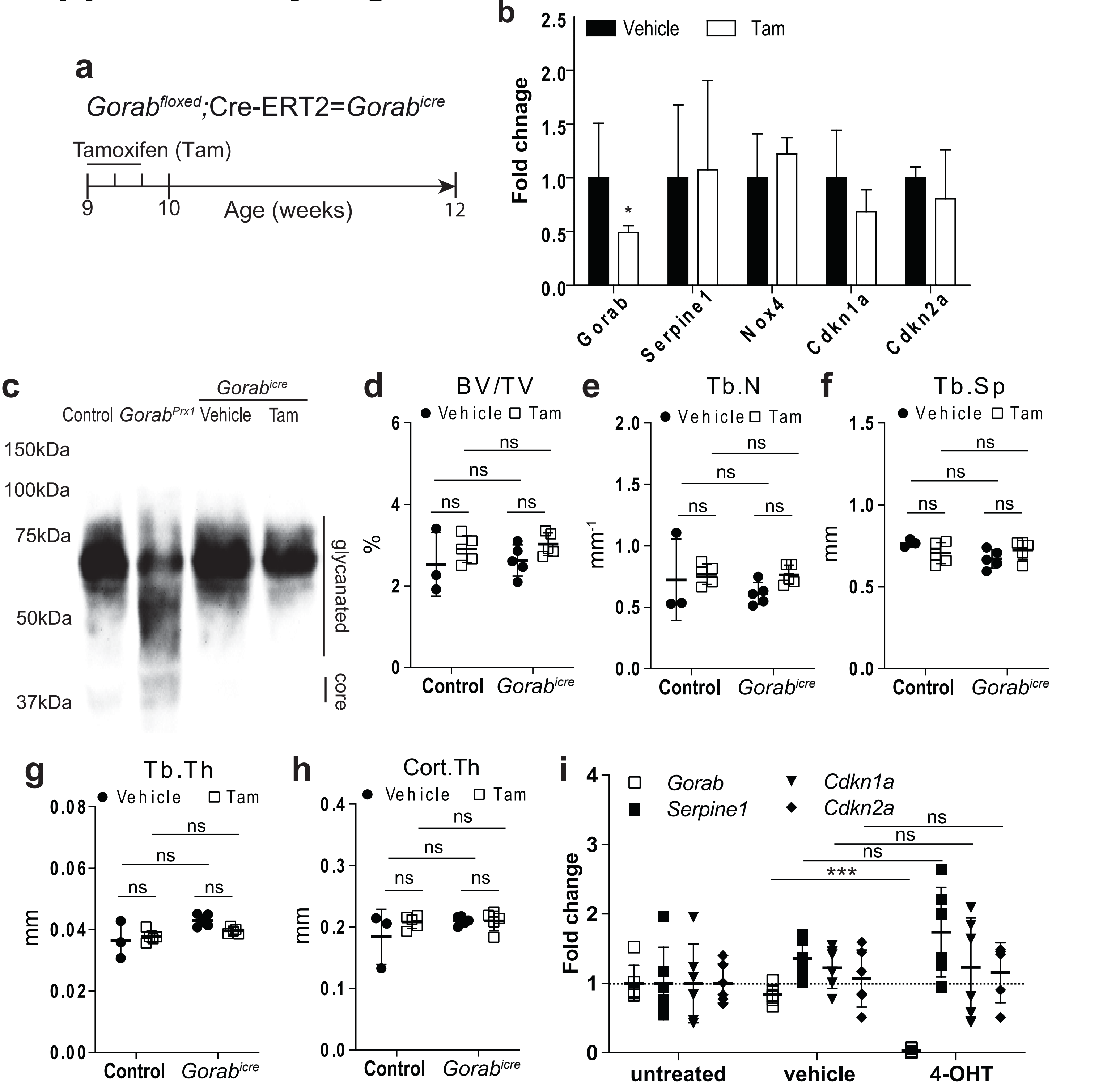

Supplementary Figue 5. Bone loss and overactivation of TGF-β signaling is not due to cell-intrinsic effects of Gorab-deficiency. (a). *Gorab*^{icre} mice were injected three times with vehicle or Tamoxifen(Tam) at 9 weeks of age and harvested at 12 weeks of age for analysis. (b) Expression of *Gorab* and TGF-β responsive genes in *Gorab*^{icre} mice two weeks after vehicle or tamoxifen treatment (N=5 vehicle treated *Gorab*^{icre}, N=6 tamoxifen treated *Gorab*^{icre}). (c) Western blot analysis showing no significant changes in decorin glycanation two weeks after tamoxifen injection in *Gorab*^{icre}*,* demonstrating the lack of immediate effect of *Gorab* knockout on the ECM *in vivo*. microCT quantitation of (d) percentage of trabecular bone volume (BV/TV); (e) trabecular number (Tb.N); (f) trabecular separation (Tb.Sp); (g) trabecular thickness (Tb.N) and (h) cortical bone thickness (Cort. Th) at the proximal tibia of Control or *Gorab*^{icre} mice 2 weeks after treatment (N=3 vehicle treated control, N=5 tamoxifen treated control, N=5 vehicle treated *Gorab*^{icre} and N=5 tamoxifen treated control). Scanned and analyzed with Bruker Skyscan 1172 at 5μm. (i) Expression of *Gorab* and TGF-β responsive genes in primary calvarial osteoblast cultures from *Gorab*^{icre} three days after vehicle or 4-hydroxytamoxifen (4-OHT) treatment (N= 6). *p<0.05, *** p<0.001, ns = no statistically significant difference (unpaired Welch's t-test).

Supplementary Figure 6

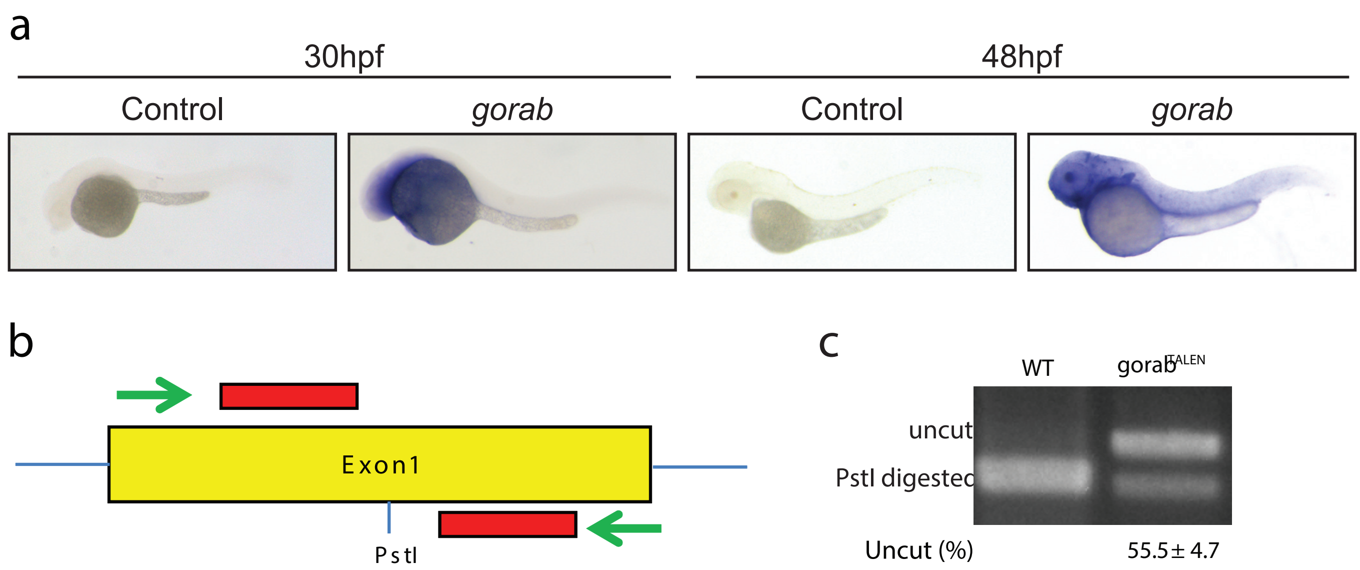

Supplementary Figure 6. Inactivation of *gorab* in zebrafish. (a) in-situ hybridization of *gorab* in the zebrafish embryos at 30 and 48hpf. Controls were done by using sense probe against *gorab*. (b) The design of Talens against the *gorab* locus in zebrafish. Green arrows represent primers for genotyping. The red bars are the targeted position of the Talens. Successful Talen mediated mutation resulted in destruction of the PstI sites and generate loss of function mutation in the *gorab* gene. (c) Genotyping showing *gorab* knock-down efficiency in Talen-injected mutant zebrafish.

Supplementary Figure 7

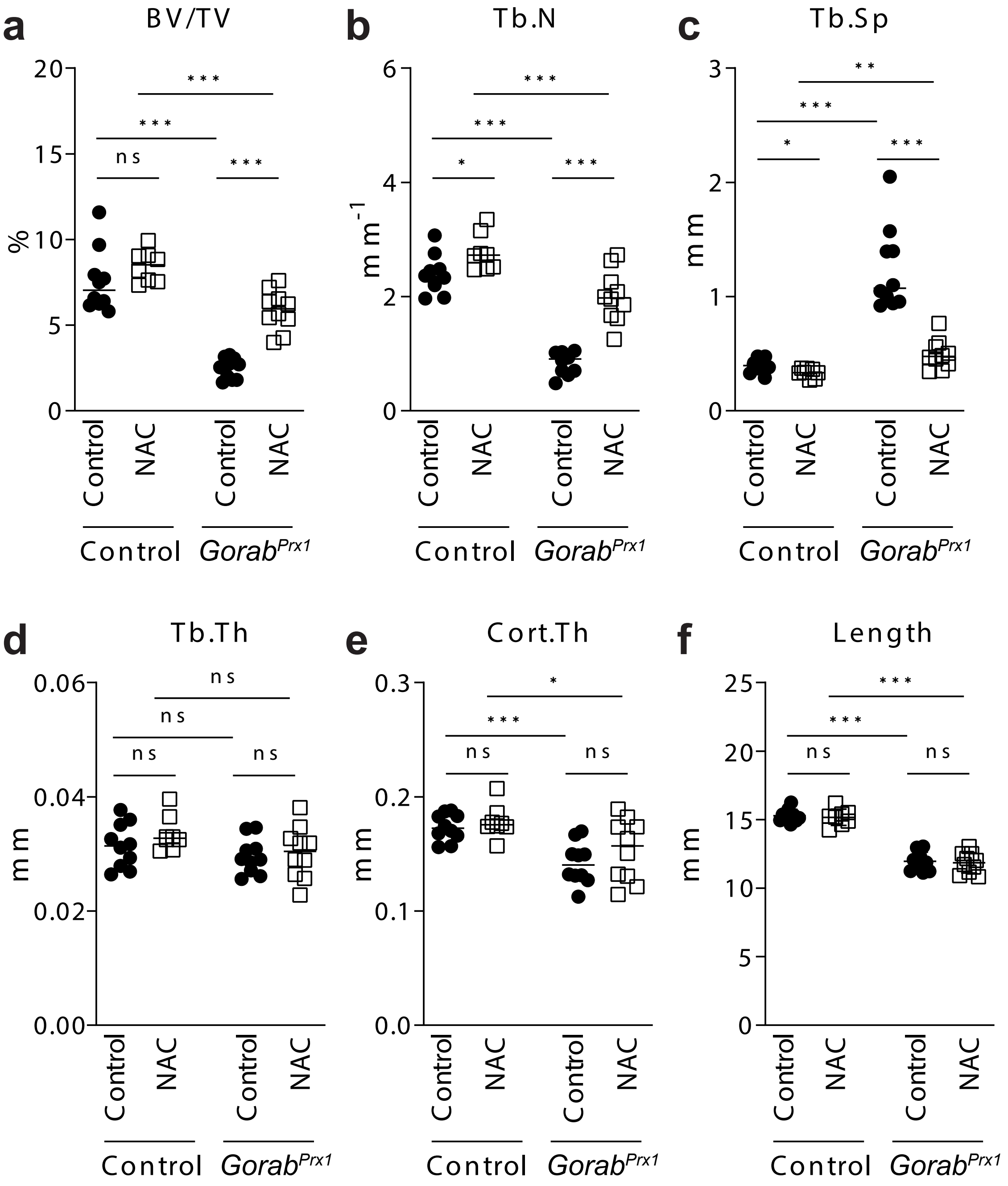

Supplementary Figure 7. Antioxidant treatment ameliorated osteoporosis in the femur of *Gorab^{Prx1}* mice. microCT quantitation of (a) percentage of trabecular bone volume (BV/TV); (b) trabecular number (Tb.N); (c) trabecular separation (Tb.Sp); (d) trabecular thickness (Tb.Th); (e) cortical thickness and (f) length of the femurs of 12 weeks old control mice and *Gorab^{Prx1}* mice treated with NAC (N=8-10). Scanned and analyzed with Scanco µCT40 at 10µm. *p<0.05, **p<0.01, *** p<0.001, n.s. = no statistically significant difference (unpaired Welch's t-test).

Supplementary Figure 8

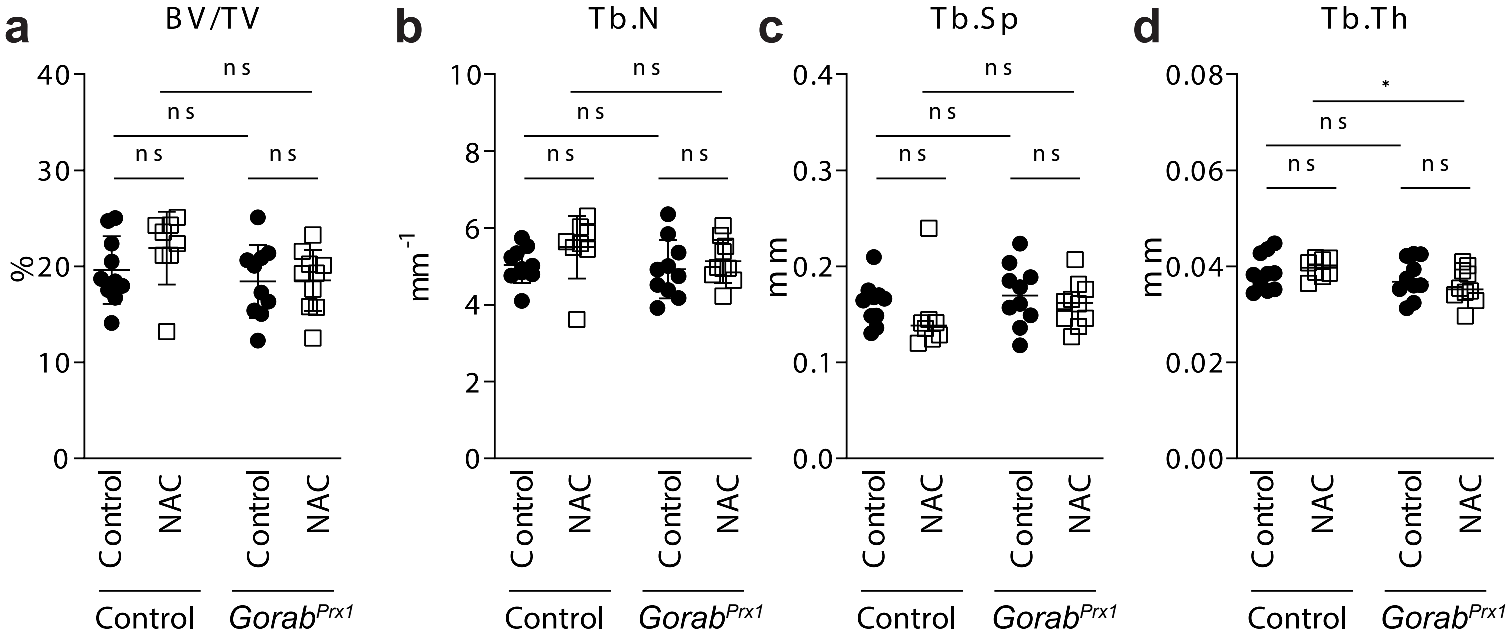

Supplementary Figure 8. Antioxidant treatment has no significant effect on bone microarchitecture in the Gorab expressing vertebrae of *Gorab^{Prx1}* mice. microCT quantitation of (a) percentage of trabecular bone volume (BV/TV); (b) trabecular number (Tb.N); (c) trabecular separation (Tb.Sp); (d) trabecular thickness (Tb.Th) of 6th lumbar vertebrae of 12 weeks old control mouse and *Gorab^{Prx1}* treated with NAC (N=8-10). Scanned and analyzed with Scanco μCT40 at 10μm. * $p<0.05$, n.s. = no statistically significant difference (unpaired Welch's t-test).

Supplementary Figure 9

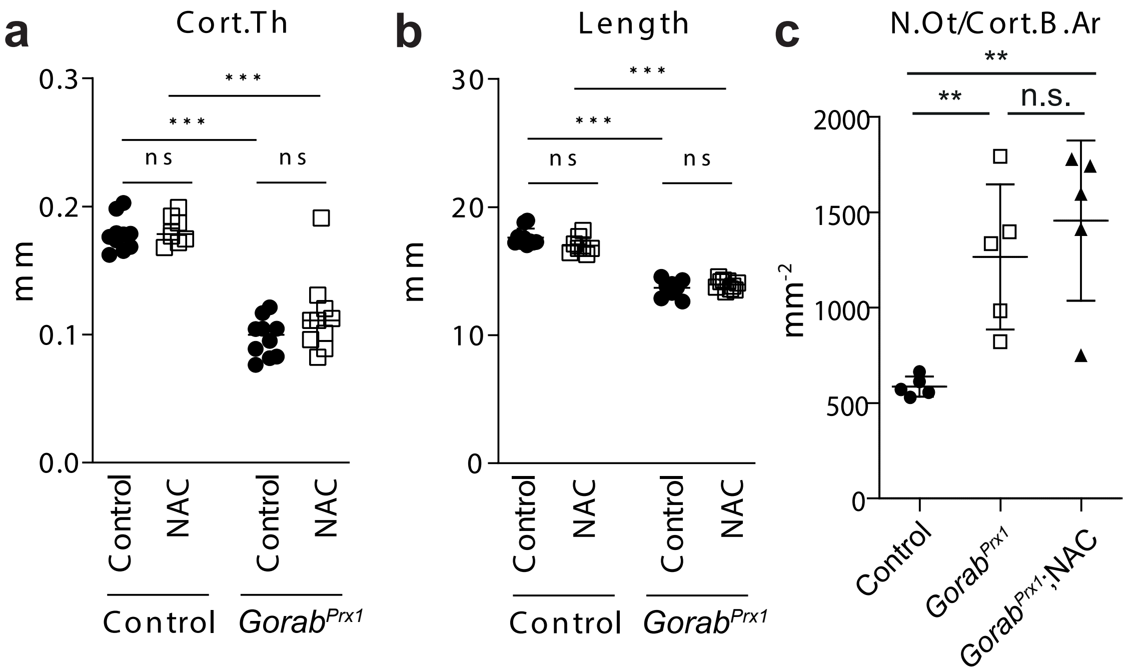

Supplementary Figure 9. Antioxidant treatment has no effect on cortical bone phenotype in the tibiae of *Gorab*^{Prx1} mice. (a) cortical bone thickness (Cort. Th) and (b) the length of tibia of 12 weeks old control mouse and *Gorab*^{Prx1} treated with NAC (N=8-10). Scanned and analyzed with Scanco µCT40 at 10µm. (c) Number of osteocytes per cortical bone area (N.Ot/Ct.B.Ar.) of tibiae of 12 weeks old control mice and *Gorab*^{Prx1} mice treated with NAC (N=5). **$p<0.01$, *** $p<0.001$, n.s. = no statistically significant difference (unpaired Welch's t-test).

Supplementary Figure 10

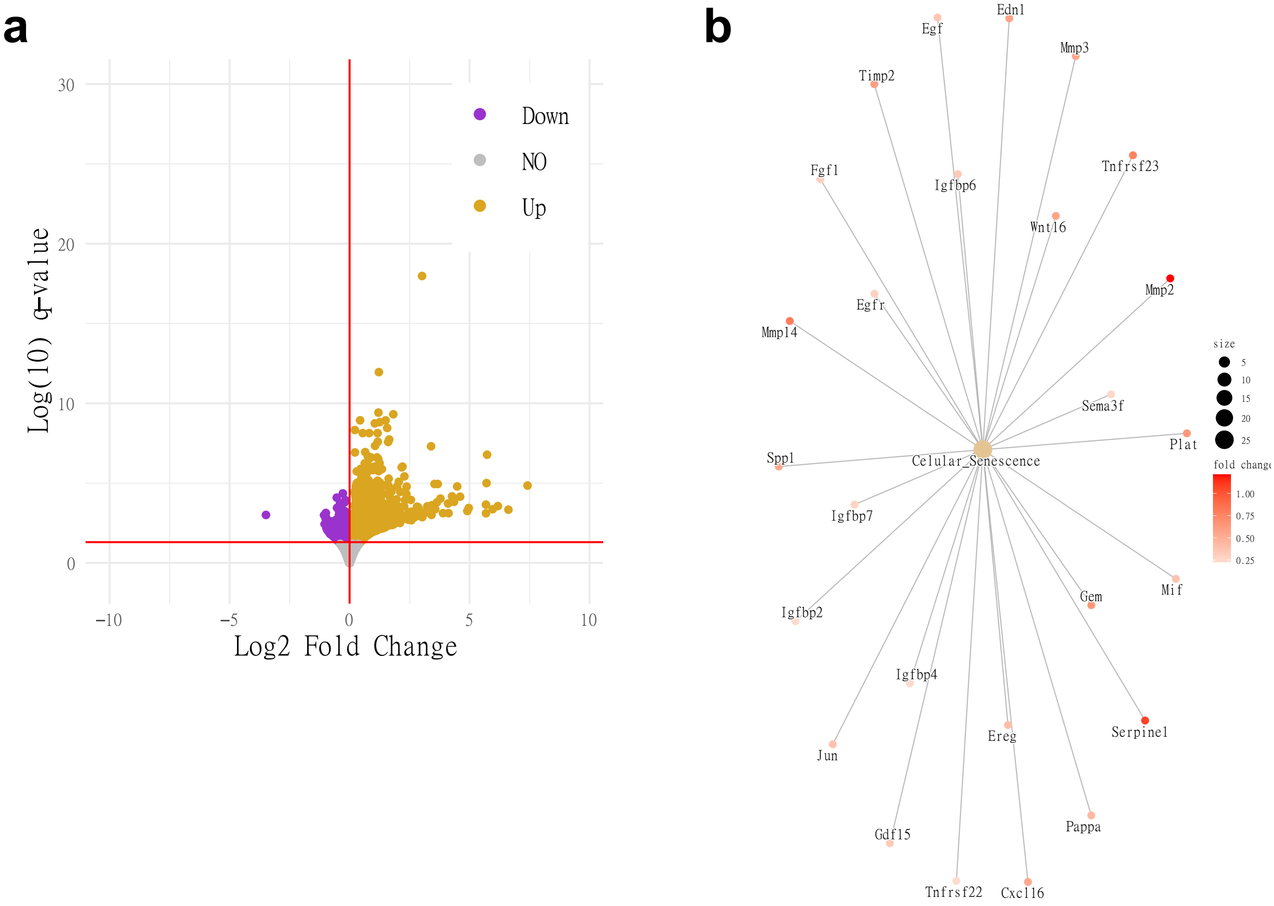

Supplementary Figure 10. Cellular senescence signature in upregulated genes in transcriptome profiling. Transcriptome profiling of bone tissue from 12 week-old *Gorab*^{Prx1} mutants (N=9) and controls (N=6). (a) Volcano plot showing predominant upregulation of genes. (b) Upregulated genes known to be associated with cellular senescence.

Supplementary Figure 11

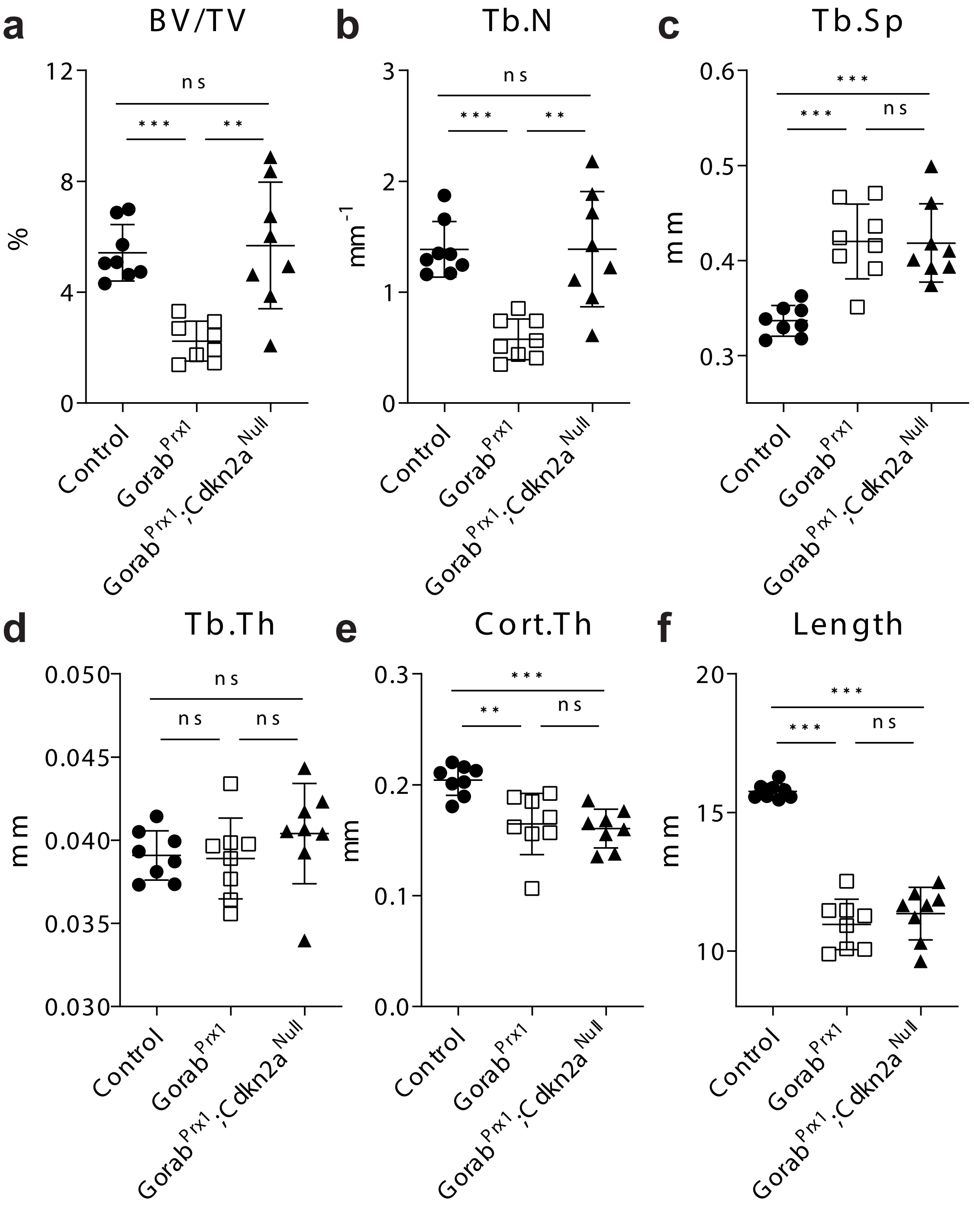

Supplementary Figure 11. Inactivation of *Cdkn2a* rescues the femoral trabecular bone phenotype of *Gorab^{Prx1};Cdkn2a^{Null}* mice, but not the cortical bone phenotype. microCT quantitation of (a) percentage of trabecular bone volume (BV/TV); (b) trabecular number (Tb.N); (c) trabecular separation (Tb.Sp); and (d) trabecular thickness (Tb.N); (e) the cortical bone thickness and (f) bone length of the femurs of female control, *Gorab^{Prx1}* and *Gorab^{Prx1};Cdkn2a^{Null}* mice at 12 weeks of age (N=8). Scanned and analyzed with Bruker Skyscan 1172 at 5µm. **p<0.01, *** p<0.001, n.s. = no statistically significant difference (unpaired Welch's t-test).

Supplementary Figure 12

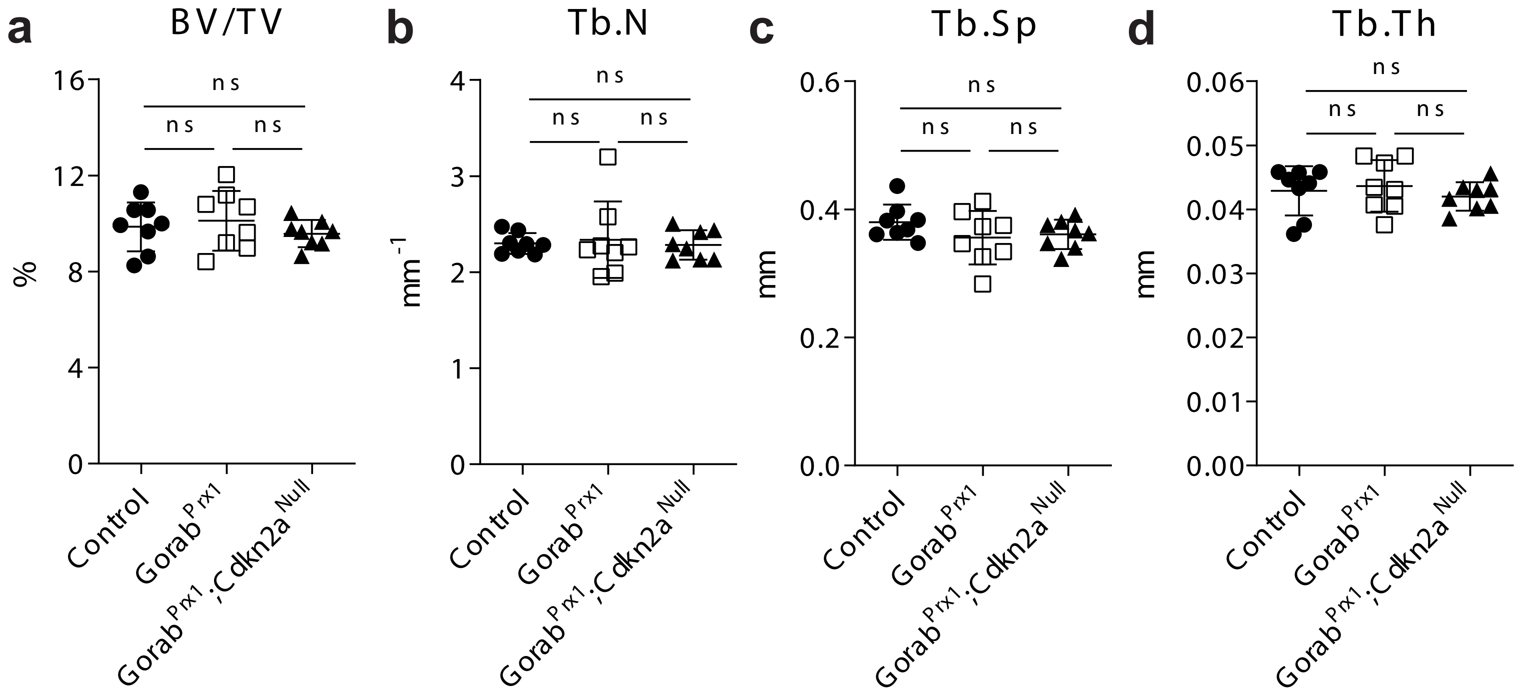

Supplementary Figure 12. Inactivation of *Cdkn2a* has no effect on bone microarchitecture in the Gorab expressing vertebrae of *Gorab^{Prx1};Cdkn2a^{Null}* mice. microCT quantitation of (a) percentage of trabecular bone volume (BV/TV); (b) trabecular number (Tb.N); (c) trabecular separation (Tb.Sp); and (d) trabecular thickness (Tb.Th) of the 6th lumbar vertebrae of female control, *Gorab^{Prx1}* and *Gorab^{Prx1};C-dkn2a^{Null}* mice at 12 weeks of age (N=8). Scanned and analyzed with Bruker Skyscan 1172 at 5µm. ns = no statistically significant difference (unpaired Welch's t-test).

Supplementary Figure 13

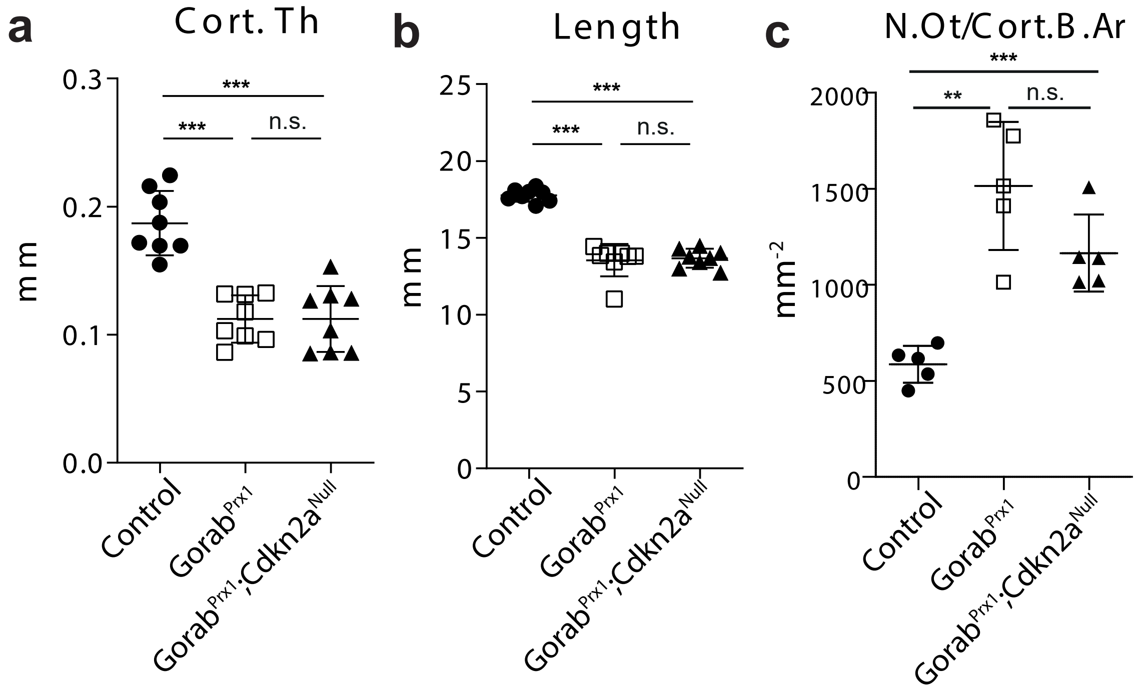

Supplementary Figure 13. Inactivation of Cdkn2a has no rescue effect on cortical bone phenotype of *Gorab^{Prx1};Cdkn2a^{Null}* mice. (a) the cortical bone thickness and (b) bone length of female control, *Gorab^{Prx1}* and *Gorab^{Prx1};Cdkn2a^{Null}* mice at 12 weeks of age (N=8). Scanned and analyzed with Bruker Skyscan 1172 at 5µm. (c) Number of osteocytes per cortical bone area in the tibia of 12 weeks old *Gorab^{Prx1};Cdkn2a^{Null}* mice (N=5). **p<0.01, *** p<0.001, n.s. = no statistically significant difference (unpaired Welch's t-test).
