# Supplementary material for: Targeting TGF‐β signaling, oxidative stress, and cellular senescence rescues osteoporosis in gerodermia osteodysplastica
Source: Aging Cell. 2024 Sep 5;23(12):e14322. doi: 10.1111/acel.14322 (PMC11634742; doi:10.1111/acel.14322)
Supplement: Supplementary file 2 — Table S1. [file ACEL-23-e14322-s001.pdf]

Supplementary Table 1. Transcriptome profiling *Gorab* ^{Prx1} vs. control bone tissue, 12 weeks of age

Gene	baseMean	log2FoldChange	lfcSE	pvalue	padj	Symbol	Locus	Description	Gene_type
ENSMUSG00000049133	136.0241148	33.31958519	4.189649081	2.39E-16	1.12E-12	Flg2	3:93197278-93221391	filaggrin family member 2 [Source:MGI Symbol;Acc:MGI:3645678]	protein_coding
ENSMUSG00000041991	99.10496054	31.57449854	4.186718233	7.65E-15	2.09E-11	Hrnr	3:93319749-93333570	hornerin [Source:MGI Symbol;Acc:MGI:3046938]	protein_coding
ENSMUSG00000067594	112.5302599	24.52050416	3.852561911	6.85E-11	5.16E-08	Krt77	15:101858731-101869705	keratin 77 [Source:MGI Symbol;Acc:MGI:3588209]	protein_coding
ENSMUSG00000045545	189.9553926	10.37197545	2.13787051	2.20E-08	7.29E-06	Krt14	11:100203162-100207548	keratin 14 [Source:MGI Symbol;Acc:MGI:96688]	protein_coding
ENSMUSG00000060177	72.21166013	7.415004948	1.920564781	5.67E-08	1.43E-05	Klk1b22	7:44112673-44116922	kallikrein 1-related peptidase b22 [Source:MGI Symbol;Acc:MGI:95291]	protein_coding
ENSMUSG00000034607	64.38708167	6.179081929	3.001523043	2.99E-06	2.76E-04	Pof1b	X:112638431-112698651	premature ovarian failure 1B [Source:MGI Symbol;Acc:MGI:1916943]	protein_coding
ENSMUSG00000061527	179.9764437	5.965269629	3.502506688	5.75E-06	4.30E-04	Krt5	15:101707070-101712898	keratin 5 [Source:MGI Symbol;Acc:MGI:96702]	protein_coding
ENSMUSG00000069441	83.1499568	5.683517865	4.341225936	1.27E-05	7.75E-04	Dsg1a	18:20310811-20343350	desmoglein 1 alpha [Source:MGI Symbol;Acc:MGI:94930]	protein_coding
ENSMUSG00000059898	68.92276252	5.682221981	2.507043632	2.22E-06	2.24E-04	Dsc3	18:19960930-20002351	desmocollin 3 [Source:MGI Symbol;Acc:MGI:1194993]	protein_coding
ENSMUSG00000030713	54.77707964	4.905975964	3.04765371	7.85E-06	5.61E-04	Klk7	7:43811294-43816359	kallikrein related-peptidase 7 (chymotryptic, stratum corneum) [Source:MGI Symbol;Acc:MGI:134633	protein_coding
ENSMUSG00000053522	135.6057499	4.269081051	1.193905159	4.72E-07	6.80E-05	Lgals7	7:28863853-28866284	lectin, galactose binding, soluble 7 [Source:MGI Symbol;Acc:MGI:1316742]	protein_coding
ENSMUSG00000046834	172.137021	4.123166379	2.776302286	1.23E-05	7.67E-04	Krt1	15:101845426-101850794	keratin 1 [Source:MGI Symbol;Acc:MGI:96698]	protein_coding
ENSMUSG00000033765	125.4173025	3.891468185	2.45871737	1.23E-05	7.65E-04	Calm4	13:3837757-3838673	calmodulin 4 [Source:MGI Symbol;Acc:MGI:1931464]	protein_coding
ENSMUSG00000060093	8342.525827	3.785229412	1.072676575	7.17E-07	9.39E-05	H4c1	13:23760692-23761230	H4 clustered histone 1 [Source:MGI Symbol;Acc:MGI:2448419]	protein_coding
ENSMUSG00000019761	699.4699202	3.67402684	0.798647258	4.02E-08	1.14E-05	Krt10	11:99385254-99389364	keratin 10 [Source:MGI Symbol;Acc:MGI:96685]	protein_coding
ENSMUSG00000100210	17920.31865	3.568090483	1.497696614	5.68E-06	4.30E-04	H3c7	13:23544052-23545312	H3 clustered histone 7 [Source:MGI Symbol;Acc:MGI:2448329]	protein_coding
ENSMUSG00000066364	60.13179489	3.537603341	0.770654231	4.11E-08	1.14E-05	Serpina3b	12:104127996-104139545	serine (or cysteine) peptidase inhibitor, clade A, member 3B [Source:MGI Symbol;Acc:MGI:2182835]	protein_coding
ENSMUSG00000040703	298.5299157	3.433809859	0.245644473	1.85E-45	4.04E-41	Cyp2s1	7:25802475-25816913	cytochrome P450, family 2, subfamily s, polypeptide 1 [Source:MGI Symbol;Acc:MGI:1921384]	protein_coding
ENSMUSG00000064288	18591.80658	3.425234288	1.162362402	2.53E-06	2.45E-04	H4c12	13:21750194-21750505	H4 clustered histone 12 [Source:MGI Symbol;Acc:MGI:2448439]	protein_coding
ENSMUSG00000074199	133.6598577	3.413321066	2.196968556	1.75E-05	9.90E-04	Krtdap	7:30787896-30791097	keratinocyte differentiation associated protein [Source:MGI Symbol;Acc:MGI:1928282]	protein_coding
ENSMUSG00000058914	395.0820969	3.394433524	0.567684801	6.34E-11	4.95E-08	C1qtnf3	15:10952332-10980150	C1q and tumor necrosis factor related protein 3 [Source:MGI Symbol;Acc:MGI:1932136]	protein_coding
ENSMUSG00000032013	54.28818471	3.238949168	1.131215245	3.15E-06	2.85E-04	Trim29	9:43310848-43336115	tripartite motif-containing 29 [Source:MGI Symbol;Acc:MGI:1919419]	protein_coding
ENSMUSG00000069273	13816.28877	3.115341186	1.52771792	1.26E-05	7.74E-04	H3c6	13:23561534-23562369	H3 clustered histone 6 [Source:MGI Symbol;Acc:MGI:2448326]	protein_coding
ENSMUSG00000028262	53.59822741	3.030504371	2.142059411	2.73E-05	0.001367527	Clca3a2	3:144796559-144819494	chloride channel accessory 3A2 [Source:MGI Symbol;Acc:MGI:1931471]	protein_coding
ENSMUSG00000067455	10723.59908	2.920248935	1.273170765	9.61E-06	6.45E-04	H4c11	13:21735064-21735837	H4 clustered histone 11 [Source:MGI Symbol;Acc:MGI:2448436]	protein_coding
ENSMUSG00000061482	29902.22503	2.895987861	1.110260566	5.56E-06	4.27E-04	H4c4	13:23581598-23582735	H4 clustered histone 4 [Source:MGI Symbol;Acc:MGI:2448423]	protein_coding
ENSMUSG00000069274	11500.91713	2.845870309	1.57243433	2.10E-05	0.001120883	H4c6	13:23551258-23551648	H4 clustered histone 6 [Source:MGI Symbol;Acc:MGI:2448425]	protein_coding
ENSMUSG00000060981	19371.41768	2.844934001	1.00528489	3.63E-06	3.12E-04	H4c8	13:23531050-23531519	H4 clustered histone 8 [Source:MGI Symbol;Acc:MGI:2448427]	protein_coding
ENSMUSG00000095217	8529.096459	2.808051468	1.22006861	9.99E-06	6.67E-04	H2bc15	13:21754123-21754503	H2B clustered histone 15 [Source:MGI Symbol;Acc:MGI:2448407]	protein_coding
ENSMUSG00000105827	14711.69826	2.78308851	1.272195509	1.23E-05	7.65E-04	H2bc18	3:96269752-96270132	H2B clustered histone 18 [Source:MGI Symbol;Acc:MGI:2448413]	protein_coding
ENSMUSG00000069266	10761.78086	2.658046066	1.400753702	2.11E-05	0.00112413	H4c2	13:23757013-23757409	H4 clustered histone 2 [Source:MGI Symbol;Acc:MGI:2448420]	protein_coding
ENSMUSG00000101972	7421.88129	2.650150957	1.212137588	1.31E-05	7.92E-04	H3c11	13:21782960-21783370	H3 clustered histone 11 [Source:MGI Symbol;Acc:MGI:2448350]	protein_coding
ENSMUSG00000074403	7151.489809	2.629412341	1.306156393	1.79E-05	9.94E-04	H3c13	3:96268654-96269141	H3 clustered histone 13 [Source:MGI Symbol;Acc:MGI:2448351]	protein_coding
ENSMUSG00000051627	32670.64287	2.584383701	1.092119065	9.61E-06	6.45E-04	H1f4	13:23620629-23622558	H1.4 linker histone, cluster member [Source:MGI Symbol;Acc:MGI:1931527]	protein_coding
ENSMUSG00000069310	11322.896	2.577982125	1.078079751	9.17E-06	6.25E-04	H3c3	13:23744973-23745602	H3 clustered histone 3 [Source:MGI Symbol;Acc:MGI:2448320]	protein_coding
ENSMUSG00000069305	6981.788331	2.565457878	1.637920324	3.61E-05	0.001678784	Hist1h4n	13:21831767-21832196	histone cluster 1, H4n [Source:MGI Symbol;Acc:MGI:4843992]	protein_coding
ENSMUSG00000049539	21795.71285	2.529709098	1.300260612	2.12E-05	0.00112413	H1f1	13:23763666-23764406	H1.1 linker histone, cluster member [Source:MGI Symbol;Acc:MGI:1931523]	protein_coding
ENSMUSG00000091405	19013.94735	2.476895492	1.04935725	1.00E-05	6.67E-04	H4c14	3:96261682-96263311	H4 clustered histone 14 [Source:MGI Symbol;Acc:MGI:2140113]	protein_coding
ENSMUSG00000061615	7101.785448	2.398746976	1.133259258	1.66E-05	9.62E-04	H2ac4	13:23751126-23751598	H2A clustered histone 4 [Source:MGI Symbol;Acc:MGI:2448306]	protein_coding
ENSMUSG00000046167	53.24780713	2.307109377	0.696414878	9.41E-07	1.18E-04	Gldn	9:54286486-54341786	gliomedin [Source:MGI Symbol;Acc:MGI:2388361]	protein_coding
ENSMUSG00000063021	11470.08442	2.284649269	1.257895964	3.07E-05	0.001491267	H2ac15	13:21753435-21753827	H2A clustered histone 15 [Source:MGI Symbol;Acc:MGI:2448297]	protein_coding
ENSMUSG00000061397	51.50826794	2.262559875	1.249712374	3.15E-05	0.001505333	Krt79	15:101929332-101940324	keratin 79 [Source:MGI Symbol;Acc:MGI:2385030]	protein_coding
ENSMUSG00000026413	100.5787637	2.197245831	1.036677791	1.81E-05	0.001004396	Pkp1	1:135871395-135919207	plakophilin 1 [Source:MGI Symbol;Acc:MGI:1328359]	protein_coding
ENSMUSG00000062727	4944.033179	2.182788486	1.329344964	4.44E-05	0.001957505	H2bc12	13:22035870-22036345	H2B clustered histone 12 [Source:MGI Symbol;Acc:MGI:2448399]	protein_coding
ENSMUSG00000043165	254.5353246	2.124689535	1.229239404	4.00E-05	0.001797596	Lor	3:92080271-92083142	loricrin [Source:MGI Symbol;Acc:MGI:96816]	protein_coding
ENSMUSG00000024846	50.8600838	2.08916418	0.88689606	1.14E-05	7.31E-04	Cst6	19:5344705-5349574	cystatin E/M [Source:MGI Symbol;Acc:MGI:1920970]	protein_coding
ENSMUSG00000052316	824.1037503	2.050070966	0.483891492	2.57E-08	8.27E-06	Lrrc15	16:30269302-30283256	leucine rich repeat containing 15 [Source:MGI Symbol;Acc:MGI:1921738]	protein_coding
ENSMUSG00000060678	15040.53658	2.047748515	1.050767065	2.77E-05	0.00138306	H4c3	13:23697635-23698454	H4 clustered histone 3 [Source:MGI Symbol;Acc:MGI:2448421]	protein_coding
ENSMUSG00000020908	77.26530152	2.025797399	1.11676332	3.66E-05	0.001687128	Myh3	11:67078300-67102291	myosin, heavy polypeptide 3, skeletal muscle, embryonic [Source:MGI Symbol;Acc:MGI:1339709]	protein_coding
ENSMUSG00000114279	7114.888385	2.013416587	1.314861318	6.11E-05	0.002423155	H2bc14	13:21722044-21722567	H2B clustered histone 14 [Source:MGI Symbol;Acc:MGI:2448404]	protein_coding
ENSMUSG00000025064	73.40929789	1.994976181	1.080748262	3.51E-05	0.001652606	Col17a1	19:47646344-47692094	collagen, type XVII, alpha 1 [Source:MGI Symbol;Acc:MGI:88450]	protein_coding
ENSMUSG00000069300	5349.334398	1.990362218	1.153179858	4.43E-05	0.001957505	H2bc11	13:22043214-22043676	H2B clustered histone 11 [Source:MGI Symbol;Acc:MGI:2448388]	protein_coding
ENSMUSG00000075031	15501.28747	1.976531501	1.290357063	6.31E-05	0.002466861	H2bc3	13:23746734-23747241	H2B clustered histone 3 [Source:MGI Symbol;Acc:MGI:2448377]	protein_coding
ENSMUSG00000032262	91.62506262	1.948643919	0.871359662	1.61E-05	9.36E-04	Elovl4	9:83778692-83806277	elongation of very long chain fatty acids (FEN1/Elo2, SUR4/Elo3, yeast)-like 4 [Source:MGI Symbol;Acc	protein_coding
ENSMUSG00000069267	5420.299796	1.93649257	1.309577235	7.13E-05	0.002697167	H3c2	13:23752267-23752886	H3 clustered histone 2 [Source:MGI Symbol;Acc:MGI:2448319]	protein_coding
ENSMUSG00000069265	4712.870167	1.905813448	1.3966254	8.74E-05	0.003103105	H3c1	13:23761853-23762386	H3 clustered histone 1 [Source:MGI Symbol;Acc:MGI:2668828]	protein_coding
ENSMUSG00000026442	125.4067564	1.836853855	0.626296973	3.00E-06	2.76E-04	Nfasc	1:132564690-132741797	neurofascin [Source:MGI Symbol;Acc:MGI:104753]	protein_coding
ENSMUSG00000034892	9726.193184	1.822385867	0.77158804	1.32E-05	7.95E-04	Rps29	12:69157722-69159186	ribosomal protein S29 [Source:MGI Symbol;Acc:MGI:107681]	protein_coding
ENSMUSG00000004892	127.2496515	1.821599332	0.288731962	2.90E-13	4.89E-10	Bcan	3:87987531-88000230	brevican [Source:MGI Symbol;Acc:MGI:1096385]	protein_coding
ENSMUSG00000058773	22356.00951	1.818071761	1.169681723	7.00E-05	0.002662268	H1f5	13:21779883-21780625	H1.5 linker histone, cluster member [Source:MGI Symbol;Acc:MGI:1861461]	protein_coding
ENSMUSG00000068855	7536.204134	1.742892816	1.221333395	9.38E-05	0.003282262	H2ac20	3:96220361-96220880	H2A clustered histone 20 [Source:MGI Symbol;Acc:MGI:2448316]	protein_coding
ENSMUSG00000009185	168.2318079	1.742575995	0.460991611	1.91E-07	3.39E-05	Ccl8	11:82115185-82116799	chemokine (C-C motif) ligand 8 [Source:MGI Symbol;Acc:MGI:101878]	protein_coding
ENSMUSG00000026725	3400.997235	1.718439851	0.442354005	1.30E-07	2.52E-05	Tnn	1:160085029-160153580	tenascin N [Source:MGI Symbol;Acc:MGI:2665790]	protein_coding
ENSMUSG00000017404	10815.22487	1.706734062	0.713802289	1.38E-05	8.20E-04	Rpl19	11:98026710-98030492	ribosomal protein L19 [Source:MGI Symbol;Acc:MGI:98020]	protein_coding
ENSMUSG00000057666	18242.60726	1.701723118	0.625786935	6.18E-06	4.59E-04	Gapdh	6:125161715-125166467	glyceraldehyde-3-phosphate dehydrogenase [Source:MGI Symbol;Acc:MGI:95640]	protein_coding
ENSMUSG00000057751	370.8644157	1.679771798	0.427937929	1.18E-07	2.33E-05	Megf6	4:154170730-154275713	multiple EGF-like-domains 6 [Source:MGI Symbol;Acc:MGI:1919351]	protein_coding
ENSMUSG00000022887	121.4220264	1.678179967	0.477251885	5.59E-07	7.67E-05	Masp1	16:23449417-23520815	mannan-binding lectin serine peptidase 1 [Source:MGI Symbol;Acc:MGI:88492]	protein_coding
ENSMUSG00000094338	6773.983784	1.674140694	1.398428798	1.42E-04	0.004332166	H2bc13	13:21715763-21716143	H2B clustered histone 13 [Source:MGI Symbol;Acc:MGI:2448403]	protein_coding
ENSMUSG00000058385	10159.22731	1.664908829	1.094645715	8.68E-05	0.003097708	H2bc8	13:23571396-23572013	H2B clustered histone 8 [Source:MGI Symbol;Acc:MGI:2448386]	protein_coding
ENSMUSG00000071984	2628.425253	1.650996245	0.291095782	2.04E-11	1.86E-08	Fndc1	17:7738569-7827302	fibronectin type III domain containing 1 [Source:MGI Symbol;Acc:MGI:1915905]	protein_coding
ENSMUSG00000031250	301.1651115	1.634184538	0.516409944	1.93E-06	2.00E-04	Tnmd	X:133850980-133865577	tenomodulin [Source:MGI Symbol;Acc:MGI:1929885]	protein_coding
ENSMUSG00000023885	5248.59663	1.629556826	0.36782378	1.47E-08	5.26E-06	Thbs2	17:14665500-14694235	thrombospondin 2 [Source:MGI Symbol;Acc:MGI:98738]	protein_coding
ENSMUSG00000021260	244.882963	1.621128183	0.393279458	5.89E-08	1.46E-05	Hhipl1	12:108306270-108330869	hedgehog interacting protein-like 1 [Source:MGI Symbol;Acc:MGI:1919265]	protein_coding
ENSMUSG00000054196	1504.037797	1.605804991	0.285784384	3.16E-11	2.66E-08	Cthrc1	15:39076932-39087121	collagen triple helix repeat containing 1 [Source:MGI Symbol;Acc:MGI:1915838]	protein_coding
ENSMUSG00000020256	1119.909767	1.566104848	0.259709807	3.06E-12	3.52E-09	Aldh1l2	10:83487450-83534140	aldehyde dehydrogenase 1 family, member L2 [Source:MGI Symbol;Acc:MGI:2444680]	protein_coding
ENSMUSG00000028487	192.6964512	1.530929146	0.653154701	1.98E-05	0.001078691	Bnc2	4:84275095-84675275	basonuclin 2 [Source:MGI Symbol;Acc:MGI:2443805]	protein_coding
ENSMUSG00000101355	4370.346139	1.517862471	1.413858614	2.01E-04	0.005485546	H3c10	13:21717659-21718069	H3 clustered histone 10 [Source:MGI Symbol;Acc:MGI:2448349]	protein_coding
ENSMUSG00000055561	67.63087519	1.503496273	2.909327176	1.97E-04	0.005435436	Spink5	18:43963235-44022501	serine peptidase inhibitor, Kazal type 5 [Source:MGI Symbol;Acc:MGI:1919682]	protein_coding
ENSMUSG00000037855	76.7904453	1.50232378	0.23957889	8.62E-13	1.18E-09	Zfp365	10:67886103-67912662	zinc finger protein 365 [Source:MGI Symbol;Acc:MGI:2143676]	protein_coding
ENSMUSG00000066058	59.10628941	1.474127048	0.793966703	5.97E-05	0.002402012	Cldn19	4:119255414-119262438	claudin 19 [Source:MGI Symbol;Acc:MGI:3033992]	protein_coding
ENSMUSG00000062825	18463.66974	1.45114621	0.658353797	3.09E-05	0.001491267	Actg1	11:120345690-120348542	actin, gamma, cytoplasmic 1 [Source:MGI Symbol;Acc:MGI:87906]	protein_coding
ENSMUSG00000057322	4034.802963	1.438862576	0.752446335	5.69E-05	0.002316002	Rpl38	11:114668524-114672331	ribosomal protein L38 [Source:MGI Symbol;Acc:MGI:1914921]	protein_coding
ENSMUSG00000069268	3837.110314	1.422882946	1.232558497	2.10E-04	0.005645526	H2bc7	13:23573736-23574196	H2B clustered histone 7 [Source:MGI Symbol;Acc:MGI:2448383]	protein_coding
ENSMUSG00000037563	9357.567983	1.40458435	0.735829239	6.09E-05	0.002423155	Rps16	7:28350652-28353155	ribosomal protein S16 [Source:MGI Symbol;Acc:MGI:98118]	protein_coding
ENSMUSG00000035279	614.8746546	1.401384077	0.425161118	2.23E-06	2.25E-04	Ssc5d	7:4925785-4944826	scavenger receptor cysteine rich family, 5 domains [Source:MGI Symbol;Acc:MGI:3606211]	protein_coding
ENSMUSG00000032332	24293.33053	1.390061487	0.35572842	2.84E-07	4.57E-05	Col12a1	9:79598991-79718831	collagen, type XII, alpha 1 [Source:MGI Symbol;Acc:MGI:88448]	protein_coding
ENSMUSG00000099517	2295.306243	1.382786532	1.058867962	1.80E-04	0.005106799	H3c8	13:23535422-23535951	H3 clustered histone 8 [Source:MGI Symbol;Acc:MGI:2145541]	protein_coding
ENSMUSG00000027750	19564.61416	1.379573963	0.405144619	1.70E-06	1.83E-04	Postn	3:54361109-54391037	periostin, osteoblast specific factor [Source:MGI Symbol;Acc:MGI:1926321]	protein_coding
ENSMUSG00000021702	4506.494643	1.378977979	0.540294986	1.69E-05	9.71E-04	Thbs4	13:92751590-92794818	thrombospondin 4 [Source:MGI Symbol;Acc:MGI:1101779]	protein_coding
ENSMUSG00000042436	430.0985073	1.358888354	0.659281147	4.98E-05	0.002118941	Mfap4	11:61485431-61488900	microfibrillar-associated protein 4 [Source:MGI Symbol;Acc:MGI:1342276]	protein_coding
ENSMUSG00000007594	166.5096124	1.353738697	0.316055063	7.17E-08	1.63E-05	Hapln4	8:70083457-70090862	hyaluronan and proteoglycan link protein 4 [Source:MGI Symbol;Acc:MGI:2679531]	protein_coding
ENSMUSG00000030772	1635.174827	1.346672595	0.270663359	2.93E-09	1.33E-06	Dkk3	7:112116017-112159057	dickkopf WNT signaling pathway inhibitor 3 [Source:MGI Symbol;Acc:MGI:1354952]	protein_coding
ENSMUSG00000030077	231.1986646	1.346455021	0.383551845	1.35E-06	1.54E-04	Chl1	6:103510586-103750211	cell adhesion molecule L1-like [Source:MGI Symbol;Acc:MGI:1098266]	protein_coding
ENSMUSG00000051243	153.3632008	1.337181738	0.312344171	7.75E-08	1.69E-05	Islr2	9:58196297-58204319	immunoglobulin superfamily containing leucine-rich repeat 2 [Source:MGI Symbol;Acc:MGI:2444277]	protein_coding
ENSMUSG00000024810	110.9953843	1.333574014	0.473881178	1.02E-05	6.74E-04	Il33	19:29925114-29960718	interleukin 33 [Source:MGI Symbol;Acc:MGI:1924375]	protein_coding
ENSMUSG00000099583	6013.91131	1.332011939	0.705127884	7.30E-05	0.002734216	H3c4	13:23575763-23577011	H3 clustered histone 4 [Source:MGI Symbol;Acc:MGI:2448322]	protein_coding
ENSMUSG00000052565	22537.42034	1.324556835	0.869594696	1.40E-04	0.004293218	H1f3	13:23553262-23560686	H1.3 linker histone, cluster member [Source:MGI Symbol;Acc:MGI:107502]	protein_coding
ENSMUSG00000033006	66.5718071	1.302624592	0.482997384	1.48E-05	8.70E-04	Sox10	15:79154908-79165240	SRY (sex determining region Y)-box 10 [Source:MGI Symbol;Acc:MGI:98358]	protein_coding
ENSMUSG00000016995	352.0926747	1.298717235	0.339649723	5.49E-07	7.60E-05	Matn4	2:164389393-164405160	matrilin 4 [Source:MGI Symbol;Acc:MGI:1328314]	protein_coding
ENSMUSG00000090733	7375.061283	1.271589088	0.597527672	5.37E-05	0.002237607	Rps27	3:90212522-90213651	ribosomal protein S27 [Source:MGI Symbol;Acc:MGI:1888676]	protein_coding
ENSMUSG00000071656	282.7017087	1.271180608	0.503524381	2.40E-05	0.001249532	Lrrn4cl	19:8850768-8853909	LRRN4 C-terminal like [Source:MGI Symbol;Acc:MGI:1916102]	protein_coding
ENSMUSG00000004105	2662.428693	1.267784856	0.277621365	2.99E-08	9.33E-06	Angptl2	2:33216069-33247717	angiopoietin-like 2 [Source:MGI Symbol;Acc:MGI:1347002]	protein_coding
ENSMUSG00000114456	5765.460369	1.257786846	0.703033183	1.03E-04	0.003514288	H2bc9	13:23541714-23543392	H2B clustered histone 9 [Source:MGI Symbol;Acc:MGI:2448387]	protein_coding
ENSMUSG00000025140	573.527611	1.257180309	0.196364216	1.18E-12	1.52E-09	Pycr1	11:120635712-120643769	pyrroline-5-carboxylate reductase 1 [Source:MGI Symbol;Acc:MGI:2384795]	protein_coding
ENSMUSG00000015852	276.5612624	1.252397771	0.238266919	1.06E-09	5.73E-07	Fcrls	3:87250758-87263738	Fc receptor-like S, scavenger receptor [Source:MGI Symbol;Acc:MGI:1933397]	protein_coding
ENSMUSG00000069303	422.9587151	1.251134168	1.148094	3.01E-04	0.007231705	Hist1h2br	13:21833743-21837530	histone cluster 1 H2br [Source:MGI Symbol;Acc:MGI:3710645]	protein_coding
ENSMUSG00000071516	3384.094623	1.247294858	1.225550621	3.27E-04	0.007577996	H2ac13	13:21716422-21716814	H2A clustered histone 13 [Source:MGI Symbol;Acc:MGI:2448457]	protein_coding
ENSMUSG00000068794	71.64363608	1.232137039	0.390262567	5.79E-06	4.32E-04	Col28a1	6:7997808-8192617	collagen, type XXVIII, alpha 1 [Source:MGI Symbol;Acc:MGI:2685312]	protein_coding
ENSMUSG00000030268	448.7247967	1.221153263	0.160334568	2.56E-16	1.12E-12	Bcat1	6:144993835-145076184	branched chain aminotransferase 1, cytosolic [Source:MGI Symbol;Acc:MGI:104861]	protein_coding
ENSMUSG00000031740	12130.60399	1.210601312	0.240743277	4.23E-09	1.78E-06	Mmp2	8:92827291-92853420	matrix metallopeptidase 2 [Source:MGI Symbol;Acc:MGI:97009]	protein_coding
ENSMUSG00000032997	1861.672124	1.200730713	0.178758749	1.93E-13	3.84E-10	Chpf	1:75474569-75479307	chondroitin polymerizing factor [Source:MGI Symbol;Acc:MGI:106576]	protein_coding
ENSMUSG00000056174	1107.061987	1.196348257	0.334833171	1.92E-06	2.00E-04	Col8a2	4:126286793-126314330	collagen, type VIII, alpha 2 [Source:MGI Symbol;Acc:MGI:88464]	protein_coding
ENSMUSG00000049001	1450.017101	1.191078086	0.218654564	5.08E-10	3.00E-07	Ndnf	6:65671590-65712326	neuron-derived neurotrophic factor [Source:MGI Symbol;Acc:MGI:1915419]	protein_coding
ENSMUSG00000055368	195.2423494	1.189128335	0.231220815	2.61E-09	1.24E-06	Slc6a2	8:92960079-93001667	solute carrier family 6 (neurotransmitter transporter, noradrenalin), member 2 [Source:MGI Symbol;A	protein_coding
ENSMUSG00000028339	3408.555837	1.17608422	0.197544139	2.94E-11	2.57E-08	Col15a1	4:47208161-47313167	collagen, type XV, alpha 1 [Source:MGI Symbol;Acc:MGI:88449]	protein_coding
ENSMUSG00000042377	109.1270789	1.175138219	0.269461496	1.12E-07	2.24E-05	Fam83g	11:61684091-61709951	family with sequence similarity 83, member G [Source:MGI Symbol;Acc:MGI:1916890]	protein_coding
ENSMUSG00000039518	62.91426031	1.172626201	0.896457056	2.63E-04	0.006605705	Cdsn	17:35552128-35557180	corneodesmosin [Source:MGI Symbol;Acc:MGI:3505689]	protein_coding
ENSMUSG00000025964	137.7385314	1.17064288	0.247075715	2.11E-08	7.10E-06	Adam23	1:63445891-63596276	a disintegrin and metallopeptidase domain 23 [Source:MGI Symbol;Acc:MGI:1345162]	protein_coding
ENSMUSG00000022754	416.3373297	1.166281263	0.188546299	7.39E-12	7.39E-09	Tmem45a	16:56805161-56886166	transmembrane protein 45a [Source:MGI Symbol;Acc:MGI:1913122]	protein_coding
ENSMUSG00000039114	69.33094266	1.166257208	0.397803852	1.30E-05	7.90E-04	Nrn1	13:36725361-36735131	neuritin 1 [Source:MGI Symbol;Acc:MGI:1915654]	protein_coding
ENSMUSG00000031595	625.5082343	1.159455575	0.280781484	3.08E-07	4.85E-05	Pdgfrl	8:40926212-40990785	platelet-derived growth factor receptor-like [Source:MGI Symbol;Acc:MGI:1916047]	protein_coding
ENSMUSG00000052276	361.2325321	1.155614173	0.518345811	6.13E-05	0.002423155	Ostn	16:27307489-27351212	osteocrin [Source:MGI Symbol;Acc:MGI:2677164]	protein_coding
ENSMUSG00000028495	6884.74559	1.152049365	0.649409801	1.37E-04	0.004243025	Rps6	4:86854660-86857412	ribosomal protein S6 [Source:MGI Symbol;Acc:MGI:98159]	protein_coding
ENSMUSG00000060572	508.5020003	1.143703761	0.206695442	3.96E-10	2.47E-07	Mfap2	4:141010418-141015984	microfibrillar-associated protein 2 [Source:MGI Symbol;Acc:MGI:99559]	protein_coding

ENSMUSG00000021974	78.20034316	1.139332202	0.314637098	2.06E-06	2.11E-04	Fgf9	14:58070547-58112720	fibroblast growth factor 9 [Source:MGI Symbol;Acc:MGI:104723]	protein_coding
ENSMUSG00000047976	96.96369743	1.138600206	0.619079864	1.28E-04	0.004053944	Kcna1	6:126640397-126646384	potassium voltage-gated channel, shaker-related subfamily, member 1 [Source:MGI Symbol;Acc:MGI:	protein_coding
ENSMUSG00000069301	3377.854091	1.138126569	0.998964079	3.52E-04	0.007962017	H2ac11	13:22042460-22042944	H2A clustered histone 11 [Source:MGI Symbol;Acc:MGI:2448293]	protein_coding
ENSMUSG00000027015	349.2990749	1.134894897	0.255782478	9.66E-08	1.99E-05	Cybrd1	2:71117975-71142926	cytochrome b reductase 1 [Source:MGI Symbol;Acc:MGI:2654575]	protein_coding
ENSMUSG00000043681	55.01811211	1.129732075	1.831356565	3.50E-04	0.007950027	Fam25c	14:34351881-34355433	family with sequence similarity 25, member C [Source:MGI Symbol;Acc:MGI:1916384]	protein_coding
ENSMUSG00000029307	17087.21026	1.129397834	0.306393409	1.73E-06	1.85E-04	Dmp1	5:104202613-104214102	dentin matrix protein 1 [Source:MGI Symbol;Acc:MGI:94910]	protein_coding
ENSMUSG00000030116	349.0968699	1.124866321	0.684974396	1.82E-04	0.005129051	Mfap5	6:122505845-122529290	microfibrillar associated protein 5 [Source:MGI Symbol;Acc:MGI:1354387]	protein_coding
ENSMUSG00000059022	56.89265104	1.117670512	0.485928168	6.04E-05	0.002410659	Kcp	6:29473162-29507952	kielin/chordin-like protein [Source:MGI Symbol;Acc:MGI:2141640]	protein_coding
ENSMUSG00000038600	78.75153253	1.116581789	0.361077263	1.04E-05	6.76E-04	Atp6v0a4	6:38048483-38124586	ATPase, H+ transporting, lysosomal V0 subunit A4 [Source:MGI Symbol;Acc:MGI:2153480]	protein_coding
ENSMUSG00000069302	1513.46774	1.111124592	1.151154051	4.45E-04	0.009255531	H2ac12	13:22035164-22035568	H2A clustered histone 12 [Source:MGI Symbol;Acc:MGI:2448295]	protein_coding
ENSMUSG00000021388	3792.723088	1.107939282	0.511118847	7.97E-05	0.002919533	Aspn	13:49544443-49567565	asporin [Source:MGI Symbol;Acc:MGI:1913945]	protein_coding
ENSMUSG00000031561	671.8921719	1.100458189	0.354767257	1.07E-05	6.97E-04	Tenm3	8:48227682-48843951	teneurin transmembrane protein 3 [Source:MGI Symbol;Acc:MGI:1345183]	protein_coding
ENSMUSG00000034161	167.2400129	1.100442672	0.359761897	1.20E-05	7.51E-04	Scx	15:76457452-76459458	scleraxis [Source:MGI Symbol;Acc:MGI:102934]	protein_coding
ENSMUSG00000024593	1066.068549	1.095179922	0.247363675	1.20E-07	2.34E-05	Megf10	18:57133090-57297467	multiple EGF-like-domains 10 [Source:MGI Symbol;Acc:MGI:2685177]	protein_coding
ENSMUSG00000056569	735.2501698	1.089677921	0.892198326	3.54E-04	0.007962017	Mpz	1:171150711-171161130	myelin protein zero [Source:MGI Symbol;Acc:MGI:103177]	protein_coding
ENSMUSG00000037411	825.3361371	1.07692301	0.395714584	2.90E-05	0.001428155	Serpine1	5:137061504-137072268	serine (or cysteine) peptidase inhibitor, clade E, member 1 [Source:MGI Symbol;Acc:MGI:97608]	protein_coding
ENSMUSG00000033207	993.9141319	1.06831435	0.234271045	7.54E-08	1.67E-05	Mamdc2	19:23302609-23448442	MAM domain containing 2 [Source:MGI Symbol;Acc:MGI:1918988]	protein_coding
ENSMUSG00000062382	285.3004883	1.065803887	0.753211242	2.94E-04	0.007135454	Ftl1-ps1	13:74399694-74407346	ferritin light polypeptide 1, pseudogene 1 [Source:MGI Symbol;Acc:MGI:3779109]	protein_coding
ENSMUSG00000017724	72.28902644	1.065226502	0.28846675	2.17E-06	2.22E-04	Etv4	11:101769742-101785371	ets variant 4 [Source:MGI Symbol;Acc:MGI:99423]	protein_coding
ENSMUSG00000046807	190.6838476	1.063032473	0.179191815	5.72E-11	4.64E-08	Lrrc75b	10:75550123-75560330	leucine rich repeat containing 75B [Source:MGI Symbol;Acc:MGI:2143657]	protein_coding
ENSMUSG00000061991	9124.241425	1.050679804	1.073711154	5.01E-04	0.00999841	H2ac10	13:23533906-23534304	H2A clustered histone 10 [Source:MGI Symbol;Acc:MGI:2448309]	protein_coding
ENSMUSG00000037568	105.6963344	1.049306304	0.160930515	1.49E-12	1.82E-09	Vash2	1:190947646-190979296	vasohibin 2 [Source:MGI Symbol;Acc:MGI:2444826]	protein_coding
ENSMUSG00000053626	89.55748963	1.047832317	0.578184137	1.76E-04	0.00503002	Tll1	8:64014931-64206271	tolloid-like [Source:MGI Symbol;Acc:MGI:106923]	protein_coding
ENSMUSG00000041144	57.58167468	1.044589362	0.368500451	2.51E-05	0.001288254	Dnah7b	1:46066315-46373550	dynein, axonemal, heavy chain 7B [Source:MGI Symbol;Acc:MGI:2684953]	protein_coding
ENSMUSG00000050700	87.46187892	1.041382481	0.297702963	4.38E-06	3.55E-04	Emilin3	2:160906437-160912328	elastin microfibril interfacer 3 [Source:MGI Symbol;Acc:MGI:2389142]	protein_coding
ENSMUSG00000049404	78.0415607	1.040937941	0.245446609	3.25E-07	5.01E-05	Rarres1	3:67478972-67515523	retinoic acid receptor responder (tazarotene induced) 1 [Source:MGI Symbol;Acc:MGI:1924461]	protein_coding
ENSMUSG00000062380	87.39349104	1.037758474	0.281479386	2.45E-06	2.40E-04	Tubb3	8:123411424-123422015	tubulin, beta 3 class III [Source:MGI Symbol;Acc:MGI:107813]	protein_coding
ENSMUSG00000016262	461.4208748	1.033531233	0.193261569	1.83E-09	9.29E-07	Sertad4	1:192844488-192855756	SERTA domain containing 4 [Source:MGI Symbol;Acc:MGI:2443496]	protein_coding
ENSMUSG00000069272	4014.304982	1.032963311	0.774581088	3.57E-04	0.007989116	H2ac8	13:23570517-23571220	H2A clustered histone 8 [Source:MGI Symbol;Acc:MGI:2448290]	protein_coding
ENSMUSG00000038274	6924.31771	1.032949995	0.60982802	2.20E-04	0.005826638	Fau	19:6057844-6059524	Finkel-Biskis-Reilly murine sarcoma virus (FBR-MuSV) ubiquitously expressed (fox derived) [Source:MG	protein_coding
ENSMUSG00000015085	163.7755963	1.028370168	0.348444604	2.02E-05	0.001090779	Entpd2	2:25395874-25401321	ectonucleoside triphosphate diphosphohydrolase 2 [Source:MGI Symbol;Acc:MGI:1096863]	protein_coding
ENSMUSG00000030606	52.11070019	1.027306545	0.275650175	2.24E-06	2.25E-04	Hapln3	7:79115102-79131018	hyaluronan and proteoglycan link protein 3 [Source:MGI Symbol;Acc:MGI:1914916]	protein_coding
ENSMUSG00000027412	180.5539454	1.026989337	0.222598535	7.08E-08	1.63E-05	Lpin3	2:160880670-160906002	lipin 3 [Source:MGI Symbol;Acc:MGI:1891342]	protein_coding
ENSMUSG00000036334	1747.336886	1.021925301	0.228412478	1.34E-07	2.52E-05	Igsf10	3:59316735-59344394	immunoglobulin superfamily, member 10 [Source:MGI Symbol;Acc:MGI:1923481]	protein_coding
ENSMUSG00000034040	77.14929243	1.00855448	0.427171037	7.75E-05	0.002858307	Galnt17	5:130872082-131308497	polypeptide N-acetylgalactosaminyltransferase 17 [Source:MGI Symbol;Acc:MGI:2137594]	protein_coding
ENSMUSG00000010830	1190.12676	1.004124495	0.211223092	4.10E-08	1.14E-05	Kdelr3	15:79516411-79527739	KDEL (Lys-Asp-Glu-Leu) endoplasmic reticulum protein retention receptor 3 [Source:MGI Symbol;Acc:	protein_coding
ENSMUSG00000039904	52.82800508	1.002361183	0.31077022	1.10E-05	7.11E-04	Gpr37	6:25665878-25690729	G protein-coupled receptor 37 [Source:MGI Symbol;Acc:MGI:1313297]	protein_coding
ENSMUSG00000032816	863.3336031	1.000680916	0.242836222	6.10E-07	8.15E-05	Igdcc4	9:65101486-65137940	immunoglobulin superfamily, DCC subclass, member 4 [Source:MGI Symbol;Acc:MGI:1858497]	protein_coding
ENSMUSG00000039057	67.9976436	0.998497296	0.354863088	3.09E-05	0.001491267	Myo16	8:10153911-10634742	myosin XVI [Source:MGI Symbol;Acc:MGI:2685951]	protein_coding
ENSMUSG00000020473	3018.019093	0.992805088	0.249544773	1.07E-06	1.26E-04	Aebp1	11:5861947-5872088	AE binding protein 1 [Source:MGI Symbol;Acc:MGI:1197012]	protein_coding
ENSMUSG00000070469	488.1062978	0.992520973	0.227515826	2.41E-07	4.05E-05	Adamtsl3	7:82335694-82614450	ADAMTS-like 3 [Source:MGI Symbol;Acc:MGI:3028499]	protein_coding
ENSMUSG00000027864	2486.997245	0.988770159	0.185130475	2.31E-09	1.12E-06	Ptgfrn	3:101040232-101110278	prostaglandin F2 receptor negative regulator [Source:MGI Symbol;Acc:MGI:1277114]	protein_coding
ENSMUSG00000052353	1517.336475	0.986787503	0.23419872	4.49E-07	6.56E-05	Cemip	7:83932857-84086502	cell migration inducing protein, hyaluronan binding [Source:MGI Symbol;Acc:MGI:2443629]	protein_coding
ENSMUSG00000019899	1333.901462	0.9834782	0.339700795	2.70E-05	0.001364003	Lama2	10:26980036-27619758	laminin, alpha 2 [Source:MGI Symbol;Acc:MGI:99912]	protein_coding
ENSMUSG00000006057	1973.758209	0.981272342	0.476298318	1.47E-04	0.004410257	Atp5g1	11:96068852-96075670	ATP synthase, H+ transporting, mitochondrial F0 complex, subunit C1 (subunit 9) [Source:MGI Symbol	protein_coding
ENSMUSG00000001131	1616.074171	0.97951615	0.337543053	2.69E-05	0.001364003	Timp1	X:20870166-20874735	tissue inhibitor of metalloproteinase 1 [Source:MGI Symbol;Acc:MGI:98752]	protein_coding
ENSMUSG00000037868	151.8221888	0.978949323	0.208986357	6.27E-08	1.54E-05	Egr2	10:67535475-67542188	early growth response 2 [Source:MGI Symbol;Acc:MGI:95296]	protein_coding
ENSMUSG00000027254	1265.932337	0.971276149	0.201794749	3.56E-08	1.05E-05	Map1a	2:121289600-121310832	microtubule-associated protein 1 A [Source:MGI Symbol;Acc:MGI:1306776]	protein_coding
ENSMUSG00000029544	55.02810094	0.970257638	0.307280549	1.47E-05	8.62E-04	Cabp1	5:115168689-115194381	calcium binding protein 1 [Source:MGI Symbol;Acc:MGI:1352750]	protein_coding
ENSMUSG00000020142	407.7720208	0.959129684	0.181527622	3.55E-09	1.53E-06	Slc1a4	11:20302180-20332713	solute carrier family 1 (glutamate/neutral amino acid transporter), member 4 [Source:MGI Symbol;Ac	protein_coding
ENSMUSG00000016763	357.187427	0.958943032	0.331682785	2.96E-05	0.001450827	Scube1	15:83604999-83725021	signal peptide, CUB domain, EGF-like 1 [Source:MGI Symbol;Acc:MGI:1890616]	protein_coding
ENSMUSG00000029093	968.5023655	0.958590096	0.21937799	2.66E-07	4.41E-05	Sorcs2	5:36017180-36398139	sortilin-related VPS10 domain containing receptor 2 [Source:MGI Symbol;Acc:MGI:1932289]	protein_coding
ENSMUSG00000036564	587.013677	0.957675014	0.247926452	1.82E-06	1.91E-04	Ndrg4	8:95676980-95715119	N-myc downstream regulated gene 4 [Source:MGI Symbol;Acc:MGI:2384590]	protein_coding
ENSMUSG00000026837	24105.31709	0.955173258	0.197846233	3.54E-08	1.05E-05	Col5a1	2:27886425-28039514	collagen, type V, alpha 1 [Source:MGI Symbol;Acc:MGI:88457]	protein_coding
ENSMUSG00000039579	56.26055846	0.953023987	0.325825088	2.79E-05	0.001387412	Grin3a	4:49661611-49845744	glutamate receptor ionotropic, NMDA3A [Source:MGI Symbol;Acc:MGI:1933206]	protein_coding
ENSMUSG00000027560	133.5977173	0.953005754	0.29149179	1.15E-05	7.34E-04	Dok5	2:170731807-170879769	docking protein 5 [Source:MGI Symbol;Acc:MGI:1924079]	protein_coding
ENSMUSG00000027978	69.42780158	0.950895954	0.370581493	6.28E-05	0.002462896	Prss12	3:123446913-123506597	protease, serine 12 neurotrypsin (motopsin) [Source:MGI Symbol;Acc:MGI:1100881]	protein_coding
ENSMUSG00000001435	1364.699314	0.950202221	0.236026053	1.05E-06	1.24E-04	Col18a1	10:77052178-77166548	collagen, type XVIII, alpha 1 [Source:MGI Symbol;Acc:MGI:88451]	protein_coding
ENSMUSG00000027375	82.08260113	0.942055875	0.522660219	2.56E-04	0.006497971	Mal	2:127633226-127656695	myelin and lymphocyte protein, T cell differentiation protein [Source:MGI Symbol;Acc:MGI:892970]	protein_coding
ENSMUSG00000057969	778.0098267	0.941359211	0.198522754	5.58E-08	1.42E-05	Sema3b	9:107597674-107609229	sema domain, immunoglobulin domain (Ig), short basic domain, secreted, (semaphorin) 3B [Source:M	protein_coding
ENSMUSG00000030562	187.6460258	0.936085232	0.514453958	2.54E-04	0.006485534	Nox4	7:87246096-87398710	NADPH oxidase 4 [Source:MGI Symbol;Acc:MGI:1354184]	protein_coding
ENSMUSG00000024727	56.90318831	0.931317108	0.337423782	4.40E-05	0.001953831	Trpm6	19:18749983-18892511	transient receptor potential cation channel, subfamily M, member 6 [Source:MGI Symbol;Acc:MGI:26	protein_coding
ENSMUSG00000027313	263.1493292	0.929731558	0.205252261	1.50E-07	2.76E-05	Chac1	2:119351229-119354381	ChaC, cation transport regulator 1 [Source:MGI Symbol;Acc:MGI:1916315]	protein_coding
ENSMUSG00000022324	724.0597212	0.924009196	0.274261357	9.59E-06	6.45E-04	Matn2	15:34306677-34436273	matrilin 2 [Source:MGI Symbol;Acc:MGI:109613]	protein_coding
ENSMUSG00000042182	138.9475273	0.921880129	0.193044025	5.14E-08	1.35E-05	Bend6	1:33852052-33907816	BEN domain containing 6 [Source:MGI Symbol;Acc:MGI:2444572]	protein_coding
ENSMUSG00000029838	507.6096349	0.916005038	0.3287682	4.37E-05	0.001944102	Ptn	6:36714929-36810220	pleiotrophin [Source:MGI Symbol;Acc:MGI:97804]	protein_coding
ENSMUSG00000044674	1060.495368	0.915787462	0.215886341	5.19E-07	7.22E-05	Fzd1	5:4753839-4758035	frizzled class receptor 1 [Source:MGI Symbol;Acc:MGI:1196625]	protein_coding
ENSMUSG00000038074	863.3198202	0.915468279	0.200248032	1.32E-07	2.52E-05	Fkbp14	6:54577604-54597308	FK506 binding protein 14 [Source:MGI Symbol;Acc:MGI:2387639]	protein_coding
ENSMUSG00000066842	627.8943115	0.9140717	0.241161932	2.69E-06	2.56E-04	Hmcn1	1:150562524-150993435	hemicentin 1 [Source:MGI Symbol;Acc:MGI:2685047]	protein_coding
ENSMUSG00000056481	489.8542574	0.911377356	0.392809383	1.18E-04	0.003802628	Cd248	19:5068078-5070682	CD248 antigen, endosialin [Source:MGI Symbol;Acc:MGI:1917695]	protein_coding
ENSMUSG00000061787	6251.817206	0.910837053	0.499796579	2.78E-04	0.006899026	Rps17	7:81342732-81345254	ribosomal protein S17 [Source:MGI Symbol;Acc:MGI:1309526]	protein_coding
ENSMUSG00000028031	107.1866321	0.909379402	0.544635231	3.48E-04	0.007929893	Dkk2	3:132085292-132180304	dickkopf WNT signaling pathway inhibitor 2 [Source:MGI Symbol;Acc:MGI:1890663]	protein_coding
ENSMUSG00000027188	477.2228589	0.903349044	0.331042371	5.19E-05	0.002184829	Pamr1	2:102550012-102643041	peptidase domain containing associated with muscle regeneration 1 [Source:MGI Symbol;Acc:MGI:24	protein_coding
ENSMUSG00000053198	474.0302722	0.90183731	0.36238395	8.74E-05	0.003103105	Prx	7:27497388-27520214	periaxin [Source:MGI Symbol;Acc:MGI:108176]	protein_coding
ENSMUSG00000030432	11456.85478	0.899903969	0.516767231	3.26E-04	0.007576642	Rpl28	7:4792874-4795066	ribosomal protein L28 [Source:MGI Symbol;Acc:MGI:101839]	protein_coding
ENSMUSG00000044646	1137.40559	0.895743077	0.212744453	6.27E-07	8.31E-05	Zbtb7c	18:75820178-76148564	zinc finger and BTB domain containing 7C [Source:MGI Symbol;Acc:MGI:2443302]	protein_coding
ENSMUSG00000074486	11424.94054	0.891680869	0.245089762	4.73E-06	3.76E-04	Bglap2	3:88377736-88378699	bone gamma-carboxyglutamate protein 2 [Source:MGI Symbol;Acc:MGI:88157]	protein_coding
ENSMUSG00000070436	23017.8194	0.891089558	0.184514419	4.47E-08	1.19E-05	Serpinh1	7:99345376-99353239	serine (or cysteine) peptidase inhibitor, clade H, member 1 [Source:MGI Symbol;Acc:MGI:88283]	protein_coding
ENSMUSG00000051000	573.5375354	0.890544159	0.21029626	5.80E-07	7.88E-05	Fam160a1	3:85660061-85817291	family with sequence similarity 160, member A1 [Source:MGI Symbol;Acc:MGI:2444746]	protein_coding
ENSMUSG00000028641	2342.498319	0.887371906	0.154531236	3.78E-10	2.43E-07	P3h1	4:119232915-119248975	prolyl 3-hydroxylase 1 [Source:MGI Symbol;Acc:MGI:1888921]	protein_coding
ENSMUSG00000032826	812.9855979	0.886121388	0.268186235	1.30E-05	7.87E-04	Ank2	3:126921612-127499350	ankyrin 2, brain [Source:MGI Symbol;Acc:MGI:88025]	protein_coding
ENSMUSG00000091243	114.8655955	0.885965034	0.393989153	1.48E-04	0.00442806	Vgll3	16:65815257-65866368	vestigial like family member 3 [Source:MGI Symbol;Acc:MGI:1920819]	protein_coding
ENSMUSG00000056073	55.29779113	0.885532097	0.267907782	1.29E-05	7.87E-04	Grik2	10:49094833-49788766	glutamate receptor, ionotropic, kainate 2 (beta 2) [Source:MGI Symbol;Acc:MGI:95815]	protein_coding
ENSMUSG00000035357	749.4709793	0.884091435	0.221587852	1.48E-06	1.63E-04	Pdzrn3	6:101149609-101377897	PDZ domain containing RING finger 3 [Source:MGI Symbol;Acc:MGI:1933157]	protein_coding
ENSMUSG00000041372	524.67537	0.88331105	0.241200398	4.50E-06	3.62E-04	B4galnt3	6:120203073-120294559	beta-1,4-N-acetyl-galactosaminyl transferase 3 [Source:MGI Symbol;Acc:MGI:3041155]	protein_coding
ENSMUSG00000096010	4745.880962	0.878806332	0.659586551	5.80E-04	0.010923627	H4f16	6:136801553-136804415	H4 histone 16 [Source:MGI Symbol;Acc:MGI:2448443]	protein_coding
ENSMUSG00000051048	217.5314043	0.877802293	0.315051708	4.95E-05	0.002118097	P4ha3	7:100285520-100319699	procollagen-proline, 2-oxoglutarate 4-dioxygenase (proline 4-hydroxylase), alpha polypeptide III [Sour	protein_coding
ENSMUSG00000023067	2166.222346	0.870095178	0.181279816	5.53E-08	1.42E-05	Cdkn1a	17:29090976-29100727	cyclin-dependent kinase inhibitor 1A (P21) [Source:MGI Symbol;Acc:MGI:104556]	protein_coding
ENSMUSG00000063689	419.4232323	0.867096049	0.967881356	8.44E-04	0.014134455	H2ac21	3:96219865-96220308	H2A clustered histone 21 [Source:MGI Symbol;Acc:MGI:2448314]	protein_coding
ENSMUSG00000003355	441.2742181	0.866336051	0.182263818	6.94E-08	1.63E-05	Fkbp11	15:98724366-98728198	FK506 binding protein 11 [Source:MGI Symbol;Acc:MGI:1913370]	protein_coding
ENSMUSG00000070576	754.2280105	0.864932214	0.320436832	6.29E-05	0.002462896	Mn1	5:111417362-111457033	meningioma 1 [Source:MGI Symbol;Acc:MGI:1261813]	protein_coding
ENSMUSG00000047497	634.0427422	0.863622974	0.236749354	4.99E-06	3.92E-04	Adamts12	15:11064790-11349231	a disintegrin-like and metallopeptidase (reprolysin type) with thrombospondin type 1 motif, 12 [Sourc	protein_coding
ENSMUSG00000035914	1346.391148	0.860189238	0.193585001	2.74E-07	4.48E-05	Cd276	9:58524298-58555437	CD276 antigen [Source:MGI Symbol;Acc:MGI:2183926]	protein_coding
ENSMUSG00000031253	1391.274456	0.857041331	0.20420376	7.44E-07	9.62E-05	Srpx2	X:133908426-133932446	sushi-repeat-containing protein, X-linked 2 [Source:MGI Symbol;Acc:MGI:1916042]	protein_coding
ENSMUSG00000049517	8563.776117	0.856112237	0.536923023	4.70E-04	0.00957715	Rps23	13:90922958-90924950	ribosomal protein S23 [Source:MGI Symbol;Acc:MGI:1913725]	protein_coding
ENSMUSG00000026043	40782.37868	0.855793023	0.398393325	1.99E-04	0.00544343	Col3a1	1:45311538-45349706	collagen, type III, alpha 1 [Source:MGI Symbol;Acc:MGI:88453]	protein_coding
ENSMUSG00000021950	839.1906302	0.855135237	0.394614694	1.92E-04	0.005347477	Anxa8	14:34085981-34100571	annexin A8 [Source:MGI Symbol;Acc:MGI:1201374]	protein_coding
ENSMUSG00000033361	159.0216662	0.854883234	0.410105778	2.23E-04	0.005878956	Prrg3	X:71962624-71972722	proline rich Gla (G-carboxyglutamic acid) 3 (transmembrane) [Source:MGI Symbol;Acc:MGI:2685214]	protein_coding
ENSMUSG00000057719	125.7919771	0.853713337	0.633506103	6.26E-04	0.011451464	Sh3rf2	18:42053667-42158960	SH3 domain containing ring finger 2 [Source:MGI Symbol;Acc:MGI:2444628]	protein_coding
ENSMUSG00000038539	1898.802784	0.852356562	0.148604875	4.49E-10	2.73E-07	Atf5	7:44812256-44816658	activating transcription factor 5 [Source:MGI Symbol;Acc:MGI:2141857]	protein_coding
ENSMUSG00000027797	616.1226134	0.843743837	0.178559379	8.46E-08	1.79E-05	Dclk1	3:55242364-55539068	doublecortin-like kinase 1 [Source:MGI Symbol;Acc:MGI:1330861]	protein_coding
ENSMUSG00000052504	343.8308224	0.843235904	0.3397217	1.08E-04	0.003614228	Epha3	16:63543534-63864175	Eph receptor A3 [Source:MGI Symbol;Acc:MGI:99612]	protein_coding
ENSMUSG00000032518	25853.49517	0.842284714	0.480812727	4.01E-04	0.008620695	Rpsa	9:120127689-120132369	ribosomal protein SA [Source:MGI Symbol;Acc:MGI:105381]	protein_coding
ENSMUSG00000062328	8619.900658	0.840567143	0.52202996	4.90E-04	0.009832579	Rpl17	18:74998558-75003381	ribosomal protein L17 [Source:MGI Symbol;Acc:MGI:2448270]	protein_coding
ENSMUSG00000022510	57.29443252	0.839596293	0.509484516	4.68E-04	0.009547229	Trp63	16:25683763-25892102	transformation related protein 63 [Source:MGI Symbol;Acc:MGI:1330810]	protein_coding
ENSMUSG00000058546	7170.620378	0.837354982	0.528560263	5.13E-04	0.010153011	Rpl23a	11:78180932-78183584	ribosomal protein L23A [Source:MGI Symbol;Acc:MGI:3040672]	protein_coding
ENSMUSG00000035095	700.7163952	0.833211867	0.201234557	9.85E-07	1.21E-04	Fam167a	14:63436394-63465498	family with sequence similarity 167, member A [Source:MGI Symbol;Acc:MGI:3606565]	protein_coding
ENSMUSG00000032911	3216.365838	0.831705834	0.2681683	2.62E-05	0.001335598	Cspg4	9:56865033-56899870	chondroitin sulfate proteoglycan 4 [Source:MGI Symbol;Acc:MGI:2153093]	protein_coding
ENSMUSG00000018604	208.833109	0.83141879	0.334533489	1.12E-04	0.003686418	Tbx3	5:119670669-119684724	T-box 3 [Source:MGI Symbol;Acc:MGI:98495]	protein_coding
ENSMUSG00000059901	981.879725	0.828123724	0.217881751	3.34E-06	2.98E-04	Adamts14	10:61197112-61273438	a disintegrin-like and metallopeptidase (reprolysin type) with thrombospondin type 1 motif, 14 [Sourc	protein_coding
ENSMUSG00000046491	94.57192178	0.827973897	0.437117446	3.41E-04	0.007814572	C1qtnf2	11:43474276-43491525	C1q and tumor necrosis factor related protein 2 [Source:MGI Symbol;Acc:MGI:1916433]	protein_coding
ENSMUSG00000062683	1243.043651	0.827665086	0.451261693	3.75E-04	0.008313044	Atp5g2	15:102662864-102672352	ATP synthase, H+ transporting, mitochondrial F0 complex, subunit C2 (subunit 9) [Source:MGI Symbol	protein_coding
ENSMUSG00000023336	281.5864495	0.825908875	0.414118958	2.91E-04	0.007104642	Wfdc1	8:119666365-119688222	WAP four-disulfide core domain 1 [Source:MGI Symbol;Acc:MGI:1915116]	protein_coding
ENSMUSG00000048583	66.20743426	0.823824169	0.546412849	5.94E-04	0.011054611	Igf2	7:142650766-142666816	insulin-like growth factor 2 [Source:MGI Symbol;Acc:MGI:96434]	protein_coding
ENSMUSG00000069920	1153.531353	0.823621019	0.18547581	3.08E-07	4.85E-05	B3gnt9	8:105252638-105255153	UDP-GlcNAc:betaGal beta-1,3-N-acetylglucosaminyltransferase 9 [Source:MGI Symbol;Acc:MGI:2142	protein_coding
ENSMUSG00000029070	5357.005567	0.822382731	0.197475856	9.26E-07	1.16E-04	Mxra8	4:155839680-155844088	matrix-remodelling associated 8 [Source:MGI Symbol;Acc:MGI:1922011]	protein_coding
ENSMUSG00000041559	7548.348628	0.822298388	0.45676617	4.04E-04	0.008620695	Fmod	1:134037254-134048277	fibromodulin [Source:MGI Symbol;Acc:MGI:1328364]	protein_coding
ENSMUSG00000043903	2346.240304	0.822021454	0.283115552	4.44E-05	0.001957505	Zfp469	8:122258620-122272650	zinc finger protein 469 [Source:MGI Symbol;Acc:MGI:2684868]	protein_coding
ENSMUSG00000013419	1107.892975	0.820548541	0.200641095	1.23E-06	1.43E-04	Zfp651	9:121759330-121771742	zinc finger protein 651 [Source:MGI Symbol;Acc:MGI:2670992]	protein_coding
ENSMUSG00000013089	859.2326573	0.819838969	0.180755023	2.09E-07	3.65E-05	Etv5	16:22381309-22439719	ets variant 5 [Source:MGI Symbol;Acc:MGI:1096867]	protein_coding
ENSMUSG00000019853	115.9019456	0.818623464	0.183321852	2.82E-07	4.56E-05	Hebp2	10:18539498-18546076	heme binding protein 2 [Source:MGI Symbol;Acc:MGI:1860084]	protein_coding
ENSMUSG00000000223	393.0610168	0.818210092	0.45181896	4.04E-04	0.008620695	Drp2	X:134404551-134456573	dystrophin related protein 2 [Source:MGI Symbol;Acc:MGI:107432]	protein_coding
ENSMUSG00000031149	364.5259155	0.817340237	0.127302608	7.78E-12	7.39E-09	Praf2	X:7728439-7731064	PRA1 domain family 2 [Source:MGI Symbol;Acc:MGI:1859607]	protein_coding
ENSMUSG00000031286	116.2637908	0.816196869	0.180930269	2.34E-07	3.97E-05	Glt28d2	3:85869849-85887516	glycosyltransferase 28 domain containing 2 [Source:MGI Symbol;Acc:MGI:2443773]	protein_coding
ENSMUSG00000039252	176.0871896	0.816151782	0.497324197	5.19E-04	0.010220887	Lgi2	5:52533517-52566462	leucine-rich repeat LGI family, member 2 [Source:MGI Symbol;Acc:MGI:2180196]	protein_coding
ENSMUSG00000039883	262.8352093	0.814509718	0.322531978	1.08E-04	0.003614228	Lrrc17	5:21543559-21575904	leucine rich repeat containing 17 [Source:MGI Symbol;Acc:MGI:1921761]	protein_coding

ENSMUSG00000054555	2738.300515	0.812149069	0.17228949	9.77E-08	2.00E-05	Adam12	7:133883199-134232146	a disintegrin and metallopeptidase domain 12 (meltrin alpha) [Source:MGI Symbol;Acc:MGI:105378]	protein_coding
ENSMUSG00000064080	1570.043588	0.810873111	0.354471593	1.79E-04	0.005083949	Fbln2	6:91212455-91272540	fibulin 2 [Source:MGI Symbol;Acc:MGI:95488]	protein_coding
ENSMUSG00000001555	4933.822226	0.810286305	0.195337544	1.01E-06	1.23E-04	Fkbp10	11:100415697-100424824	FK506 binding protein 10 [Source:MGI Symbol;Acc:MGI:104769]	protein_coding
ENSMUSG00000018217	1816.013106	0.809518148	0.300743157	7.60E-05	0.002821458	Pmp22	11:63128982-63159547	peripheral myelin protein 22 [Source:MGI Symbol;Acc:MGI:97631]	protein_coding
ENSMUSG00000022012	128.7234465	0.808283764	0.197738979	1.28E-06	1.48E-04	Enox1	14:77156763-77721760	ecto-NOX disulfide-thiol exchanger 1 [Source:MGI Symbol;Acc:MGI:2444896]	protein_coding
ENSMUSG00000036242	181.2702263	0.808110045	0.369219613	2.19E-04	0.005808376	Armh4	14:49675952-49783383	armadillo-like helical domain containing 4 [Source:MGI Symbol;Acc:MGI:1914669]	protein_coding
ENSMUSG00000029161	1501.258988	0.804952663	0.239679239	1.39E-05	8.22E-04	Cgref1	5:30933143-30945591	cell growth regulator with EF hand domain 1 [Source:MGI Symbol;Acc:MGI:1915817]	protein_coding
ENSMUSG00000057863	5429.99232	0.802572385	0.518811066	6.18E-04	0.011327118	Rpl36	17:56613416-56614243	ribosomal protein L36 [Source:MGI Symbol;Acc:MGI:1860603]	protein_coding
ENSMUSG00000067925	150.5409223	0.800376862	0.204134105	2.37E-06	2.33E-04	Rtl8a	X:53642488-53643682	retrotransposon Gag like 8A [Source:MGI Symbol;Acc:MGI:1913408]	protein_coding
ENSMUSG00000038463	4963.830795	0.797172851	0.191585102	9.97E-07	1.22E-04	Olfml2b	1:170644532-170682789	olfactomedin-like 2B [Source:MGI Symbol;Acc:MGI:2443310]	protein_coding
ENSMUSG00000028597	499.9729847	0.795494575	0.228741365	1.00E-05	6.67E-04	Gpx7	4:108400390-108406961	glutathione peroxidase 7 [Source:MGI Symbol;Acc:MGI:1914555]	protein_coding
ENSMUSG00000000957	8050.478314	0.794965567	0.190034017	9.21E-07	1.16E-04	Mmp14	14:54431612-54445364	matrix metallopeptidase 14 (membrane-inserted) [Source:MGI Symbol;Acc:MGI:101900]	protein_coding
ENSMUSG00000014602	173.3594272	0.792350887	0.287441921	6.88E-05	0.002628568	Kif1a	1:93015464-93101951	kinesin family member 1A [Source:MGI Symbol;Acc:MGI:108391]	protein_coding
ENSMUSG00000010529	146.8274779	0.786517567	0.217840663	6.81E-06	4.95E-04	Gm266	12:111484609-111485823	predicted gene 266 [Source:MGI Symbol;Acc:MGI:2685112]	protein_coding
ENSMUSG00000045871	325.9391075	0.786200974	0.345192747	1.99E-04	0.00544343	Slitrk6	14:110748580-110755149	SLIT and NTRK-like family, member 6 [Source:MGI Symbol;Acc:MGI:2443198]	protein_coding
ENSMUSG00000063142	1502.179758	0.785003504	0.259698255	3.64E-05	0.001686332	Kcnma1	14:23289431-24014491	potassium large conductance calcium-activated channel, subfamily M, alpha member 1 [Source:MGI S	protein_coding
ENSMUSG00000041453	8725.953828	0.784265932	0.473047502	5.78E-04	0.010911487	Rpl21	5:146832890-146837032	ribosomal protein L21 [Source:MGI Symbol;Acc:MGI:1278340]	protein_coding
ENSMUSG00000043943	231.6894106	0.782557041	0.153796057	1.80E-08	6.36E-06	Naalad2	9:18321951-18402995	N-acetylated alpha-linked acidic dipeptidase 2 [Source:MGI Symbol;Acc:MGI:1919810]	protein_coding
ENSMUSG00000020354	368.4054316	0.782203977	0.30196356	1.04E-04	0.003529811	Sgcd	11:46896253-47988969	sarcoglycan, delta (dystrophin-associated glycoprotein) [Source:MGI Symbol;Acc:MGI:1346525]	protein_coding
ENSMUSG00000072623	516.1922645	0.780525761	0.201816786	2.97E-06	2.75E-04	Zfp9	6:118461950-118479320	zinc finger protein 9 [Source:MGI Symbol;Acc:MGI:99210]	protein_coding
ENSMUSG00000066607	186.0688777	0.779820433	0.186382542	9.48E-07	1.18E-04	Insyn1	9:58488603-58499780	inhibitory synaptic factor 1 [Source:MGI Symbol;Acc:MGI:2442108]	protein_coding
ENSMUSG00000021464	190.4466417	0.777595171	0.237741012	1.90E-05	0.00104542	Ror2	13:53109312-53286124	receptor tyrosine kinase-like orphan receptor 2 [Source:MGI Symbol;Acc:MGI:1347521]	protein_coding
ENSMUSG00000026586	4333.000827	0.777281448	0.174328835	3.20E-07	4.96E-05	Prrx1	1:163245119-163313710	paired related homeobox 1 [Source:MGI Symbol;Acc:MGI:97712]	protein_coding
ENSMUSG00000069309	1383.722859	0.775596437	0.92762347	0.001141123	0.017183641	Hist1h2an	13:21786826-21787218	histone cluster 1, H2an [Source:MGI Symbol;Acc:MGI:2448300]	protein_coding
ENSMUSG00000036957	136.9410763	0.773041443	0.208643517	5.18E-06	4.03E-04	Lrfn3	7:30355489-30362772	leucine rich repeat and fibronectin type III domain containing 3 [Source:MGI Symbol;Acc:MGI:244251	protein_coding
ENSMUSG00000026828	396.4956279	0.771431953	0.257895592	4.08E-05	0.001829838	Galnt5	2:57997884-58045860	polypeptide N-acetylgalactosaminyltransferase 5 [Source:MGI Symbol;Acc:MGI:2179403]	protein_coding
ENSMUSG00000029436	163.5641175	0.769758756	0.200869625	3.42E-06	3.02E-04	Mmp17	5:129584169-129611099	matrix metallopeptidase 17 [Source:MGI Symbol;Acc:MGI:1346076]	protein_coding
ENSMUSG00000029581	2064.687971	0.767859694	0.14319671	4.63E-09	1.87E-06	Fscn1	5:142960343-142973185	fascin actin-bundling protein 1 [Source:MGI Symbol;Acc:MGI:1352745]	protein_coding
ENSMUSG00000019539	6421.498155	0.766929429	0.177305874	5.61E-07	7.67E-05	Rcn3	7:45082913-45092221	reticulocalbin 3, EF-hand calcium binding domain [Source:MGI Symbol;Acc:MGI:1277122]	protein_coding
ENSMUSG00000026211	1115.249497	0.766612287	0.155599945	4.09E-08	1.14E-05	Obsl1	1:75479310-75506452	obscurin-like 1 [Source:MGI Symbol;Acc:MGI:2138628]	protein_coding
ENSMUSG00000044006	1514.444677	0.766032466	0.50483534	7.47E-04	0.012926304	Cilp2	8:69880369-69887687	cartilage intermediate layer protein 2 [Source:MGI Symbol;Acc:MGI:1915959]	protein_coding
ENSMUSG00000033420	4698.027939	0.765963525	0.140720133	3.03E-09	1.35E-06	Antxr1	6:87133853-87335821	anthrax toxin receptor 1 [Source:MGI Symbol;Acc:MGI:1916788]	protein_coding
ENSMUSG00000040552	434.876796	0.76442668	0.150648288	2.02E-08	6.91E-06	C3ar1	6:122847138-122856161	complement component 3a receptor 1 [Source:MGI Symbol;Acc:MGI:1097680]	protein_coding
ENSMUSG00000007279	94.27749511	0.764290985	0.462070898	6.31E-04	0.011512397	Scube2	7:109798676-109865679	signal peptide, CUB domain, EGF-like 2 [Source:MGI Symbol;Acc:MGI:1928765]	protein_coding
ENSMUSG00000020658	228.2518402	0.763494689	0.205877389	5.22E-06	4.05E-04	Efr3b	12:3962554-4038915	EFR3 homolog B [Source:MGI Symbol;Acc:MGI:2444851]	protein_coding
ENSMUSG00000015957	82.95882292	0.763045899	0.286417453	9.27E-05	0.003253328	Wnt11	7:98835112-98855195	wingless-type MMTV integration site family, member 11 [Source:MGI Symbol;Acc:MGI:101948]	protein_coding
ENSMUSG00000038552	241.0989352	0.760403768	0.230956614	1.86E-05	0.001026497	Fndc4	5:31292242-31296080	fibronectin type III domain containing 4 [Source:MGI Symbol;Acc:MGI:1917195]	protein_coding
ENSMUSG00000037613	252.9992033	0.759555344	0.129838953	3.17E-10	2.10E-07	Tnfrsf23	7:143665809-143685872	tumor necrosis factor receptor superfamily, member 23 [Source:MGI Symbol;Acc:MGI:1930269]	protein_coding
ENSMUSG00000015222	129.0959559	0.757271249	0.251086967	3.96E-05	0.001791071	Map2	1:66175273-66442583	microtubule-associated protein 2 [Source:MGI Symbol;Acc:MGI:97175]	protein_coding
ENSMUSG00000042607	77.72268672	0.756780756	0.270779564	6.92E-05	0.002638801	Asb4	6:5383386-5433022	ankyrin repeat and SOCS box-containing 4 [Source:MGI Symbol;Acc:MGI:1929751]	protein_coding
ENSMUSG00000098274	2317.205721	0.754851719	0.556352687	9.28E-04	0.015039382	Rpl24	16:55966275-55971435	ribosomal protein L24 [Source:MGI Symbol;Acc:MGI:1915443]	protein_coding
ENSMUSG00000018672	1197.398066	0.753804887	0.202392141	5.04E-06	3.95E-04	Copz2	11:96849870-96861203	coatomer protein complex, subunit zeta 2 [Source:MGI Symbol;Acc:MGI:1929008]	protein_coding
ENSMUSG00000032243	2022.418396	0.752545433	0.189657483	2.21E-06	2.24E-04	Itga11	9:62677826-62783982	integrin alpha 11 [Source:MGI Symbol;Acc:MGI:2442114]	protein_coding
ENSMUSG00000025754	125.3100472	0.750055691	0.534266171	9.06E-04	0.014812833	Agbl1	7:76229887-77124698	ATP/GTP binding protein-like 1 [Source:MGI Symbol;Acc:MGI:3646469]	protein_coding
ENSMUSG00000090862	6087.126271	0.744045494	0.461153633	7.27E-04	0.012654263	Rps13	7:116331505-116334195	ribosomal protein S13 [Source:MGI Symbol;Acc:MGI:1915302]	protein_coding
ENSMUSG00000022629	294.5393391	0.742301252	0.26336523	6.77E-05	0.002596905	Kif21a	15:90933276-91049948	kinesin family member 21A [Source:MGI Symbol;Acc:MGI:109188]	protein_coding
ENSMUSG00000050910	1449.583656	0.742178875	0.178960782	1.17E-06	1.36E-04	Cdr2l	11:115381916-115396132	cerebellar degeneration-related protein 2-like [Source:MGI Symbol;Acc:MGI:2684867]	protein_coding
ENSMUSG00000026826	404.2612484	0.741453949	0.251410042	4.89E-05	0.002098913	Nr4a2	2:57106830-57124003	nuclear receptor subfamily 4, group A, member 2 [Source:MGI Symbol;Acc:MGI:1352456]	protein_coding
ENSMUSG00000022816	10496.24054	0.737005468	0.130285572	1.01E-09	5.73E-07	Fstl1	16:37776873-37836514	follistatin-like 1 [Source:MGI Symbol;Acc:MGI:102793]	protein_coding
ENSMUSG00000061603	494.8287993	0.734224688	0.319902061	2.28E-04	0.005972592	Akap6	12:52699383-53155599	A kinase (PRKA) anchor protein 6 [Source:MGI Symbol;Acc:MGI:3050566]	protein_coding
ENSMUSG00000030256	738.2821186	0.733171741	0.188347073	2.96E-06	2.75E-04	Bhlhe41	6:145858243-145865558	basic helix-loop-helix family, member e41 [Source:MGI Symbol;Acc:MGI:1930704]	protein_coding
ENSMUSG00000034675	611.8163697	0.730902711	0.179788278	1.60E-06	1.74E-04	Dbn1	13:55473429-55488111	drebrin 1 [Source:MGI Symbol;Acc:MGI:1931838]	protein_coding
ENSMUSG00000047205	128.5491647	0.729130524	0.254456416	6.22E-05	0.00245348	Dusp18	11:3895240-3901296	dual specificity phosphatase 18 [Source:MGI Symbol;Acc:MGI:1922469]	protein_coding
ENSMUSG00000029335	4049.461861	0.728819419	0.254661922	6.28E-05	0.002462896	Bmp3	5:98854415-98884396	bone morphogenetic protein 3 [Source:MGI Symbol;Acc:MGI:88179]	protein_coding
ENSMUSG00000051065	133.1097713	0.726621138	0.199480013	6.87E-06	4.97E-04	Mb21d2	16:28826176-28929673	Mab-21 domain containing 2 [Source:MGI Symbol;Acc:MGI:1917028]	protein_coding
ENSMUSG00000005958	314.8885309	0.725892614	0.262568665	8.04E-05	0.002930856	Ephb3	16:21204755-21223305	Eph receptor B3 [Source:MGI Symbol;Acc:MGI:104770]	protein_coding
ENSMUSG00000039683	85.54823238	0.72487023	0.390776641	5.44E-04	0.010513273	Sdk1	5:141241490-142215586	sidekick cell adhesion molecule 1 [Source:MGI Symbol;Acc:MGI:2444413]	protein_coding
ENSMUSG00000040711	5985.689897	0.724111369	0.207753123	1.13E-05	7.22E-04	Sh3pxd2b	11:32347820-32428173	SH3 and PX domains 2B [Source:MGI Symbol;Acc:MGI:2442062]	protein_coding
ENSMUSG00000039476	179.6057189	0.72349326	0.353468599	3.87E-04	0.008469529	Prrx2	2:30834972-30881251	paired related homeobox 2 [Source:MGI Symbol;Acc:MGI:98218]	protein_coding
ENSMUSG00000046182	73.82950355	0.721577856	0.354729198	3.99E-04	0.008620695	Gsg1l	7:125878420-126082411	GSG1-like [Source:MGI Symbol;Acc:MGI:2685483]	protein_coding
ENSMUSG00000028583	512.1677063	0.719222296	0.256677447	7.45E-05	0.002771379	Pdpn	4:143267431-143299564	podoplanin [Source:MGI Symbol;Acc:MGI:103098]	protein_coding
ENSMUSG00000061477	8234.048128	0.718313024	0.519264253	0.00106561	0.016452554	Rps7	12:28630854-28635953	ribosomal protein S7 [Source:MGI Symbol;Acc:MGI:1333818]	protein_coding
ENSMUSG00000030739	139.0569457	0.717535808	0.479189028	9.46E-04	0.015192294	Myh14	7:44605803-44670843	myosin, heavy polypeptide 14 [Source:MGI Symbol;Acc:MGI:1919210]	protein_coding
ENSMUSG00000032942	265.8385832	0.715928829	0.54532654	0.001147936	0.017274344	Ucp3	7:100472990-100486432	uncoupling protein 3 (mitochondrial, proton carrier) [Source:MGI Symbol;Acc:MGI:1099787]	protein_coding
ENSMUSG00000026235	202.3640725	0.714810155	0.254742263	7.45E-05	0.002771379	Epha4	1:77367185-77515088	Eph receptor A4 [Source:MGI Symbol;Acc:MGI:98277]	protein_coding
ENSMUSG00000040158	252.4405651	0.71362951	0.27145309	1.15E-04	0.00373588	Tax1bp3	11:73177083-73183162	Tax1 (human T cell leukemia virus type I) binding protein 3 [Source:MGI Symbol;Acc:MGI:1923531]	protein_coding
ENSMUSG00000048126	12652.86553	0.712552863	0.238143415	4.65E-05	0.002033313	Col6a3	1:90765923-90843971	collagen, type VI, alpha 3 [Source:MGI Symbol;Acc:MGI:88461]	protein_coding
ENSMUSG00000042104	335.7936245	0.712458783	0.151944338	1.33E-07	2.52E-05	Uggt2	14:118985039-119099430	UDP-glucose glycoprotein glucosyltransferase 2 [Source:MGI Symbol;Acc:MGI:1913685]	protein_coding
ENSMUSG00000028763	20232.06732	0.712228433	0.223445257	2.74E-05	0.001370269	Hspg2	4:137468769-137570630	perlecan (heparan sulfate proteoglycan 2) [Source:MGI Symbol;Acc:MGI:96257]	protein_coding
ENSMUSG00000020857	562.9385426	0.711921749	0.347281681	4.01E-04	0.008620695	Nme2	11:93949814-93956259	NME/NM23 nucleoside diphosphate kinase 2 [Source:MGI Symbol;Acc:MGI:97356]	protein_coding
ENSMUSG00000060126	20842.91716	0.704238988	0.457740188	9.54E-04	0.015294226	Tpt1	14:75845093-75848525	tumor protein, translationally-controlled 1 [Source:MGI Symbol;Acc:MGI:104890]	protein_coding
ENSMUSG00000025856	1503.760354	0.703970386	0.183242657	3.65E-06	3.12E-04	Pdgfa	5:138976014-138997370	platelet derived growth factor, alpha [Source:MGI Symbol;Acc:MGI:97527]	protein_coding
ENSMUSG00000028885	120.8955461	0.70304448	0.160601691	4.97E-07	7.02E-05	Smpdl3b	4:132732966-132757252	sphingomyelin phosphodiesterase, acid-like 3B [Source:MGI Symbol;Acc:MGI:1916022]	protein_coding
ENSMUSG00000109946	85.97099009	0.699736731	0.188669002	5.73E-06	4.30E-04	Brd3os	2:27442324-27444221	bromodomain containing 3, opposite strand [Source:MGI Symbol;Acc:MGI:106557]	protein_coding
ENSMUSG00000072812	535.3798754	0.696990344	0.396379577	7.14E-04	0.012464207	Ahnak2	12:112772194-112802657	AHNAK nucleoprotein 2 [Source:MGI Symbol;Acc:MGI:2144831]	protein_coding
ENSMUSG00000027661	360.9899666	0.695897867	0.186731265	5.42E-06	4.19E-04	Slc2a10	2:165503787-165519917	solute carrier family 2 (facilitated glucose transporter), member 10 [Source:MGI Symbol;Acc:MGI:215	protein_coding
ENSMUSG00000059291	8071.861607	0.695751403	0.423188826	8.57E-04	0.014286561	Rpl11	4:136028265-136053428	ribosomal protein L11 [Source:MGI Symbol;Acc:MGI:1914275]	protein_coding
ENSMUSG00000047238	291.7597136	0.695200912	0.210803964	2.05E-05	0.001103053	Mageh1	X:153036166-153037575	melanoma antigen, family H, 1 [Source:MGI Symbol;Acc:MGI:1922875]	protein_coding
ENSMUSG00000051146	60.41457054	0.692973414	0.201512461	1.35E-05	8.07E-04	Camk2n2	16:20619215-20621287	calcium/calmodulin-dependent protein kinase II inhibitor 2 [Source:MGI Symbol;Acc:MGI:1920297]	protein_coding
ENSMUSG00000029675	1238.130124	0.689993778	0.312375349	3.13E-04	0.00741282	Eln	5:134702593-134747323	elastin [Source:MGI Symbol;Acc:MGI:95317]	protein_coding
ENSMUSG00000031548	765.8149502	0.688848567	0.244411412	7.64E-05	0.002826828	Sfrp1	8:23411502-23449632	secreted frizzled-related protein 1 [Source:MGI Symbol;Acc:MGI:892014]	protein_coding
ENSMUSG00000030499	457.4775929	0.687224166	0.257011031	1.10E-04	0.003650325	Kctd15	7:34639014-34656732	potassium channel tetramerisation domain containing 15 [Source:MGI Symbol;Acc:MGI:2385276]	protein_coding
ENSMUSG00000044807	659.2699533	0.6859685	0.184211271	5.52E-06	4.25E-04	Zfp354c	11:50811086-50827724	zinc finger protein 354C [Source:MGI Symbol;Acc:MGI:1353621]	protein_coding
ENSMUSG00000044550	54.58291654	0.685216863	0.274097896	1.67E-04	0.004861789	Tceal3	X:136590842-136668378	transcription elongation factor A (SII)-like 3 [Source:MGI Symbol;Acc:MGI:1913354]	protein_coding
ENSMUSG00000053460	1250.960286	0.685068166	0.129668092	8.04E-09	3.20E-06	Ggcx	6:72414308-72430712	gamma-glutamyl carboxylase [Source:MGI Symbol;Acc:MGI:1927655]	protein_coding
ENSMUSG00000052727	1791.165443	0.684695168	0.257377364	1.14E-04	0.003727349	Map1b	13:99421446-99516540	microtubule-associated protein 1B [Source:MGI Symbol;Acc:MGI:1306778]	protein_coding
ENSMUSG00000006931	1117.734124	0.683866538	0.206172383	1.97E-05	0.001077231	P3h4	11:100408456-100414836	prolyl 3-hydroxylase family member 4 (non-enzymatic) [Source:MGI Symbol;Acc:MGI:1913430]	protein_coding
ENSMUSG00000002265	1303.883569	0.68374076	0.338908129	4.71E-04	0.009593676	Peg3	7:6703892-6730431	paternally expressed 3 [Source:MGI Symbol;Acc:MGI:104748]	protein_coding
ENSMUSG00000003070	76.77210872	0.683664188	0.190529422	8.59E-06	5.94E-04	Efna2	10:80179482-80190010	ephrin A2 [Source:MGI Symbol;Acc:MGI:102707]	protein_coding
ENSMUSG00000058070	960.1311323	0.682030051	0.217946012	3.38E-05	0.001601662	Eml1	12:108370957-108539617	echinoderm microtubule associated protein like 1 [Source:MGI Symbol;Acc:MGI:1915769]	protein_coding
ENSMUSG00000040310	306.757312	0.682025947	0.189089983	8.10E-06	5.73E-04	Alx4	2:93642384-93681339	aristaless-like homeobox 4 [Source:MGI Symbol;Acc:MGI:108359]	protein_coding
ENSMUSG00000070044	424.4804932	0.68110449	0.146318471	1.57E-07	2.87E-05	Fam149a	8:45336717-45382291	family with sequence similarity 149, member A [Source:MGI Symbol;Acc:MGI:2387177]	protein_coding
ENSMUSG00000104346	77.70493377	0.679464881	0.310117421	3.36E-04	0.007744622	Pcdhga3	18:37674307-37841873	protocadherin gamma subfamily A, 3 [Source:MGI Symbol;Acc:MGI:1935215]	protein_coding
ENSMUSG00000072294	430.391904	0.678659693	0.271138712	1.69E-04	0.004895031	Klf12	14:99870632-100284679	Kruppel-like factor 12 [Source:MGI Symbol;Acc:MGI:1333796]	protein_coding
ENSMUSG00000034460	316.2665278	0.672548611	0.211104301	2.92E-05	0.001435052	Six4	12:73099609-73113456	sine oculis-related homeobox 4 [Source:MGI Symbol;Acc:MGI:106034]	protein_coding
ENSMUSG00000027868	1086.15988	0.672405818	0.284842839	2.37E-04	0.006165489	Tbx15	3:99240381-99354259	T-box 15 [Source:MGI Symbol;Acc:MGI:1277234]	protein_coding
ENSMUSG00000059049	52.62883297	0.671460966	0.487048543	0.00133298	0.019174739	Frem1	4:82897920-83052339	Fras1 related extracellular matrix protein 1 [Source:MGI Symbol;Acc:MGI:2670972]	protein_coding
ENSMUSG00000026131	10045.58294	0.671093047	0.275791195	2.02E-04	0.00550651	Dst	1:33908225-34308661	dystonin [Source:MGI Symbol;Acc:MGI:104627]	protein_coding
ENSMUSG00000029913	338.6847283	0.67071251	0.186370569	8.39E-06	5.86E-04	Prdm5	6:65778988-65937010	PR domain containing 5 [Source:MGI Symbol;Acc:MGI:1918029]	protein_coding
ENSMUSG00000008682	11022.21343	0.670658137	0.405832219	9.42E-04	0.015151596	Rpl10	X:74270812-74273135	ribosomal protein L10 [Source:MGI Symbol;Acc:MGI:105943]	protein_coding
ENSMUSG00000034520	201.5197381	0.67025112	0.171977924	3.08E-06	2.82E-04	Gjc1	11:102799579-102819700	gap junction protein, gamma 1 [Source:MGI Symbol;Acc:MGI:95718]	protein_coding
ENSMUSG00000057280	68.13343549	0.670056506	0.589160061	0.001596338	0.02169294	Musk	4:58285960-58374303	muscle, skeletal, receptor tyrosine kinase [Source:MGI Symbol;Acc:MGI:103581]	protein_coding
ENSMUSG00000014303	1268.148166	0.669424213	0.171077205	2.91E-06	2.75E-04	Glis2	16:4594713-4624924	GLIS family zinc finger 2 [Source:MGI Symbol;Acc:MGI:1932535]	protein_coding
ENSMUSG00000044647	371.8922908	0.669398718	0.269740585	1.81E-04	0.005129051	Csrnp3	2:65845767-66031546	cysteine-serine-rich nuclear protein 3 [Source:MGI Symbol;Acc:MGI:1925021]	protein_coding
ENSMUSG00000041245	70.57917102	0.667935594	0.198128214	1.71E-05	9.76E-04	Wnk3	X:151198078-151320152	WNK lysine deficient protein kinase 3 [Source:MGI Symbol;Acc:MGI:2652875]	protein_coding
ENSMUSG00000053475	99.82857494	0.667162449	0.31021171	3.80E-04	0.008411094	Tnfaip6	2:52038009-52056686	tumor necrosis factor alpha induced protein 6 [Source:MGI Symbol;Acc:MGI:1195266]	protein_coding
ENSMUSG00000037206	4305.422865	0.665946221	0.167561456	2.34E-06	2.32E-04	Islr	9:58156268-58204306	immunoglobulin superfamily containing leucine-rich repeat [Source:MGI Symbol;Acc:MGI:1349645]	protein_coding
ENSMUSG00000034488	865.6821654	0.664881858	0.361599152	7.06E-04	0.012399281	Edil3	13:88821472-89323223	EGF-like repeats and discoidin I-like domains 3 [Source:MGI Symbol;Acc:MGI:1329025]	protein_coding
ENSMUSG00000039062	1013.572278	0.663766068	0.291244556	2.92E-04	0.007104642	Anpep	7:79821803-79861059	alanyl (membrane) aminopeptidase [Source:MGI Symbol;Acc:MGI:5000466]	protein_coding
ENSMUSG00000025265	511.6981528	0.663633504	0.18090172	6.72E-06	4.89E-04	Fgd1	X:151046150-151089521	FYVE, RhoGEF and PH domain containing 1 [Source:MGI Symbol;Acc:MGI:104566]	protein_coding
ENSMUSG00000047216	74.76044953	0.663379021	0.493975436	0.001434439	0.020201948	Cdh19	1:110888326-110977584	cadherin 19, type 2 [Source:MGI Symbol;Acc:MGI:3588198]	protein_coding
ENSMUSG00000042116	309.7149609	0.661707022	0.244124032	1.06E-04	0.003569747	Vwa1	4:155768149-155774698	von Willebrand factor A domain containing 1 [Source:MGI Symbol;Acc:MGI:2179729]	protein_coding
ENSMUSG00000042751	189.1286925	0.660599742	0.294457578	3.18E-04	0.007450205	Nmnat2	1:152954993-153119261	nicotinamide nucleotide adenylyltransferase 2 [Source:MGI Symbol;Acc:MGI:2444155]	protein_coding
ENSMUSG00000022440	2424.999468	0.659031898	0.162660909	1.77E-06	1.88E-04	C1qtnf6	15:78523346-78531416	C1q and tumor necrosis factor related protein 6 [Source:MGI Symbol;Acc:MGI:1919959]	protein_coding
ENSMUSG00000046743	2137.602222	0.65874393	0.267226971	1.94E-04	0.005364226	Fat4	3:38886940-39011985	FAT atypical cadherin 4 [Source:MGI Symbol;Acc:MGI:3045256]	protein_coding
ENSMUSG00000056832	122.7609607	0.658152105	0.161833867	1.67E-06	1.81E-04	Ttc26	6:38381469-38427647	tetratricopeptide repeat domain 26 [Source:MGI Symbol;Acc:MGI:2444853]	protein_coding
ENSMUSG00000012017	2825.654854	0.657987759	0.213463377	3.98E-05	0.001795134	Scarf2	16:17797282-17808293	scavenger receptor class F, member 2 [Source:MGI Symbol;Acc:MGI:1858430]	protein_coding
ENSMUSG00000027408	1265.317404	0.657358628	0.181827791	8.01E-06	5.69E-04	Cpxm1	2:130390775-130397574	carboxypeptidase X 1 (M14 family) [Source:MGI Symbol;Acc:MGI:1934569]	protein_coding
ENSMUSG00000025255	3344.420996	0.656095909	0.238447004	9.66E-05	0.00335648	Zfhx4	3:5218526-5415857	zinc finger homeodomain 4 [Source:MGI Symbol;Acc:MGI:2137668]	protein_coding
ENSMUSG00000020241	10855.11113	0.65427903	0.200082118	2.33E-05	0.001224199	Col6a2	10:76595762-76623630	collagen, type VI, alpha 2 [Source:MGI Symbol;Acc:MGI:88460]	protein_coding
ENSMUSG00000020758	641.4877223	0.652921885	0.448761429	0.001338752	0.019232461	Itgb4	11:115974709-116008412	integrin beta 4 [Source:MGI Symbol;Acc:MGI:96613]	protein_coding
ENSMUSG00000032855	8301.517856	0.652458827	0.137486788	1.05E-07	2.13E-05	Pkd1	17:24549834-24596508	polycystin 1, transient receptor poteintial channel interacting [Source:MGI Symbol;Acc:MGI:97603]	protein_coding
ENSMUSG00000023191	1448.216834	0.65194801	0.163797408	2.30E-06	2.28E-04	P3h3	6:124841089-124857752	prolyl 3-hydroxylase 3 [Source:MGI Symbol;Acc:MGI:1315208]	protein_coding
ENSMUSG00000079523	5545.666531	0.651796071	0.31117447	4.49E-04	0.009320816	Tmsb10	6:72957347-72958748	thymosin, beta 10 [Source:MGI Symbol;Acc:MGI:109146]	protein_coding
ENSMUSG00000039234	3460.381689	0.65093582	0.108491722	1.57E-10	1.14E-07	Sec24d	3:123267455-123365641	Sec24 related gene family, member D (S. cerevisiae) [Source:MGI Symbol;Acc:MGI:1916858]	protein_coding
ENSMUSG00000036995	241.5262934	0.648466159	0.281408732	2.88E-04	0.007064654	Asap3	4:136206365-136245216	ArfGAP with SH3 domain, ankyrin repeat and PH domain 3 [Source:MGI Symbol;Acc:MGI:2684986]	protein_coding

ENSMUSG00000034457	90.16104323	0.64782312	0.442677212	0.00135723	0.01942136	Eda2r	X:97333840-97377216	ectodysplasin A2 receptor [Source:MGI Symbol;Acc:MGI:2442860]	protein_coding
ENSMUSG00000096145	801.7603237	0.647808029	0.150774261	6.94E-07	9.14E-05	Vkorc1	7:127893063-127895617	vitamin K epoxide reductase complex, subunit 1 [Source:MGI Symbol;Acc:MGI:106442]	protein_coding
ENSMUSG00000028214	203.9993067	0.646448736	0.259571038	1.88E-04	0.005262611	Gem	4:11704457-11714752	GTP binding protein (gene overexpressed in skeletal muscle) [Source:MGI Symbol;Acc:MGI:99844]	protein_coding
ENSMUSG00000017446	1237.210851	0.64605586	0.146230398	4.25E-07	6.36E-05	C1qtnf1	11:118428203-118449963	C1q and tumor necrosis factor related protein 1 [Source:MGI Symbol;Acc:MGI:1919254]	protein_coding
ENSMUSG00000072825	941.9167271	0.645333737	0.194043042	1.99E-05	0.001080926	Cep170b	12:112720455-112746592	centrosomal protein 170B [Source:MGI Symbol;Acc:MGI:2145043]	protein_coding
ENSMUSG00000021318	584.5369304	0.643912769	0.213292747	4.83E-05	0.002085012	Gli3	13:15463235-15730026	GLI-Kruppel family member GLI3 [Source:MGI Symbol;Acc:MGI:95729]	protein_coding
ENSMUSG00000030717	2528.78718	0.642405814	0.245041016	1.38E-04	0.004243025	Nupr1	7:126623249-126630861	nuclear protein transcription regulator 1 [Source:MGI Symbol;Acc:MGI:1891834]	protein_coding
ENSMUSG00000031070	92.76449763	0.640705514	0.445352488	0.001446529	0.020326711	Mrgprf	7:145300828-145309557	MAS-related GPR, member F [Source:MGI Symbol;Acc:MGI:2384823]	protein_coding
ENSMUSG00000031538	342.3075664	0.638408326	0.332023381	6.65E-04	0.011979243	Plat	8:22757727-22782844	plasminogen activator, tissue [Source:MGI Symbol;Acc:MGI:97610]	protein_coding
ENSMUSG00000037016	60.75629257	0.637981704	0.43223202	0.001399179	0.01985272	Frem2	3:53513938-53657355	Fras1 related extracellular matrix protein 2 [Source:MGI Symbol;Acc:MGI:2444465]	protein_coding
ENSMUSG00000041729	300.8109143	0.637787202	0.157474914	1.77E-06	1.88E-04	Coro2b	9:62419492-62537044	coronin, actin binding protein, 2B [Source:MGI Symbol;Acc:MGI:2444283]	protein_coding
ENSMUSG00000023078	52.08428352	0.637305976	0.573820316	0.001898346	0.024373066	Cxcl13	5:95956951-95961068	chemokine (C-X-C motif) ligand 13 [Source:MGI Symbol;Acc:MGI:1888499]	protein_coding
ENSMUSG00000024427	432.8975624	0.636901376	0.192817399	2.14E-05	0.001134969	Spry4	18:38586247-38601415	sprouty RTK signaling antagonist 4 [Source:MGI Symbol;Acc:MGI:1345144]	protein_coding
ENSMUSG00000040875	56.92239292	0.636114098	0.251381067	1.74E-04	0.00503002	Osbpl10	9:114978569-115232225	oxysterol binding protein-like 10 [Source:MGI Symbol;Acc:MGI:1921736]	protein_coding
ENSMUSG00000038668	1020.062687	0.636105173	0.210050626	4.75E-05	0.002066079	Lpar1	4:58435255-58553898	lysophosphatidic acid receptor 1 [Source:MGI Symbol;Acc:MGI:108429]	protein_coding
ENSMUSG00000034780	74.76572956	0.635393088	0.351321754	8.35E-04	0.014032373	B3galt1	2:67565871-68122689	UDP-Gal:betaGlcNAc beta 1,3-galactosyltransferase, polypeptide 1 [Source:MGI Symbol;Acc:MGI:134	protein_coding
ENSMUSG00000071648	449.8417385	0.635001505	0.161127276	2.63E-06	2.54E-04	Rom1	19:8927391-8929356	rod outer segment membrane protein 1 [Source:MGI Symbol;Acc:MGI:97998]	protein_coding
ENSMUSG00000066113	258.4033542	0.63490093	0.403449906	0.001239577	0.01827919	Adamtsl1	4:85514172-86428385	ADAMTS-like 1 [Source:MGI Symbol;Acc:MGI:1924989]	protein_coding
ENSMUSG00000050288	228.5259102	0.634355202	0.316321251	5.72E-04	0.010857351	Fzd2	11:102604396-102608058	frizzled class receptor 2 [Source:MGI Symbol;Acc:MGI:1888513]	protein_coding
ENSMUSG00000028541	458.3747178	0.633841657	0.191076341	2.06E-05	0.001103053	B4galt2	4:117869260-117883487	UDP-Gal:betaGlcNAc beta 1,4- galactosyltransferase, polypeptide 2 [Source:MGI Symbol;Acc:MGI:185	protein_coding
ENSMUSG00000068220	7759.302528	0.633366999	0.111693434	1.02E-09	5.73E-07	Lgals1	15:78926725-78930465	lectin, galactose binding, soluble 1 [Source:MGI Symbol;Acc:MGI:96777]	protein_coding
ENSMUSG00000029185	1860.887058	0.633099421	0.154401725	1.46E-06	1.62E-04	Fam114a1	5:64970071-65041886	family with sequence similarity 114, member A1 [Source:MGI Symbol;Acc:MGI:1915553]	protein_coding
ENSMUSG00000042804	1579.326312	0.632339981	0.19577668	2.67E-05	0.001359672	Gpr153	4:152274232-152285337	G protein-coupled receptor 153 [Source:MGI Symbol;Acc:MGI:1916157]	protein_coding
ENSMUSG00000020814	458.9197402	0.631945083	0.24281045	1.47E-04	0.004410257	Mxra7	11:116803062-116828046	matrix-remodelling associated 7 [Source:MGI Symbol;Acc:MGI:1914872]	protein_coding
ENSMUSG00000038702	653.4286715	0.631810749	0.186758551	1.69E-05	9.70E-04	Dsel	1:111858702-111864918	dermatan sulfate epimerase-like [Source:MGI Symbol;Acc:MGI:2442948]	protein_coding
ENSMUSG00000022053	211.0705212	0.630258836	0.362801023	9.69E-04	0.015405066	Ebf2	14:67233292-67430918	early B cell factor 2 [Source:MGI Symbol;Acc:MGI:894332]	protein_coding
ENSMUSG00000054934	59.28846145	0.628784864	0.198020314	3.15E-05	0.001505333	Kcnmb4	10:116417861-116473878	potassium large conductance calcium-activated channel, subfamily M, beta member 4 [Source:MGI Sy	protein_coding
ENSMUSG00000034173	85.94509064	0.628316758	0.272353152	3.02E-04	0.007244539	Zbed5	5:129895723-129903623	zinc finger, BED type containing 5 [Source:MGI Symbol;Acc:MGI:1919220]	protein_coding
ENSMUSG00000044043	123.781771	0.627895193	0.258608319	2.28E-04	0.005972592	Pcdhb14	18:37447656-37456350	protocadherin beta 14 [Source:MGI Symbol;Acc:MGI:2136749]	protein_coding
ENSMUSG00000025321	146.0032994	0.624179381	0.336675635	8.00E-04	0.01356811	Itgb8	12:119158022-119238802	integrin beta 8 [Source:MGI Symbol;Acc:MGI:1338035]	protein_coding
ENSMUSG00000030074	2680.536089	0.6233064	0.211001584	5.93E-05	0.002390513	Gxylt2	6:100704734-100810913	glucoside xylosyltransferase 2 [Source:MGI Symbol;Acc:MGI:2682940]	protein_coding
ENSMUSG00000026062	787.0541481	0.623223633	0.229112062	1.11E-04	0.003667659	Slc9a2	1:40680574-40769273	solute carrier family 9 (sodium/hydrogen exchanger), member 2 [Source:MGI Symbol;Acc:MGI:10507	protein_coding
ENSMUSG00000102692	238.9260211	0.622832242	0.351316925	9.36E-04	0.015109994	Dchs2	3:83127948-83357209	dachsous cadherin related 2 [Source:MGI Symbol;Acc:MGI:2685263]	protein_coding
ENSMUSG00000044461	435.5706582	0.62023286	0.421466085	0.001529793	0.02106356	Shisa2	14:59625308-59631660	shisa family member 2 [Source:MGI Symbol;Acc:MGI:2444716]	protein_coding
ENSMUSG00000054793	91.5060166	0.619671742	0.436692409	0.001638607	0.022129801	Cadm4	7:24482023-24504539	cell adhesion molecule 4 [Source:MGI Symbol;Acc:MGI:2449088]	protein_coding
ENSMUSG00000067276	280.7461865	0.619021197	0.425333925	0.001571376	0.021500717	Capn6	X:143802231-143827414	calpain 6 [Source:MGI Symbol;Acc:MGI:1100850]	protein_coding
ENSMUSG00000031661	150.1059925	0.618799309	0.213172287	6.86E-05	0.00262568	Nkd1	8:88521354-88594884	naked cuticle 1 [Source:MGI Symbol;Acc:MGI:2135954]	protein_coding
ENSMUSG00000062661	164.2240618	0.618162737	0.207209567	5.49E-05	0.002262014	Ncs1	2:31245823-31295989	neuronal calcium sensor 1 [Source:MGI Symbol;Acc:MGI:109166]	protein_coding
ENSMUSG00000024998	662.9017529	0.617995237	0.251268434	2.16E-04	0.005750615	Plce1	19:38481109-38785030	phospholipase C, epsilon 1 [Source:MGI Symbol;Acc:MGI:1921305]	protein_coding
ENSMUSG00000020460	6798.336697	0.617174405	0.414044662	0.001512922	0.02093936	Rps27a	11:29545846-29548109	ribosomal protein S27A [Source:MGI Symbol;Acc:MGI:1925544]	protein_coding
ENSMUSG00000020105	497.4285684	0.616201063	0.192977578	3.01E-05	0.001472185	Lrig3	10:125966168-126015359	leucine-rich repeats and immunoglobulin-like domains 3 [Source:MGI Symbol;Acc:MGI:2443955]	protein_coding
ENSMUSG00000019891	243.0685209	0.615940812	0.18200246	1.69E-05	9.70E-04	Dcbld1	10:52233619-52321378	discoidin, CUB and LCCL domain containing 1 [Source:MGI Symbol;Acc:MGI:1913936]	protein_coding
ENSMUSG00000025089	876.4514423	0.615081315	0.182649213	1.78E-05	9.92E-04	Gfra1	19:58235604-58455909	glial cell line derived neurotrophic factor family receptor alpha 1 [Source:MGI Symbol;Acc:MGI:11008	protein_coding
ENSMUSG00000043556	289.345602	0.614108215	0.185466278	2.11E-05	0.00112413	Fbxl7	15:26540459-26895580	F-box and leucine-rich repeat protein 7 [Source:MGI Symbol;Acc:MGI:3052506]	protein_coding
ENSMUSG00000027803	1783.363321	0.612772397	0.19264137	3.13E-05	0.001502135	Wwtr1	3:57455649-57575910	WW domain containing transcription regulator 1 [Source:MGI Symbol;Acc:MGI:1917649]	protein_coding
ENSMUSG00000015653	191.0171711	0.612578123	0.216609265	8.47E-05	0.003034286	Steap2	5:5664829-5694578	six transmembrane epithelial antigen of prostate 2 [Source:MGI Symbol;Acc:MGI:1921301]	protein_coding
ENSMUSG00000027204	7855.016136	0.612477123	0.188305458	2.52E-05	0.0012927	Fbn1	2:125300594-125507993	fibrillin 1 [Source:MGI Symbol;Acc:MGI:95489]	protein_coding
ENSMUSG00000020181	851.2093718	0.611186961	0.223963722	1.10E-04	0.003658217	Nav3	10:109681259-110456204	neuron navigator 3 [Source:MGI Symbol;Acc:MGI:2183703]	protein_coding
ENSMUSG00000035305	149.7441643	0.61061464	0.276910993	3.99E-04	0.008620695	Ror1	4:100095791-100444765	receptor tyrosine kinase-like orphan receptor 1 [Source:MGI Symbol;Acc:MGI:1347520]	protein_coding
ENSMUSG00000024598	1397.270083	0.610391174	0.274339375	3.82E-04	0.008417698	Fbn2	18:58008623-58210487	fibrillin 2 [Source:MGI Symbol;Acc:MGI:95490]	protein_coding
ENSMUSG00000078794	364.9100893	0.610152999	0.211112631	7.17E-05	0.002707405	Dact3	7:16875317-16887462	dishevelled-binding antagonist of beta-catenin 3 [Source:MGI Symbol;Acc:MGI:3654828]	protein_coding
ENSMUSG00000040690	3535.109566	0.606267293	0.318557423	7.75E-04	0.013287321	Col16a1	4:130047840-130099283	collagen, type XVI, alpha 1 [Source:MGI Symbol;Acc:MGI:1095396]	protein_coding
ENSMUSG00000019779	268.7458184	0.605148776	0.168204988	8.47E-06	5.90E-04	Frk	10:34483399-34611278	fyn-related kinase [Source:MGI Symbol;Acc:MGI:103265]	protein_coding
ENSMUSG00000036904	784.3453738	0.604983563	0.174075972	1.26E-05	7.75E-04	Fzd8	18:9212163-9218136	frizzled class receptor 8 [Source:MGI Symbol;Acc:MGI:108460]	protein_coding
ENSMUSG00000053399	231.8656686	0.604739388	0.326606071	8.63E-04	0.014366068	Adamts18	8:113697126-113848738	a disintegrin-like and metallopeptidase (reprolysin type) with thrombospondin type 1 motif, 18 [Sourc	protein_coding
ENSMUSG00000049804	828.2926325	0.604407288	0.111315374	3.56E-09	1.53E-06	Armcx4	X:134686519-134696757	armadillo repeat containing, X-linked 4 [Source:MGI Symbol;Acc:MGI:2147887]	protein_coding
ENSMUSG00000008575	3617.034566	0.604050786	0.222620299	1.16E-04	0.003755411	Nfib	4:82290173-82705750	nuclear factor I/B [Source:MGI Symbol;Acc:MGI:103188]	protein_coding
ENSMUSG00000057897	473.3083776	0.602918647	0.327616121	8.89E-04	0.014618171	Camk2b	11:5969644-6066362	calcium/calmodulin-dependent protein kinase II, beta [Source:MGI Symbol;Acc:MGI:88257]	protein_coding
ENSMUSG00000031434	229.6183328	0.602726158	0.155005863	3.10E-06	2.83E-04	Morc4	X:139821632-139871677	microrchidia 4 [Source:MGI Symbol;Acc:MGI:1922996]	protein_coding
ENSMUSG00000032012	529.4421494	0.601753206	0.212233289	8.41E-05	0.003028091	Nectin1	9:43743984-43832658	nectin cell adhesion molecule 1 [Source:MGI Symbol;Acc:MGI:1926483]	protein_coding
ENSMUSG00000022096	626.5505095	0.600780092	0.327961988	9.10E-04	0.014823086	Hr	14:70552212-70573548	lysine demethylase and nuclear receptor corepressor [Source:MGI Symbol;Acc:MGI:96223]	protein_coding
ENSMUSG00000021217	306.4791474	0.600046126	0.218686908	1.08E-04	0.003614228	Tshz3	7:36698118-36773553	teashirt zinc finger family member 3 [Source:MGI Symbol;Acc:MGI:2442819]	protein_coding
ENSMUSG00000022708	7170.9872	0.59980908	0.224096167	1.29E-04	0.004072994	Zbtb20	16:42875881-43642602	zinc finger and BTB domain containing 20 [Source:MGI Symbol;Acc:MGI:1929213]	protein_coding
ENSMUSG00000063160	857.7546404	0.598907846	0.142848633	9.89E-07	1.21E-04	Numbl	7:27258433-27282144	numb-like [Source:MGI Symbol;Acc:MGI:894702]	protein_coding
ENSMUSG00000017466	12400.51717	0.59861333	0.185251184	2.70E-05	0.001364003	Timp2	11:118301069-118355740	tissue inhibitor of metalloproteinase 2 [Source:MGI Symbol;Acc:MGI:98753]	protein_coding
ENSMUSG00000004151	243.7555639	0.598257238	0.216970498	1.04E-04	0.003529811	Etv1	12:38779380-38870484	ets variant 1 [Source:MGI Symbol;Acc:MGI:99254]	protein_coding
ENSMUSG00000020000	66.91424441	0.598036314	0.51895326	0.002297681	0.027482928	Moxd1	10:24223517-24302790	monooxygenase, DBH-like 1 [Source:MGI Symbol;Acc:MGI:1921582]	protein_coding
ENSMUSG00000068748	1847.343953	0.597987492	0.284371046	5.21E-04	0.010223401	Ptprz1	6:22875502-23052916	protein tyrosine phosphatase, receptor type Z, polypeptide 1 [Source:MGI Symbol;Acc:MGI:97816]	protein_coding
ENSMUSG00000023972	1409.883624	0.596763695	0.179547074	2.04E-05	0.001099009	Ptk7	17:46564471-46629504	PTK7 protein tyrosine kinase 7 [Source:MGI Symbol;Acc:MGI:1918711]	protein_coding
ENSMUSG00000000416	134.659333	0.596392571	0.24769585	2.56E-04	0.006504375	Cttnbp2	6:18366478-18514843	cortactin binding protein 2 [Source:MGI Symbol;Acc:MGI:1353467]	protein_coding
ENSMUSG00000009614	736.3620599	0.593130748	0.113184715	9.14E-09	3.51E-06	Sardh	2:27188393-27248337	sarcosine dehydrogenase [Source:MGI Symbol;Acc:MGI:2183102]	protein_coding
ENSMUSG00000020826	51.95865442	0.590234567	0.310544955	8.23E-04	0.013881693	Nos2	11:78920787-78960254	nitric oxide synthase 2, inducible [Source:MGI Symbol;Acc:MGI:97361]	protein_coding
ENSMUSG00000020251	107.3161427	0.590055477	0.300887618	7.26E-04	0.012653549	Glt8d2	10:82650433-82690650	glycosyltransferase 8 domain containing 2 [Source:MGI Symbol;Acc:MGI:1922032]	protein_coding
ENSMUSG00000091476	54.93318723	0.588765536	0.242502649	2.48E-04	0.006378899	Catspere2	1:177983423-178172705	cation channel sperm associated auxiliary subunit epsilon 2 [Source:MGI Symbol;Acc:MGI:5589632]	protein_coding
ENSMUSG00000021994	459.1993914	0.586921734	0.242576277	2.54E-04	0.006485534	Wnt5a	14:28504750-28527448	wingless-type MMTV integration site family, member 5A [Source:MGI Symbol;Acc:MGI:98958]	protein_coding
ENSMUSG00000055980	3546.259862	0.586600606	0.2175116	1.25E-04	0.003945387	Irs1	1:82233101-82291416	insulin receptor substrate 1 [Source:MGI Symbol;Acc:MGI:99454]	protein_coding
ENSMUSG00000061013	201.9257812	0.58489213	0.232968662	2.03E-04	0.005518506	Mkx	18:6934518-7004780	mohawk homeobox [Source:MGI Symbol;Acc:MGI:2687286]	protein_coding
ENSMUSG00000071478	1045.643392	0.58464861	0.56439491	0.002554904	0.029477801	H2ac7	13:23574381-23574952	H2A clustered histone 7 [Source:MGI Symbol;Acc:MGI:2448289]	protein_coding
ENSMUSG00000042870	682.6945508	0.584633821	0.436615345	0.002137778	0.026185885	Tom1	8:75033705-75070121	target of myb1 trafficking protein [Source:MGI Symbol;Acc:MGI:1338026]	protein_coding
ENSMUSG00000063954	128.6411363	0.583760474	0.626365674	0.002556136	0.029477801	H2ac19	3:96239749-96240276	H2A clustered histone 19 [Source:MGI Symbol;Acc:MGI:2448283]	protein_coding
ENSMUSG00000035274	607.1393814	0.583258063	0.259097659	3.86E-04	0.008461529	Tpbg	9:85842380-85847040	trophoblast glycoprotein [Source:MGI Symbol;Acc:MGI:1341264]	protein_coding
ENSMUSG00000027223	259.9947518	0.58257601	0.22500942	1.66E-04	0.004851395	Mapk8ip1	2:92383676-92401263	mitogen-activated protein kinase 8 interacting protein 1 [Source:MGI Symbol;Acc:MGI:1309464]	protein_coding
ENSMUSG00000034910	434.8252026	0.58168728	0.16861771	1.36E-05	8.07E-04	Pygo1	9:72925645-72952181	pygopus 1 [Source:MGI Symbol;Acc:MGI:1919385]	protein_coding
ENSMUSG00000034220	12176.95539	0.579258448	0.123348172	1.16E-07	2.30E-05	Gpc1	1:92831645-92860779	glypican 1 [Source:MGI Symbol;Acc:MGI:1194891]	protein_coding
ENSMUSG00000041482	211.0113638	0.579033098	0.324889212	0.001087631	0.016671804	Piezo2	18:63010213-63387183	piezo-type mechanosensitive ion channel component 2 [Source:MGI Symbol;Acc:MGI:1918781]	protein_coding
ENSMUSG00000028082	1678.185724	0.57875167	0.128156814	2.53E-07	4.22E-05	Sh3d19	3:85971109-86130526	SH3 domain protein D19 [Source:MGI Symbol;Acc:MGI:1350923]	protein_coding
ENSMUSG00000045103	1038.633032	0.57858438	0.469070175	0.002432817	0.028476196	Dmd	X:82948870-85206141	dystrophin, muscular dystrophy [Source:MGI Symbol;Acc:MGI:94909]	protein_coding
ENSMUSG00000073702	8071.061146	0.572167665	0.377758764	0.001799305	0.023543867	Rpl31	1:39367842-39371911	ribosomal protein L31 [Source:MGI Symbol;Acc:MGI:2149632]	protein_coding
ENSMUSG00000025938	211.1637366	0.572010612	0.286180089	7.09E-04	0.012424817	Slco5a1	1:12866549-12992650	solute carrier organic anion transporter family, member 5A1 [Source:MGI Symbol;Acc:MGI:2443431]	protein_coding
ENSMUSG00000034591	650.3584085	0.57133814	0.130635723	4.50E-07	6.56E-05	Slc41a2	10:83230848-83337882	solute carrier family 41, member 2 [Source:MGI Symbol;Acc:MGI:2442940]	protein_coding
ENSMUSG00000035041	160.4883193	0.569305233	0.228879926	2.21E-04	0.005847874	Creb3l3	10:81084324-81098874	cAMP responsive element binding protein 3-like 3 [Source:MGI Symbol;Acc:MGI:2384786]	protein_coding
ENSMUSG00000047819	133.8737747	0.568230712	0.538192151	0.002796209	0.031050842	Tigd4	3:84593574-84597032	tigger transposable element derived 4 [Source:MGI Symbol;Acc:MGI:2685264]	protein_coding
ENSMUSG00000035735	184.3127113	0.567607191	0.215044308	1.49E-04	0.004447653	Dagla	19:10245265-10304877	diacylglycerol lipase, alpha [Source:MGI Symbol;Acc:MGI:2677061]	protein_coding
ENSMUSG00000037375	152.6755784	0.567235555	0.156178234	7.29E-06	5.25E-04	Hhat	1:192496711-192771223	hedgehog acyltransferase [Source:MGI Symbol;Acc:MGI:2444681]	protein_coding
ENSMUSG00000089774	1533.80578	0.566920988	0.215653208	1.53E-04	0.004555447	Slc5a3	16:92058322-92087473	solute carrier family 5 (inositol transporters), member 3 [Source:MGI Symbol;Acc:MGI:1858226]	protein_coding
ENSMUSG00000020738	990.8333178	0.566670637	0.469963801	0.002650235	0.03011819	Sumo2	11:115523102-115536276	small ubiquitin-like modifier 2 [Source:MGI Symbol;Acc:MGI:2158813]	protein_coding
ENSMUSG00000052911	4860.127608	0.566517318	0.205975196	1.10E-04	0.003658217	Lamb2	9:108479736-108490530	laminin, beta 2 [Source:MGI Symbol;Acc:MGI:99916]	protein_coding
ENSMUSG00000021367	65.62255408	0.566137697	0.37744795	0.001892575	0.024327547	Edn1	13:42301476-42307990	endothelin 1 [Source:MGI Symbol;Acc:MGI:95283]	protein_coding
ENSMUSG00000028019	949.1775608	0.565816425	0.115464561	4.35E-08	1.19E-05	Pdgfc	3:81036416-81214040	platelet-derived growth factor, C polypeptide [Source:MGI Symbol;Acc:MGI:1859631]	protein_coding
ENSMUSG00000040562	425.2687157	0.564460052	0.207540268	1.20E-04	0.003849102	Gstm2	3:107981702-107986453	glutathione S-transferase, mu 2 [Source:MGI Symbol;Acc:MGI:95861]	protein_coding
ENSMUSG00000020176	2790.488658	0.563825959	0.156948868	8.31E-06	5.83E-04	Grb10	11:11930508-12038683	growth factor receptor bound protein 10 [Source:MGI Symbol;Acc:MGI:103232]	protein_coding
ENSMUSG00000079480	158.6012785	0.563420914	0.491179609	0.002793749	0.031045133	Pin4	X:102119447-102127669	protein (peptidyl-prolyl cis/trans isomerase) NIMA-interacting, 4 (parvulin) [Source:MGI Symbol;Acc:M	protein_coding
ENSMUSG00000026888	544.744499	0.563031476	0.159611512	1.03E-05	6.76E-04	Grb14	2:64912476-65024987	growth factor receptor bound protein 14 [Source:MGI Symbol;Acc:MGI:1355324]	protein_coding
ENSMUSG00000059824	1522.780925	0.562463364	0.304531236	0.001013445	0.015918811	Dbp	7:45705088-45710203	D site albumin promoter binding protein [Source:MGI Symbol;Acc:MGI:94866]	protein_coding
ENSMUSG00000042793	770.7018402	0.561512318	0.332730371	0.001396408	0.01982627	Lgr6	1:134983301-135105276	leucine-rich repeat-containing G protein-coupled receptor 6 [Source:MGI Symbol;Acc:MGI:2441805]	protein_coding
ENSMUSG00000025151	6136.202258	0.561119142	0.167921582	1.90E-05	0.00104542	Maged1	X:94535474-94542143	melanoma antigen, family D, 1 [Source:MGI Symbol;Acc:MGI:1930187]	protein_coding
ENSMUSG00000034377	2101.284324	0.560568321	0.243936002	3.63E-04	0.008110193	Tulp4	17:6106437-6251128	tubby like protein 4 [Source:MGI Symbol;Acc:MGI:1916092]	protein_coding
ENSMUSG00000027932	220.4759585	0.5603531	0.21134933	1.46E-04	0.00439824	Slc27a3	3:90385239-90389938	solute carrier family 27 (fatty acid transporter), member 3 [Source:MGI Symbol;Acc:MGI:1347358]	protein_coding
ENSMUSG00000027230	3526.458317	0.559720748	0.226570187	2.35E-04	0.006129879	Creb3l1	2:91982328-92024502	cAMP responsive element binding protein 3-like 1 [Source:MGI Symbol;Acc:MGI:1347062]	protein_coding
ENSMUSG00000078974	1511.52832	0.559159567	0.397354537	0.002238143	0.026947682	Sec61g	11:16500530-16508484	SEC61, gamma subunit [Source:MGI Symbol;Acc:MGI:1202066]	protein_coding
ENSMUSG00000035258	2223.097912	0.55739758	0.208514896	1.38E-04	0.004243025	Abi3bp	16:56477878-56690135	ABI gene family, member 3 (NESH) binding protein [Source:MGI Symbol;Acc:MGI:2444583]	protein_coding
ENSMUSG00000037621	257.9878196	0.557019315	0.191923229	7.23E-05	0.002725942	Atoh8	6:72206177-72235577	atonal bHLH transcription factor 8 [Source:MGI Symbol;Acc:MGI:1918343]	protein_coding
ENSMUSG00000029671	235.8753967	0.556407734	0.404338935	0.002363654	0.027940926	Wnt16	6:22288227-22298522	wingless-type MMTV integration site family, member 16 [Source:MGI Symbol;Acc:MGI:2136018]	protein_coding
ENSMUSG00000075702	1397.215152	0.555280766	0.144478058	3.38E-06	3.00E-04	Selenom	11:3514684-3517351	selenoprotein M [Source:MGI Symbol;Acc:MGI:2149786]	protein_coding
ENSMUSG00000044042	738.3025973	0.554268778	0.199609541	1.04E-04	0.003529811	Fmn1	2:113327736-113716767	formin 1 [Source:MGI Symbol;Acc:MGI:101815]	protein_coding
ENSMUSG00000031596	1376.211332	0.553755685	0.215979121	1.86E-04	0.005227465	Slc7a2	8:40862396-40922308	solute carrier family 7 (cationic amino acid transporter, y+ system), member 2 [Source:MGI Symbol;A	protein_coding
ENSMUSG00000034275	53.28487214	0.553615238	0.378101513	0.002119098	0.026039478	Igsf9b	9:27299204-27357546	immunoglobulin superfamily, member 9B [Source:MGI Symbol;Acc:MGI:2685354]	protein_coding
ENSMUSG00000036560	270.6933494	0.553360126	0.295538644	9.91E-04	0.015660992	Lgi4	7:31059342-31070935	leucine-rich repeat LGI family, member 4 [Source:MGI Symbol;Acc:MGI:2180197]	protein_coding
ENSMUSG00000053647	141.8577492	0.553348793	0.230241563	2.83E-04	0.006972283	Gper1	5:139423151-139427800	G protein-coupled estrogen receptor 1 [Source:MGI Symbol;Acc:MGI:1924104]	protein_coding
ENSMUSG00000021765	321.2976798	0.552874405	0.205988297	1.34E-04	0.004195452	Fst	13:114452290-114458951	follistatin [Source:MGI Symbol;Acc:MGI:95586]	protein_coding
ENSMUSG00000043613	106.6381437	0.552301094	0.630317251	0.00299045	0.03230543	Mmp3	9:7445822-7455975	matrix metallopeptidase 3 [Source:MGI Symbol;Acc:MGI:97010]	protein_coding
ENSMUSG00000019851	301.3800631	0.55222063	0.570803797	0.003067695	0.032831691	Perp	10:18845020-18857073	PERP, TP53 apoptosis effector [Source:MGI Symbol;Acc:MGI:1929938]	protein_coding
ENSMUSG00000024501	5683.196481	0.551264335	0.191556014	7.79E-05	0.002866541	Dpysl3	18:43320984-43438286	dihydropyrimidinase-like 3 [Source:MGI Symbol;Acc:MGI:1349762]	protein_coding
ENSMUSG00000030768	759.1868478	0.550912656	0.116177336	8.49E-08	1.79E-05	Disp1	1:183086266-183221522	dispatched RND transporter family member 1 [Source:MGI Symbol;Acc:MGI:1916147]	protein_coding
ENSMUSG00000062542	175.0620941	0.550839902	0.19846443	1.04E-04	0.003529811	Syt9	7:107370728-107548656	synaptotagmin IX [Source:MGI Symbol;Acc:MGI:1926373]	protein_coding
ENSMUSG00000048264	1125.39419	0.54963288	0.150596454	6.68E-06	4.88E-04	Dip2c	13:9276528-9668928	disco interacting protein 2 homolog C [Source:MGI Symbol;Acc:MGI:1920179]	protein_coding

ENSMUSG00000068130	56.88296429	0.549504546	0.264017873	6.28E-04	0.011477533	Zfp442	2:150407141-150451486	zinc finger protein 442 [Source:MGI Symbol;Acc:MGI:3651999]	protein_coding
ENSMUSG00000028226	991.1483891	0.549503315	0.267052654	6.64E-04	0.011966807	Mmp16	4:17852893-18119145	matrix metallopeptidase 16 [Source:MGI Symbol;Acc:MGI:1276107]	protein_coding
ENSMUSG00000032011	801.2184008	0.549323809	0.198817504	1.08E-04	0.003614228	Thy1	9:44043384-44048579	thymus cell antigen 1, theta [Source:MGI Symbol;Acc:MGI:98747]	protein_coding
ENSMUSG00000029851	148.6324714	0.549115798	0.1812135	4.97E-05	0.002118941	Tcaf2	6:42623016-42645254	TRPM8 channel-associated factor 2 [Source:MGI Symbol;Acc:MGI:2385258]	protein_coding
ENSMUSG00000046152	260.3726907	0.548993295	0.147346433	5.10E-06	3.98E-04	Fut10	8:31187331-31261738	fucosyltransferase 10 [Source:MGI Symbol;Acc:MGI:2384748]	protein_coding
ENSMUSG00000061983	13643.08938	0.548195244	0.394819479	0.002423822	0.028401322	Rps12	10:23785183-23787275	ribosomal protein S12 [Source:MGI Symbol;Acc:MGI:98105]	protein_coding
ENSMUSG00000022987	65.17511215	0.547797416	0.460281127	0.002978978	0.032213332	Zfp641	15:98285585-98296161	zinc finger protein 641 [Source:MGI Symbol;Acc:MGI:2442788]	protein_coding
ENSMUSG00000022602	92.47530918	0.547456135	0.267114868	6.80E-04	0.0120966	Arc	15:74669083-74672570	activity regulated cytoskeletal-associated protein [Source:MGI Symbol;Acc:MGI:88067]	protein_coding
ENSMUSG00000045790	180.7390018	0.547342096	0.160977905	1.55E-05	9.08E-04	Ccdc149	5:52374651-52471521	coiled-coil domain containing 149 [Source:MGI Symbol;Acc:MGI:2685293]	protein_coding
ENSMUSG00000079435	2847.731255	0.547166049	0.411013478	0.002618606	0.029961186	Rpl36a	X:134585654-134588062	ribosomal protein L36A [Source:MGI Symbol;Acc:MGI:1201789]	protein_coding
ENSMUSG00000001119	11066.59487	0.547002937	0.195332385	9.75E-05	0.003378567	Col6a1	10:76708792-76726168	collagen, type VI, alpha 1 [Source:MGI Symbol;Acc:MGI:88459]	protein_coding
ENSMUSG00000094248	138.5377317	0.546553442	0.622965257	0.003090408	0.032961836	Hist1h2ao	13:21810465-21810944	histone cluster 1, H2ao [Source:MGI Symbol;Acc:MGI:2448302]	protein_coding
ENSMUSG00000047215	7900.834379	0.546262582	0.39351476	0.002448407	0.028638185	Rpl9	5:65388364-65391444	ribosomal protein L9 [Source:MGI Symbol;Acc:MGI:1298373]	protein_coding
ENSMUSG00000069308	731.9068107	0.54603014	0.545287727	0.003192609	0.033738237	Hist1h2bp	13:21787461-21789213	histone cluster 1, H2bp [Source:MGI Symbol;Acc:MGI:2448409]	protein_coding
ENSMUSG00000079111	4925.483013	0.545298334	0.084616288	7.77E-12	7.39E-09	Kdelr2	5:143403838-143421901	KDEL (Lys-Asp-Glu-Leu) endoplasmic reticulum protein retention receptor 2 [Source:MGI Symbol;Acc:	protein_coding
ENSMUSG00000002771	189.5801493	0.545285669	0.26612504	6.86E-04	0.01214749	Grin2d	7:45831883-45878378	glutamate receptor, ionotropic, NMDA2D (epsilon 4) [Source:MGI Symbol;Acc:MGI:95823]	protein_coding
ENSMUSG00000047033	56.39188058	0.544675733	0.217335852	2.20E-04	0.005819841	Pcdhb15	18:37473540-37476340	protocadherin beta 15 [Source:MGI Symbol;Acc:MGI:2136750]	protein_coding
ENSMUSG00000029304	60946.51849	0.541773031	0.31515067	0.001419256	0.020033591	Spp1	5:104435118-104441050	secreted phosphoprotein 1 [Source:MGI Symbol;Acc:MGI:98389]	protein_coding
ENSMUSG00000058571	664.2031294	0.540925491	0.266945647	7.32E-04	0.012696182	Gpc6	14:116925315-117976544	glypican 6 [Source:MGI Symbol;Acc:MGI:1346322]	protein_coding
ENSMUSG00000045045	349.8274481	0.540489592	0.139557983	2.93E-06	2.75E-04	Lrfn4	19:4611785-4615667	leucine rich repeat and fibronectin type III domain containing 4 [Source:MGI Symbol;Acc:MGI:238561	protein_coding
ENSMUSG00000040428	228.6656369	0.540194661	0.281679834	9.38E-04	0.015126039	Plekha4	7:45526330-45554229	pleckstrin homology domain containing, family A (phosphoinositide binding specific) member 4 [Sourc	protein_coding
ENSMUSG00000019577	2223.608702	0.540138448	0.428276164	0.002931971	0.031995874	Pdk4	6:5483351-5496309	pyruvate dehydrogenase kinase, isoenzyme 4 [Source:MGI Symbol;Acc:MGI:1351481]	protein_coding
ENSMUSG00000039646	2691.080288	0.539960899	0.210878434	1.91E-04	0.005327846	Vasn	16:4639941-4650802	vasorin [Source:MGI Symbol;Acc:MGI:2177651]	protein_coding
ENSMUSG00000005973	2776.71891	0.539597983	0.127473443	7.23E-07	9.40E-05	Rcn1	2:105386291-105399319	reticulocalbin 1 [Source:MGI Symbol;Acc:MGI:104559]	protein_coding
ENSMUSG00000000142	357.0981502	0.539423473	0.211491463	1.96E-04	0.005428191	Axin2	11:108920349-108950783	axin 2 [Source:MGI Symbol;Acc:MGI:1270862]	protein_coding
ENSMUSG00000030319	506.8153044	0.537505249	0.302405816	0.001280552	0.018678638	Cand2	6:115774538-115805557	cullin-associated and neddylation-dissociated 2 (putative) [Source:MGI Symbol;Acc:MGI:1914338]	protein_coding
ENSMUSG00000018920	373.3620451	0.534477765	0.186852684	8.26E-05	0.002998769	Cxcl16	11:70453983-70459984	chemokine (C-X-C motif) ligand 16 [Source:MGI Symbol;Acc:MGI:1932682]	protein_coding
ENSMUSG00000029673	794.3571053	0.533977658	0.192069488	1.04E-04	0.003529811	Auts2	5:131437333-132543344	autism susceptibility candidate 2 [Source:MGI Symbol;Acc:MGI:1919847]	protein_coding
ENSMUSG00000027983	239.2887303	0.533883134	0.234433786	4.05E-04	0.008629558	Cyp2u1	3:131288441-131303227	cytochrome P450, family 2, subfamily u, polypeptide 1 [Source:MGI Symbol;Acc:MGI:1918769]	protein_coding
ENSMUSG00000026193	58264.27987	0.532888543	0.248187914	5.61E-04	0.010747448	Fn1	1:71585520-71653200	fibronectin 1 [Source:MGI Symbol;Acc:MGI:95566]	protein_coding
ENSMUSG00000025432	62.73200166	0.529516661	0.330814603	0.001873891	0.024215504	Avil	10:127000709-127020994	advillin [Source:MGI Symbol;Acc:MGI:1333798]	protein_coding
ENSMUSG00000020695	6923.170251	0.528154917	0.225179505	3.44E-04	0.007885785	Mrc2	11:105292643-105351139	mannose receptor, C type 2 [Source:MGI Symbol;Acc:MGI:107818]	protein_coding
ENSMUSG00000035325	7340.477841	0.527313252	0.10135702	8.27E-09	3.23E-06	Sec31a	5:100361649-100416234	Sec31 homolog A (S. cerevisiae) [Source:MGI Symbol;Acc:MGI:1916412]	protein_coding
ENSMUSG00000051934	282.5164837	0.526964886	0.15181276	1.19E-05	7.51E-04	Spats2	15:99125916-99213215	spermatogenesis associated, serine-rich 2 [Source:MGI Symbol;Acc:MGI:1919822]	protein_coding
ENSMUSG00000023411	651.1132758	0.525907656	0.229028121	3.93E-04	0.008532298	Nfatc4	14:55823144-55833943	nuclear factor of activated T cells, cytoplasmic, calcineurin dependent 4 [Source:MGI Symbol;Acc:MGI	protein_coding
ENSMUSG00000032238	1239.799329	0.52521878	0.268793741	9.06E-04	0.014812833	Rora	9:68653786-69388246	RAR-related orphan receptor alpha [Source:MGI Symbol;Acc:MGI:104661]	protein_coding
ENSMUSG00000038900	11250.81576	0.52518196	0.358295623	0.002417303	0.028340124	Rpl12	2:32961559-32965345	ribosomal protein L12 [Source:MGI Symbol;Acc:MGI:98002]	protein_coding
ENSMUSG00000028369	2119.327416	0.524598044	0.236454916	4.80E-04	0.009713219	Svep1	4:58042442-58206859	sushi, von Willebrand factor type A, EGF and pentraxin domain containing 1 [Source:MGI Symbol;Acc:	protein_coding
ENSMUSG00000028919	516.1296962	0.524052073	0.244900058	5.84E-04	0.010982163	Arhgef19	4:141239499-141257564	Rho guanine nucleotide exchange factor (GEF) 19 [Source:MGI Symbol;Acc:MGI:1925912]	protein_coding
ENSMUSG00000022203	729.2160646	0.524050221	0.217272564	2.92E-04	0.007104642	Efs	14:54916535-54926126	embryonal Fyn-associated substrate [Source:MGI Symbol;Acc:MGI:105311]	protein_coding
ENSMUSG00000073530	129.4708868	0.523940082	0.32339983	0.001848947	0.023988393	Pappa2	1:158711727-158980490	pappalysin 2 [Source:MGI Symbol;Acc:MGI:3051647]	protein_coding
ENSMUSG00000020186	553.8252056	0.52245746	0.137254373	3.58E-06	3.09E-04	Csrp2	10:110919789-110939622	cysteine and glycine-rich protein 2 [Source:MGI Symbol;Acc:MGI:1202907]	protein_coding
ENSMUSG00000032064	394.0633372	0.52142046	0.244815146	6.03E-04	0.01117716	Dixdc1	9:50662752-50739517	DIX domain containing 1 [Source:MGI Symbol;Acc:MGI:2679721]	protein_coding
ENSMUSG00000038143	975.3634623	0.520929684	0.208191995	2.30E-04	0.006020059	Stox2	8:47180048-47446362	storkhead box 2 [Source:MGI Symbol;Acc:MGI:1918319]	protein_coding
ENSMUSG00000022449	64.79516714	0.520867268	0.478267416	0.003677957	0.036620461	Adamts20	15:94270163-94465418	a disintegrin-like and metallopeptidase (reprolysin type) with thrombospondin type 1 motif, 20 [Sourc	protein_coding
ENSMUSG00000032925	751.6722486	0.520478444	0.422803286	0.003366366	0.034681166	Itgbl1	14:123659971-123975618	integrin, beta-like 1 [Source:MGI Symbol;Acc:MGI:2443439]	protein_coding
ENSMUSG00000020135	59.39783669	0.520108264	0.238032073	5.27E-04	0.0103232	Apc2	10:80295977-80318263	APC regulator of WNT signaling pathway 2 [Source:MGI Symbol;Acc:MGI:1346052]	protein_coding
ENSMUSG00000058317	294.5325983	0.520094435	0.160057883	2.47E-05	0.001276001	Ube2e2	14:18573575-18894267	ubiquitin-conjugating enzyme E2E 2 [Source:MGI Symbol;Acc:MGI:2384997]	protein_coding
ENSMUSG00000029767	10043.74969	0.519917611	0.100662196	9.56E-09	3.60E-06	Calu	6:29348069-29377110	calumenin [Source:MGI Symbol;Acc:MGI:1097158]	protein_coding
ENSMUSG00000051855	441.6839209	0.519675594	0.337541521	0.002185607	0.026446211	Mest	6:30723547-30748465	mesoderm specific transcript [Source:MGI Symbol;Acc:MGI:96968]	protein_coding
ENSMUSG00000003352	845.5758522	0.517770901	0.213166745	2.82E-04	0.006962035	Cacnb3	15:98630840-98644529	calcium channel, voltage-dependent, beta 3 subunit [Source:MGI Symbol;Acc:MGI:103307]	protein_coding
ENSMUSG00000049872	172.8974325	0.517518147	0.251185518	7.21E-04	0.012576453	Calhm5	10:34087815-34096519	calcium homeostasis modulator family member 5 [Source:MGI Symbol;Acc:MGI:2143897]	protein_coding
ENSMUSG00000021719	139.0373067	0.517174533	0.266321003	9.55E-04	0.015298119	Rgs7bp	13:104945904-105054930	regulator of G-protein signalling 7 binding protein [Source:MGI Symbol;Acc:MGI:106334]	protein_coding
ENSMUSG00000035407	302.3815551	0.516939	0.219817939	3.46E-04	0.007906895	Kank4	4:98754898-98817537	KN motif and ankyrin repeat domains 4 [Source:MGI Symbol;Acc:MGI:3043381]	protein_coding
ENSMUSG00000022178	232.4028484	0.516629588	0.174189028	6.04E-05	0.002410659	Ajuba	14:54567472-54577558	ajuba LIM protein [Source:MGI Symbol;Acc:MGI:1341886]	protein_coding
ENSMUSG00000034435	177.340554	0.516429289	0.185558161	1.04E-04	0.003529811	Tmem30b	12:73543402-73546392	transmembrane protein 30B [Source:MGI Symbol;Acc:MGI:2442082]	protein_coding
ENSMUSG00000029122	557.1564191	0.516378937	0.190958933	1.31E-04	0.004131062	Evc	5:37289098-37336894	EvC ciliary complex subunit 1 [Source:MGI Symbol;Acc:MGI:1890596]	protein_coding
ENSMUSG00000048388	278.5791082	0.515724833	0.238105806	5.59E-04	0.01073064	Fam171b	2:83812636-83883486	family with sequence similarity 171, member B [Source:MGI Symbol;Acc:MGI:2444579]	protein_coding
ENSMUSG00000022150	2396.531313	0.515666293	0.164976388	3.67E-05	0.001689599	Dab2	15:6299788-6440712	disabled 2, mitogen-responsive phosphoprotein [Source:MGI Symbol;Acc:MGI:109175]	protein_coding
ENSMUSG00000031642	995.431616	0.513277933	0.189499365	1.30E-04	0.004093839	Sh3rf1	8:61223872-61396071	SH3 domain containing ring finger 1 [Source:MGI Symbol;Acc:MGI:1913066]	protein_coding
ENSMUSG00000024529	11439.80397	0.513029672	0.218219223	3.49E-04	0.007948203	Lox	18:52516067-52529867	lysyl oxidase [Source:MGI Symbol;Acc:MGI:96817]	protein_coding
ENSMUSG00000037370	3037.288359	0.51302532	0.239518618	5.97E-04	0.011118213	Enpp1	10:24637914-24712159	ectonucleotide pyrophosphatase/phosphodiesterase 1 [Source:MGI Symbol;Acc:MGI:97370]	protein_coding
ENSMUSG00000024420	1221.976684	0.512304669	0.19007913	1.35E-04	0.004209564	Zfp521	18:13687013-13972787	zinc finger protein 521 [Source:MGI Symbol;Acc:MGI:95459]	protein_coding
ENSMUSG00000048612	1848.963711	0.51136874	0.150104788	1.45E-05	8.54E-04	Myof	19:37899036-38043577	myoferlin [Source:MGI Symbol;Acc:MGI:1919192]	protein_coding
ENSMUSG00000049047	424.2913239	0.510737311	0.124641014	1.12E-06	1.32E-04	Armcx3	X:134756595-134761455	armadillo repeat containing, X-linked 3 [Source:MGI Symbol;Acc:MGI:1918953]	protein_coding
ENSMUSG00000019975	1328.097319	0.510586779	0.145607665	1.03E-05	6.74E-04	Ikbip	10:91082940-91102607	IKBKB interacting protein [Source:MGI Symbol;Acc:MGI:1914704]	protein_coding
ENSMUSG00000067889	98.53977777	0.509942162	0.363939008	0.002879421	0.031621567	Sptbn2	19:4711167-4752360	spectrin beta, non-erythrocytic 2 [Source:MGI Symbol;Acc:MGI:1313261]	protein_coding
ENSMUSG00000033453	415.6995248	0.509832061	0.240477716	6.36E-04	0.011566667	Adamts15	9:30899155-30922452	a disintegrin-like and metallopeptidase (reprolysin type) with thrombospondin type 1 motif, 15 [Sourc	protein_coding
ENSMUSG00000036446	12900.63735	0.509259799	0.325699366	0.002189011	0.026458111	Lum	10:97565128-97572703	lumican [Source:MGI Symbol;Acc:MGI:109347]	protein_coding
ENSMUSG00000002732	1221.329804	0.509254984	0.251286244	8.00E-04	0.01356811	Fkbp7	2:76663044-76673116	FK506 binding protein 7 [Source:MGI Symbol;Acc:MGI:1336879]	protein_coding
ENSMUSG00000028128	133.7504847	0.50914214	0.272393643	0.001157534	0.017382891	F3	3:121723537-121735048	coagulation factor III [Source:MGI Symbol;Acc:MGI:88381]	protein_coding
ENSMUSG00000025586	327.137389	0.509070412	0.322273728	0.002125619	0.026095818	Cpeb1	7:81347026-81455465	cytoplasmic polyadenylation element binding protein 1 [Source:MGI Symbol;Acc:MGI:108442]	protein_coding
ENSMUSG00000051041	223.2935953	0.509034448	0.252072194	8.15E-04	0.013781615	Olfml1	7:107567446-107591094	olfactomedin-like 1 [Source:MGI Symbol;Acc:MGI:2679264]	protein_coding
ENSMUSG00000058152	412.8773611	0.507947042	0.135732911	4.37E-06	3.55E-04	Chsy3	18:59175401-59535999	chondroitin sulfate synthase 3 [Source:MGI Symbol;Acc:MGI:1926173]	protein_coding
ENSMUSG00000018906	861.5120461	0.507479904	0.233223477	5.56E-04	0.010691212	P4ha2	11:54100095-54131665	procollagen-proline, 2-oxoglutarate 4-dioxygenase (proline 4-hydroxylase), alpha II polypeptide [Sourc	protein_coding
ENSMUSG00000014329	2771.702693	0.506079972	0.156130596	2.50E-05	0.001288254	Bicc1	10:70922832-71159700	BicC family RNA binding protein 1 [Source:MGI Symbol;Acc:MGI:1933388]	protein_coding
ENSMUSG00000038872	3683.408489	0.505878504	0.262838069	0.00102952	0.016068155	Zfhx3	8:107942644-108961630	zinc finger homeobox 3 [Source:MGI Symbol;Acc:MGI:99948]	protein_coding
ENSMUSG00000038260	528.9485975	0.505815356	0.142712064	8.89E-06	6.10E-04	Trpm4	7:45302632-45333780	transient receptor potential cation channel, subfamily M, member 4 [Source:MGI Symbol;Acc:MGI:19	protein_coding
ENSMUSG00000020260	2057.278714	0.503617107	0.098163531	1.02E-08	3.79E-06	Pofut2	10:77259218-77269575	protein O-fucosyltransferase 2 [Source:MGI Symbol;Acc:MGI:1916863]	protein_coding
ENSMUSG00000031137	179.3394378	0.503366159	0.184591718	1.24E-04	0.003936639	Fgf13	X:59062145-59568071	fibroblast growth factor 13 [Source:MGI Symbol;Acc:MGI:109178]	protein_coding
ENSMUSG00000046480	1130.392499	0.50297088	0.456311314	0.004086265	0.03899877	Scn4b	9:45138395-45154152	sodium channel, type IV, beta [Source:MGI Symbol;Acc:MGI:2687406]	protein_coding
ENSMUSG00000037656	2707.085936	0.502072853	0.13856603	6.65E-06	4.88E-04	Slc20a2	8:22476788-22569612	solute carrier family 20, member 2 [Source:MGI Symbol;Acc:MGI:97851]	protein_coding
ENSMUSG00000036095	52.11768788	0.50177362	0.401104573	0.003664922	0.03659236	Dgkb	12:37817726-38634239	diacylglycerol kinase, beta [Source:MGI Symbol;Acc:MGI:2442474]	protein_coding
ENSMUSG00000015709	185.3348616	0.501333949	0.216610635	3.93E-04	0.008532298	Arnt2	7:84246278-84410176	aryl hydrocarbon receptor nuclear translocator 2 [Source:MGI Symbol;Acc:MGI:107188]	protein_coding
ENSMUSG00000057766	167.2062874	0.500225364	0.239463895	7.04E-04	0.012387717	Ankrd29	18:12252362-12305797	ankyrin repeat domain 29 [Source:MGI Symbol;Acc:MGI:2687055]	protein_coding
ENSMUSG00000014776	256.870142	0.500166702	0.313080902	0.002131889	0.02614344	Nol3	8:105276140-105281939	nucleolar protein 3 (apoptosis repressor with CARD domain) [Source:MGI Symbol;Acc:MGI:1925938]	protein_coding
ENSMUSG00000022484	312.8040177	0.499507281	0.258970029	0.001039977	0.016184417	Hoxc10	15:102966796-102971893	homeobox C10 [Source:MGI Symbol;Acc:MGI:96192]	protein_coding
ENSMUSG00000031398	314.4169813	0.49934453	0.191620564	1.77E-04	0.005037191	Plxna3	X:74329066-74344689	plexin A3 [Source:MGI Symbol;Acc:MGI:107683]	protein_coding
ENSMUSG00000031825	2278.248119	0.499275414	0.1523303	2.20E-05	0.001161117	Crispld2	8:119992438-120052793	cysteine-rich secretory protein LCCL domain containing 2 [Source:MGI Symbol;Acc:MGI:1926142]	protein_coding
ENSMUSG00000031963	280.2003546	0.499274946	0.218955697	4.32E-04	0.009069879	Bmper	9:23223076-23485200	BMP-binding endothelial regulator [Source:MGI Symbol;Acc:MGI:1920480]	protein_coding
ENSMUSG00000002900	2095.231309	0.499054196	0.167899357	5.91E-05	0.00238885	Lamb1	12:31265234-31329644	laminin B1 [Source:MGI Symbol;Acc:MGI:96743]	protein_coding
ENSMUSG00000002020	3634.002285	0.498684502	0.145027507	1.27E-05	7.76E-04	Ltbp2	12:84783212-84876532	latent transforming growth factor beta binding protein 2 [Source:MGI Symbol;Acc:MGI:99502]	protein_coding
ENSMUSG00000033565	2216.582685	0.497785095	0.188625272	1.61E-04	0.004722237	Rbfox2	15:77078990-77307004	RNA binding protein, fox-1 homolog (C. elegans) 2 [Source:MGI Symbol;Acc:MGI:1933973]	protein_coding
ENSMUSG00000006958	196.1910885	0.496474671	0.294554555	0.00180994	0.023641716	Chrd	16:20733127-20742384	chordin [Source:MGI Symbol;Acc:MGI:1313268]	protein_coding
ENSMUSG00000002908	174.4133965	0.495534792	0.251687623	9.57E-04	0.015313631	Kcnn1	8:70842049-70863258	potassium intermediate/small conductance calcium-activated channel, subfamily N, member 1 [Sourc	protein_coding
ENSMUSG00000025268	1440.138312	0.495512272	0.127518419	2.44E-06	2.40E-04	Maged2	X:150806370-150814345	melanoma antigen, family D, 2 [Source:MGI Symbol;Acc:MGI:1933391]	protein_coding
ENSMUSG00000073591	159.1959519	0.49536849	0.210179746	3.55E-04	0.00796787	Pcdhb22	18:37517871-37524479	protocadherin beta 22 [Source:MGI Symbol;Acc:MGI:2136760]	protein_coding
ENSMUSG00000103585	164.3733826	0.495131234	0.32766598	0.002566843	0.029519232	Pcdhgb4	18:37720369-37841870	protocadherin gamma subfamily B, 4 [Source:MGI Symbol;Acc:MGI:1935173]	protein_coding
ENSMUSG00000054545	62.29273502	-0.503506838	0.210126148	3.15E-04	0.007415045	Ugt1a6a	1:88134809-88218997	UDP glucuronosyltransferase 1 family, polypeptide A6A [Source:MGI Symbol;Acc:MGI:2137698]	protein_coding
ENSMUSG00000027556	8132.667768	-0.510644268	0.177301299	7.84E-05	0.002882277	Car1	3:14766216-14808368	carbonic anhydrase 1 [Source:MGI Symbol;Acc:MGI:88268]	protein_coding
ENSMUSG00000058183	66.10189835	-0.512544139	0.272812913	0.001120037	0.016947826	Mmel1	4:154869585-154895528	membrane metallo-endopeptidase-like 1 [Source:MGI Symbol;Acc:MGI:1351603]	protein_coding
ENSMUSG00000079597	170.4224912	-0.513482458	0.263987553	9.59E-04	0.015325826	Cstdc4	16:36184212-36188111	cystatin domain containing 4 [Source:MGI Symbol;Acc:MGI:3645124]	protein_coding
ENSMUSG00000067235	615.2580647	-0.520558882	0.185403917	9.70E-05	0.003364951	H2-Q10	17:35470089-35474563	histocompatibility 2, Q region locus 10 [Source:MGI Symbol;Acc:MGI:95929]	protein_coding
ENSMUSG00000049526	383.4035799	-0.5290675	0.379714665	0.002649088	0.03011819	Tmem202	9:59518685-59539847	transmembrane protein 202 [Source:MGI Symbol;Acc:MGI:1921143]	protein_coding
ENSMUSG00000050377	100.4399512	-0.534813794	0.125250357	6.11E-07	8.15E-05	Il31ra	13:112519898-112594360	interleukin 31 receptor A [Source:MGI Symbol;Acc:MGI:2180511]	protein_coding
ENSMUSG00000059201	83.9215781	-0.544841095	0.711624174	0.002942056	0.032037128	Lep	6:29060220-29073877	leptin [Source:MGI Symbol;Acc:MGI:104663]	protein_coding
ENSMUSG00000019359	2365.831013	-0.562534121	0.238681821	3.12E-04	0.00740129	Gdpd2	X:100729873-100738895	glycerophosphodiester phosphodiesterase domain containing 2 [Source:MGI Symbol;Acc:MGI:19188	protein_coding
ENSMUSG00000031738	53.39861885	-0.565325855	0.43986878	0.002499243	0.029036106	Irx6	8:92674288-92680956	Iroquois homeobox 6 [Source:MGI Symbol;Acc:MGI:1927642]	protein_coding
ENSMUSG00000020805	3272.513944	-0.565953714	0.24076694	3.15E-04	0.007415045	Slc13a5	11:72241989-72267222	solute carrier family 13 (sodium-dependent citrate transporter), member 5 [Source:MGI Symbol;Acc:M	protein_coding
ENSMUSG00000049565	95.55842464	-0.576390721	0.192758665	5.56E-05	0.002283309	Aknad1	3:108739658-108782309	AKNA domain containing 1 [Source:MGI Symbol;Acc:MGI:3584453]	protein_coding
ENSMUSG00000021758	62.25367893	-0.581251182	0.241163961	2.63E-04	0.006605813	Ddx4	13:112598333-112652475	DEAD (Asp-Glu-Ala-Asp) box polypeptide 4 [Source:MGI Symbol;Acc:MGI:102670]	protein_coding
ENSMUSG00000002324	173.947651	-0.634953785	0.276442693	3.04E-04	0.007261701	Rec8	14:55618037-55625395	REC8 meiotic recombination protein [Source:MGI Symbol;Acc:MGI:1929645]	protein_coding
ENSMUSG00000038540	99.39059202	-0.65871804	0.225269957	6.13E-05	0.002423155	Tmc3	7:83584927-83625614	transmembrane channel-like gene family 3 [Source:MGI Symbol;Acc:MGI:2669033]	protein_coding
ENSMUSG00000067786	74.48207538	-0.674725799	0.750852513	0.001651684	0.022237729	Nnat	2:157560078-157562522	neuronatin [Source:MGI Symbol;Acc:MGI:104716]	protein_coding
ENSMUSG00000021991	141.4720672	-0.679747977	0.258133348	1.23E-04	0.003931061	Cacna2d3	14:28904943-29721864	calcium channel, voltage-dependent, alpha2/delta subunit 3 [Source:MGI Symbol;Acc:MGI:1338890]	protein_coding
ENSMUSG00000044288	153.0394356	-0.702913397	0.255546515	8.78E-05	0.003106113	Cnr1	4:33924593-33948831	cannabinoid receptor 1 (brain) [Source:MGI Symbol;Acc:MGI:104615]	protein_coding
ENSMUSG00000059741	93.5851532	-0.81691612	0.77183382	9.03E-04	0.014808182	Myl3	9:110741861-110769798	myosin, light polypeptide 3 [Source:MGI Symbol;Acc:MGI:97268]	protein_coding
