# Supplementary material for: Targeting TGF‐β signaling, oxidative stress, and cellular senescence rescues osteoporosis in gerodermia osteodysplastica
Source: Aging Cell. 2024 Sep 5;23(12):e14322. doi: 10.1111/acel.14322 (PMC11634742; doi:10.1111/acel.14322)
Supplement: Supplementary file 3 — Table S2. [file ACEL-23-e14322-s003.pdf]

Supplementary Table 2. List of antibodies used in bmMSCs flow analysis

Marker	Clone	Manufacturer	RRID
CD45	30-F11	BioLegend, San Diego, CA, USA	AB_2563061
CD34	RAM34	BD Biosciences, Franklin Lakes, NJ, USA	AB_11154576
CD11b	M1/70	ThermoFisher, Waltham, MA, USA	AB_657585
CD31	390	BioLegend, San Diego, CA, USA	AB_2566207
CD29	HMB1-1	BioLegend, San Diego, CA, USA	AB_528790
CD44	IM7	BD Biosciences, Franklin Lakes, NJ, USA	AB_1727481
CD51	RMV-7	BioLegend, San Diego, CA, USA	AB_2129493
CD140a	APA5	BioLegend, San Diego, CA, USA	AB_2043970
CD24	M1/69	BioLegend, San Diego, CA, USA	AB_493482
Sca-1	D7	BD Biosciences, Franklin Lakes, NJ, USA	AB_2738529
